# DNA hypomethylation enables the transcriptional repressor SlSPL-CNR to control fruit flavor ester biosynthesis

**DOI:** 10.1038/s41467-026-75181-8

**Published:** 2026-07-01

**Authors:** Zhifeng Zeng, Yu Ma, Hang He, Linzhu Li, Yuan Peng, Yawen Hou, Songge Chai, Pengcheng Wang, Cheng-Guo Duan, Jian-Kang Zhu, Jiamu Du, Zhaobo Lang

**Affiliations:** 1 Institute of Advanced Biotechnology and School of Medicine, Southern University of Science and Technology, Shenzhen, China; 2 Shanghai Center for Plant Stress Biology and CAS Center for Excellence in Molecular Plant Sciences, Chinese Academy of Sciences, Shanghai, China; 3 University of Chinese Academy of Sciences, Beijing, China; 4 Shenzhen Key Laboratory of Plant Genetic Engineering and Molecular Design, Institute of Plant and Food Science and Institute for Biological Electron Microscopy, Department of Biology, School of Life Sciences, Southern University of Science and Technology, Shenzhen, China

**Keywords:** Structural biology, DNA methylation, Plant genetics, Plant development

## Abstract

Reduction of DNA methylation has traditionally been associated with gene activation. Here, we show that DNA hypomethylation permits the binding of a transcriptional repressor, leading to gene silencing. In tomato, the SQUAMOSA PROMOTER BINDING PROTEIN-LIKE TF SlSPL-CNR exhibits methylation-sensitive DNA binding and preferentially occupies unmethylated GTACGG motifs. During fruit ripening, DEMETER-LIKE 2 (SlDML2)-mediated DNA demethylation at the *alcohol acyltransferase 1* (*SlAAT1*) promoter allows SlSPL-CNR binding, which in turn represses *SlAAT1* expression and thereby modulates the biosynthesis of ester metabolites—key components of fruit flavor. Structural analysis reveals that cytosine methylation introduces a steric clash with Gln94 in the SBP domain of SlSPL-CNR, explaining its methylation sensitivity. CRISPR knockout of *SlSPL-CNR* de-represses *SlAAT1* and increases ester accumulation, confirming its inhibitory role. Importantly, this methylation-sensitive binding is conserved across SBP domain proteins from rice, maize, and tomato. Our findings reveal a mechanism in which DNA hypomethylation facilitates repressor recruitment, establishing a regulatory logic linking epigenetic dynamics to metabolic control in plants.

## Abstract (web summary)

Reduced DNA methylation is typically associated with transcriptional activation. Here, the authors show that SlDML2-mediated hypomethylation of the *SlAAT1* promoter instead facilitates binding of the repressor SlSPL-CNR, establishing a negative feedback loop that fine-tunes flavor ester accumulation in tomato.

## Introduction

Genomic DNA methylation, a conserved and well-studied epigenetic modification, involves the covalent addition of a methyl group (–CH₃) to the fifth carbon atom of cytosine (5-mC)^1,2^. Genomic DNA methylation homeostasis is maintained through the dynamic regulation and antagonistic interactions between DNA methylation and demethylation^3,4^. In mammals, 5-mC mainly occurs within CG dinucleotide sequences and is methylated by DNA methyltransferases (DNMTs)^5^. In contrast, DNA methylation in plants can occur in all cytosine contexts, including symmetrical (CG, CHG; H denotes non-C nucleotide) and non-symmetrical (CHH) cytosine sequences^3,4,6^. Precise DNA methylation regulation is crucial for numerous biological processes, including X-chromosome inactivation, transcriptional control, genomic imprinting, transposon silencing, and chromosomal stability maintenance^3,4^. Recent studies have revealed that DNA methylation dynamics is critical for fleshy fruit ripening^7–15^. During tomato (*Solanum lycopersicum*) fruit ripening, DEMETER-LIKE 2 (SlDML2)-mediated DNA demethylation occurs at more than 20,000 genomic regions, and this loss of DNA methylation indirectly facilitates access of the master regulator RIN to a subset of its target promoters^16^. Whether this ripening-induced demethylation also directly shapes transcription factor (TF)–DNA recognition remains untested.

TFs regulate downstream target genes by binding to specific cis-regulatory modules^17^. DNA methylation can alter the binding affinity between TFs and their target DNA sequences directly or indirectly^4,18–20^. Based on the affinity or sensitivity to methylated DNA, TFs generally fall into two categories: methylation-sensitive TFs and methylation-insensitive TFs, while the former can be further divided into methylation-inhibiting TFs and methylation-preferring TFs^18^. Most studies of TF methylation sensitivity have been reported in mammals^21–25^. A well-established example is CTCF, whose binding is highly sensitive to DNA methylation at imprinting control regions of the mouse *Igf2h* and *H19* genes^26,27^. Similarly, NRF1 exhibits strong in vivo sensitivity to DNA methylation; whereas global hypomethylation significantly expands its genomic binding profile, hypermethylation of its recognition motif impairs binding^28^. This methylation-dependent regulation of NRF1 is further illustrated in mouse spermatogenesis, where hypomethylation of promoter regions in germ cell-specific genes (e.g., *Asz1*) permits NRF1 binding and transcriptional activation, ensuring precise gene expression control during germ cell development^29^. Likewise, GATA-1 binding to an intronic regulatory element in the *c-Kit* gene is completely inhibited by methylation of a single cytosine within the CGATA motif, a mechanism essential for precise regulation of gene expression during hematopoietic differentiation^30^. In contrast, research on DNA methylation sensitivity among plant TFs remains limited. In vitro studies have revealed that two *Arabidopsis* TFs, AtMYB4 and AtWRKY40, exhibit methylation sensitivity, with 5-mC directly interfering with their DNA-binding activity^31,32^. Notably, a recent study on epigenetic regulation in rice cold adaptation demonstrated that low-temperature stress significantly reduces DNA methylation in the *OsACT1* promoter, enhancing OsDof1 binding and subsequently activating *OsACT1* expression, thereby improving cold tolerance^33^.

The SPL (SQUAMOSA PROMOTER BINDING PROTEIN (SBP)-LIKE) gene family encodes plant-specific TFs defined by a conserved SBP domain of about 76 amino acids, with a nuclear localization signal (NLS) and two zinc-binding sites^34–36^. SPL TFs orchestrate critical processes such as developmental phase transitions, organ morphogenesis, secondary metabolism, and stress responses^35,36^. In *Arabidopsis*, 16 *AtSPL* TFs have been identified, and their functions in root, leaf, and floral development; stage transitions; secondary metabolism; and stress responses are well characterized^35,37–40^. Moreover, *SPL* genes are pivotal regulators of agronomic traits in crops. For instance, *OsSPL14* (also known as *IPA1* or *WFP*) in rice is a well-characterized pleiotropic regulator that modulates blast resistance, tillering, panicle branching, grain yield, grain appearance, and stress responses^41–48^. *OsSPL8* (also known as *OsLG1*) regulates the development of ligule and auricle, as well as panicle and inflorescence architecture traits^49–52^. *OsSPL13* (also known as *GLW7*) controls grain length and weight by positively regulating lemma cell size^53^. In maize, three homologous *ZmSPL* genes—*ZmSPL8* (also known as *UB2*), *ZmSPL30* (also known as *UB3*), and *ZmSPL2* (also known as *TSH4*) —exhibit significant pleiotropy and functional redundancy in regulating inflorescence development, plant architecture, and grain traits^54,55^. *ZmSPL1* (also known as *TGA1*) negatively regulates glume development, ear prolificacy, and shank elongation, whereas *ZmSPL15* (also known as *ZmLG1*) acts as a positive regulator of critical traits, including leaf angle, tassel branch number, and tassel branch angle^56,57^. These findings highlight the pleiotropic functions and functional diversification of SPLs across plant species. However, whether and how SPL-family TFs recognize DNA methylation in the plant genome remains unexplored.

Tomato represents one of the most economically significant vegetable crops worldwide and serves as a valuable model system for exploring the mechanisms underlying fruit development^58,59^. In tomato, a total of 16 *SPL* genes have been identified, but the functions of most members remain to be fully elucidated^60–64^. Among these, *SlSPL-CNR* (*Colorless Non-Ripening*) is a well-studied member associated with flavonoid biosynthesis, wax synthesis, carotenoid biosynthesis, and cell wall metabolism during tomato fruit development^65–69^. The *Cnr* epiallele, caused by hypermethylation in the *SlSPL-CNR* promoter, leads to non-ripening fruits^70^. Despite its agricultural significance, whether DNA methylation directly influences SlSPL-CNR binding activity and how such regulation integrates with ripening-associated hypomethylation remains unclear.

In this study, we employed high-throughput DNA affinity purification sequencing (DAP-seq), amplification-based DAP-seq (ampDAP-seq), and whole-genome bisulfite sequencing (WGBS) to demonstrate that SlSPL-CNR exhibits sensitivity to DNA methylation in vitro. Further biochemical and structural analyses showed that 5-mC within the recognition motif sequence interferes with SlSPL-CNR binding via steric hindrance effects. Moreover, in vivo chromatin immunoprecipitation sequencing (ChIP-seq) analyses of SlSPL-CNR in green and breaker-stage fruits revealed that ripening-associated DNA demethylation can modulate SlSPL-CNR binding patterns. Notably, we identified a tomato flavor-related gene, *alcohol acyltransferase 1* (*SlAAT1*), as a downstream target of SlSPL-CNR. Hypomethylation of the *SlAAT1* promoter, mediated by SlDML2, was shown to be essential for SlSPL-CNR binding, underscoring the pivotal role of SlSPL-CNR’s methylation sensitivity in its biological function in tomato fruit development. Finally, we showed that certain SBP proteins in rice, maize, and tomato exhibit conserved DNA methylation sensitivity, suggesting that the interaction between SPL TFs and DNA methylation may influence agronomically important traits beyond fruit ripening. These findings enhance our understanding of the interplay between transcription factor function and DNA methylation.

## Results

### SlSPL-CNR prefers to bind hypomethylated genomic regions in vitro

**Fig. 1:**
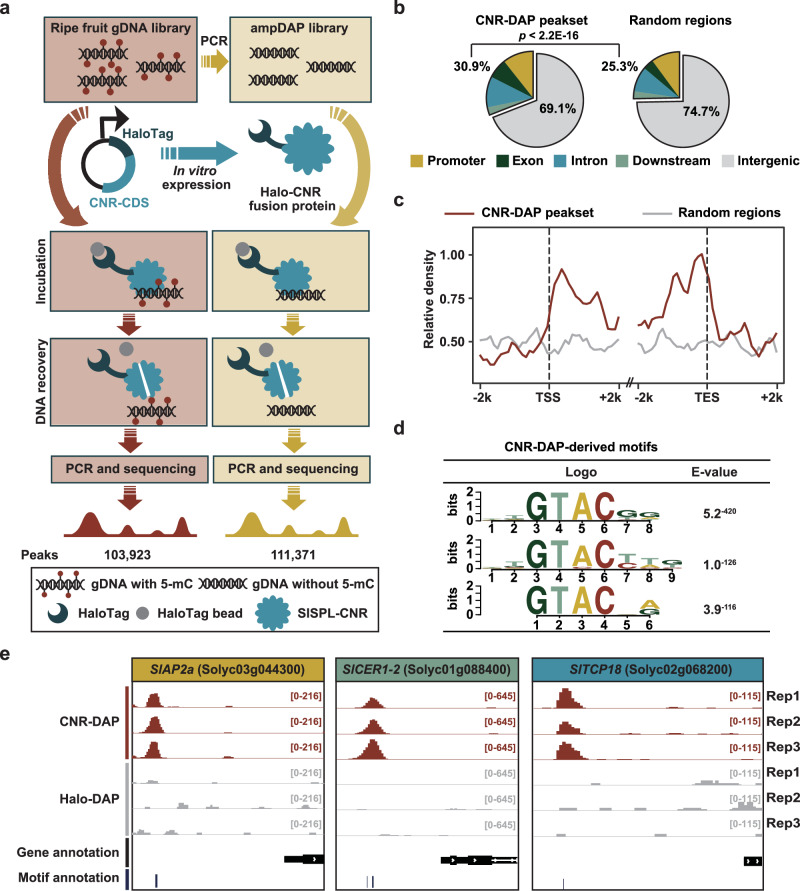
DAP-seq and ampDAP-seq profiling of SlSPL-CNR. **a** Schematic overview of DAP-seq and ampDAP-seq workflows for profiling SlSPL-CNR binding in wild-type ripe-stage fruits at 46 dpa. **b** Genomic distribution of CNR-DAP binding peakset (left) and random genomic regions (right) across various genomic features, including promoter (regions defined as spanning 2 kb upstream and 0.5 kb downstream of the transcription start site (TSS)), exons, introns, downstream (0–2 kb downstream of transcription end site (TES)), and intergenic regions. *P* value was calculated using a two-tailed two-proportion *z*-test (*P* < 2.2E-16). **c** Enrichment of CNR-DAP binding peaks across regions spanning ±2 kb around the TSS and TES of gene structures. **d** Top three de novo DNA motifs recovered from CNR-DAP peaks, ranked by *E* value. **e** Integrative Genomics Viewer (IGV) tracks illustrating reproducible CNR-DAP peaks at three established SlSPL-CNR target genes (*SlAP2a*, *SlCER1-2*, and *SlTCP18*). Halo-DAP serves as a negative control.

DAP-seq efficiently identifies TF binding sites in vitro while preserving native 5-mC, whereas ampDAP-seq, involving PCR amplification, removes nearly all cytosine methylation^71^. Integrative analysis of DAP-seq, ampDAP-seq, and DNA methylome data provides insight into TF binding at methylated loci^72^. To characterize the methylation sensitivity of SlSPL-CNR’s binding to genomic DNA, we first performed DAP-seq using genomic DNA (gDNA) libraries prepared from 46 days post-anthesis (dpa) ripe fruits (referred to as CNR-DAP) and ampDAP-seq using amplified libraries (referred to as CNR-ampDAP) (Fig. 1a). Concurrently, WGBS was conducted on wild-type fruits at 46 dpa to generate a comprehensive DNA methylation map (referred to as WT-Red) (Supplementary Data 1). CNR-DAP and CNR-ampDAP showed high reproducibility between replicates, detecting 103,923 and 111,371 binding peaks, respectively (Fig. 1a and Supplementary Fig. 1a, b). These peaks were significantly enriched in genic regions and around transcription start and end sites, compared to random region controls (Fig. 1b, c). De novo motif analysis identified three core motifs, GTACGG, GTACTT, and GTACNA (N denotes any nucleotide base), containing ‘GTAC’ as the most statistically enriched sequences in CNR-DAP, aligning with the binding characteristics typically associated with the SPL family (Fig. 1d)^34,73–76^. Previous studies reported that SlSPL-CNR can bind the promoters of *SlAP2a*, *SlCRE1-2*, and *SlTCP18*^65,77^, which were successfully captured by CNR-DAP (Fig. 1e). Therefore, the binding profiles generated under both conditions establish a solid foundation for analyzing how 5-mC influences SlSPL-CNR recruitment across the tomato genome.

**Fig. 2:**
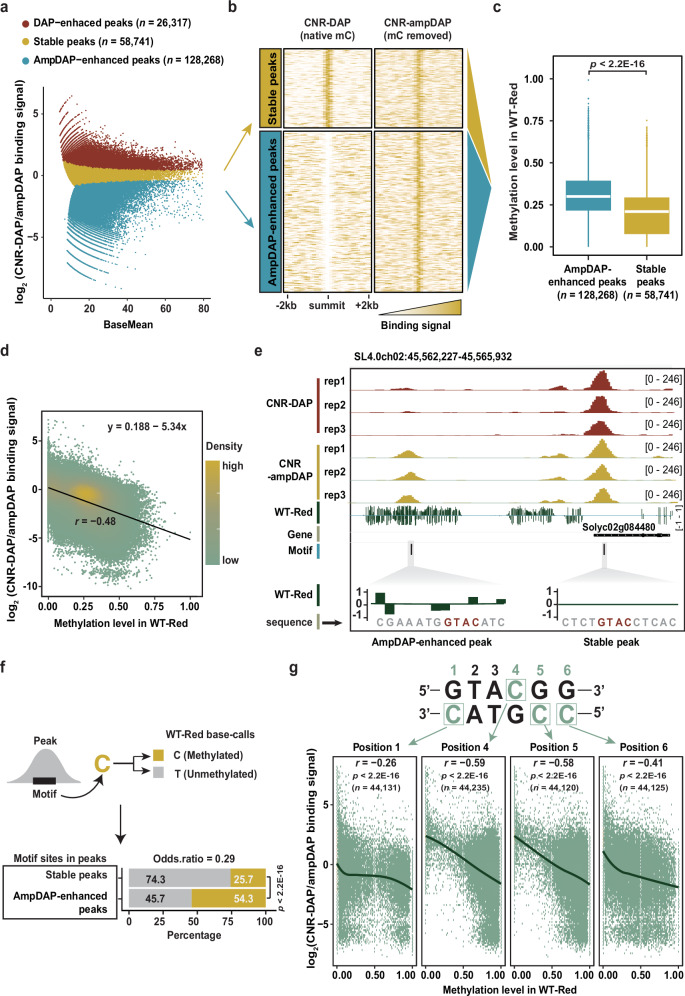
Cytosine methylation modulates SlSPL-CNR binding in vitro. **a** Scatter plot of log_2_(CNR-DAP/CNR-ampDAP) binding signal ratios versus basemean for 213,326 peak regions (±200 bp around the normalized peak summit), with peaks categorized as DAP-enhanced (red), stable (yellow), or ampDAP-enhanced (blue). **b** Heatmap depicting CNR-DAP and CNR-ampDAP binding signal enrichment at ampDAP-enhanced and stable peaks. **c** Boxplot showing significant divergence in DNA methylation levels between ampDAP-enhanced and stable peaks in WT-Red. Boxes show median values and the interquartile range of DNA methylation levels, and whiskers show the minimum and maximum observations within 1.5× IQR (interquartile range) of Q1 and Q3, with dots representing outliers beyond the whiskers. *P* value was calculated using a two-tailed Wilcoxon rank-sum test (*P* < 2.2E-16). The peak numbers for these two groups are shown. **d** Density scatter plot illustrating the relationship between log_2_(CNR-DAP/CNR-ampDAP) binding signal ratios and DNA methylation levels in WT-Red, with a Pearson correlation coefficient (*r* = −0.48) indicating a negative correlation. **e** IGV visualization of SlSPL-CNR binding and associated DNA methylation levels at selected loci in CNR-DAP and CNR-ampDAP datasets. **f** Bar graph showing the proportions of methylated versus unmethylated motifs in ampDAP**-**enhanced peaks and stable peaks, with ampDAP-enhanced peaks exhibiting significant enrichment of methylated motifs. *P* value was calculated using a two-tailed Fisher’s exact test (*P* < 2.2E-16). **g** Scatter plot depicting Pearson correlation coefficients (*r*) between log_2_(CNR-DAP/CNR-ampDAP) binding signal ratios and methylation probabilities at each cytosine position within GTACGG motif-matched sites (*n* = 46,408). *P* value was calculated using a Pearson correlation test. The numbers for each site are shown.

To evaluate the impact of DNA methylation on SlSPL-CNR binding, we performed an integrated analysis incorporating the CNR-DAP, CNR-ampDAP, and DNA methylome data (Supplementary Fig. 1c, d). Among a total of 213,326 peaks, we classified 26,317 as DAP-enhanced, 58,741 as stable, and 128,268 as ampDAP-enhanced based on relative signal intensities (Fig. 2a, b and Supplementary Data 2). The large number of ampDAP-enhanced peaks indicated a large-scale gain of SlSPL-CNR binding in CNR-ampDAP compared to CNR-DAP. Considering that ampDAP libraries are depleted of mC modifications, we hypothesized that the gain of SlSPL-CNR binding may result from the loss of DNA methylation in CNR-ampDAP. Consistently, ampDAP-enhanced peaks exhibited significantly higher methylation levels than stable peaks (Fig. 2c). We further quantified the CNR-DAP to CNR-ampDAP signal ratio across all binding regions as a function of the methylation level, as previously reported^31,78^. This analysis demonstrated a notable negative correlation (*r* = −0.48), where the DAP to ampDAP binding signal ratio decreased as DNA methylation levels increased (Fig. 2d). These results demonstrate that ampDAP-enhanced peaks originate from hypermethylated regions that become accessible upon 5-mC depletion, indicating a preference of SlSPL-CNR binding to unmethylated DNA. The examination of representative loci confirmed this pattern: low methylation regions exhibited TF binding in both assays, whereas highly methylated regions showed binding exclusively in CNR-ampDAP (Fig. 2e and Supplementary Fig. 2a).

Prior research has highlighted the potential influence of cytosine within DNA-binding motifs on methylation sensitivity^23,24,72^. Considering that the de novo annotated SlSPL-CNR core motif ‘GTACGG’ containes several cytosines (Fig. 1d), we investigated whether motif methylation modulated binding. We observed that the ampDAP-enhanced peaks exhibited significant enrichment of methylated motifs compared to stable peaks (Fig. 2f). For the top annotated motif sequence ‘GTACGG’, we assessed methylation effects at each cytosine position (1, 4, 5, and 6) by scanning the tomato genome for motif-matched sites (Supplementary Fig. 2b) and correlating position-specific methylation with the CNR-DAP to CNR-ampDAP signal ratio. All four positions showed reduced binding upon 5-mC incorporation, with the most pronounced inhibition at position 4 on the sense strand (5-mC_4_; *r* = −0.59) (Fig. 2g), identifying this residue as the key determinant of methylation-sensitive SlSPL-CNR binding.

### Cytosine methylation in the GTAC-box reduces the DNA-binding affinity of SlSPL-CNR

**Fig. 3:**
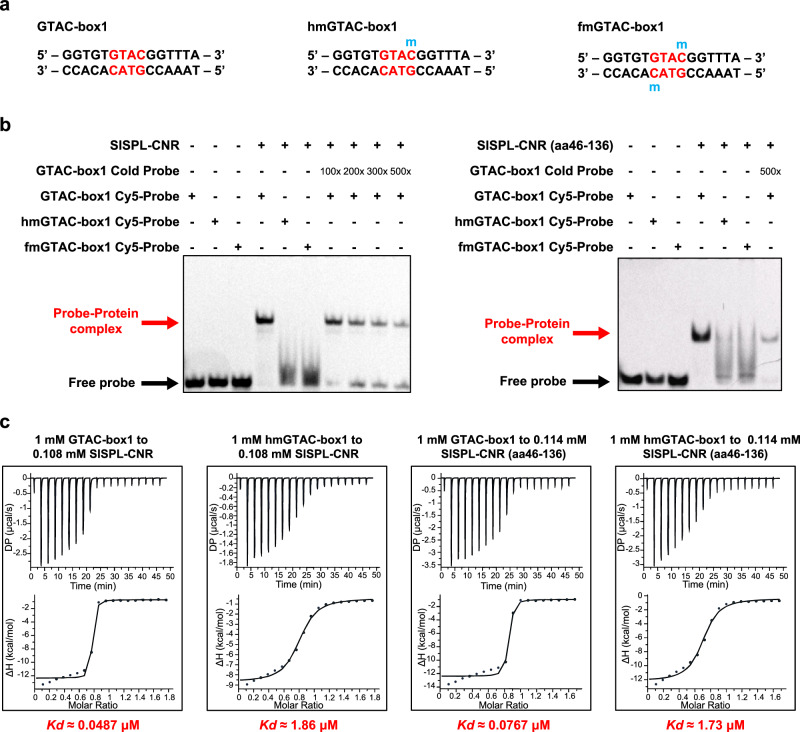
DNA-binding specificity of SlSPL-CNR to differentially methylated GTAC motifs. **a** EMSA and ITC probes harboring the GTAC motif in unmethylated (GTAC-box1), hemi-methylated (hmGTAC-box1; 5-mC on sense strand), or fully methylated (fmGTAC-box1; 5-mC on both strands) states; GTAC-box1 (SL4.0ch02: 43,707,574–43,707,613) was identified as a SlSPL-CNR target by DAP-seq. **b** Competition EMSA of SlSPL-CNR binding to differentially methylated GTAC probes. Full-length SlSPL-CNR and the SBP-domain fragment (aa 46–136) were incubated with 1 μM Cy5-labeled GTAC-box1, hmGTAC-box1, or fmGTAC-box1 probes. Binding specificity was challenged by the addition of unlabeled GTAC-box1 competitor (100-, 200-, 300-, or 500-fold excess). Experiments were repeated independently three times with similar results. **c** ITC analysis of SlSPL-CNR protein binding to unmethylated and hemi-methylated GTAC-box1. The full-length SlSPL-CNR and the SBP-domain fragment (aa 46–136) were used.

To further investigate whether and to what extent 5-mC inhibits the direct binding of SlSPL-CNR to the GTAC-box core motif, we conducted electrophoretic mobility shift assays (EMSAs) using 15-bp DNA probes (GTAC-box1), which encompass unmethylated, hemi-methylated (5-mC on the sense strand only), and fully methylated (5-mCs on both strands) ‘GTAC’ motif sequence (Fig. 3a). The EMSA results demonstrated that both full-length SlSPL-CNR and its SBP domain (amino acids 46–136, aa 46–136) bound effectively to the unmethylated DNA probes but failed to bind to probes with hemi-methylated or fully methylated cytosines at the GTAC-box (Fig. 3b). These results demonstrate that 5-mC in GTAC-box can decrease the DNA-binding affinity of SlSPL-CNR.

To quantitatively assess the binding sensitivity of SlSPL-CNR to methylation at the motif sequence, we carried out isothermal titration calorimetry (ITC). We measured the binding affinity of the full-length SlSPL-CNR protein and its SBP domain (aa 46–136) for GTAC-box probes identical to those used in the EMSA. Both SlSPL-CNR and its SBP domain exhibited the strongest binding affinity to the unmethylated DNA probe (0.0487 and 0.0767 μM, respectively), whereas hemi-methylated DNA significantly reduced their binding affinities (1.86 and 1.73 μM, respectively) (Fig. 3c and Supplementary Data 3). In addition, ITC analysis of another GTAC-containing probe (GTAC-box2) further confirmed that the presence of 5-mC within the ‘GTAC’ motif reduced the binding affinity of both full-length SlSPL-CNR and its SBP domain (Supplementary Fig. 3a, b). In summary, the EMSA and ITC results collectively indicate that DNA methylation within the GTAC motif sequence significantly diminishes the binding affinity of SlSPL-CNR and its SBP domain to target DNA sequences.

### Crystal structures of SlSPL-CNR SBP domain in complex with unmethylated DNA

**Fig. 4:**
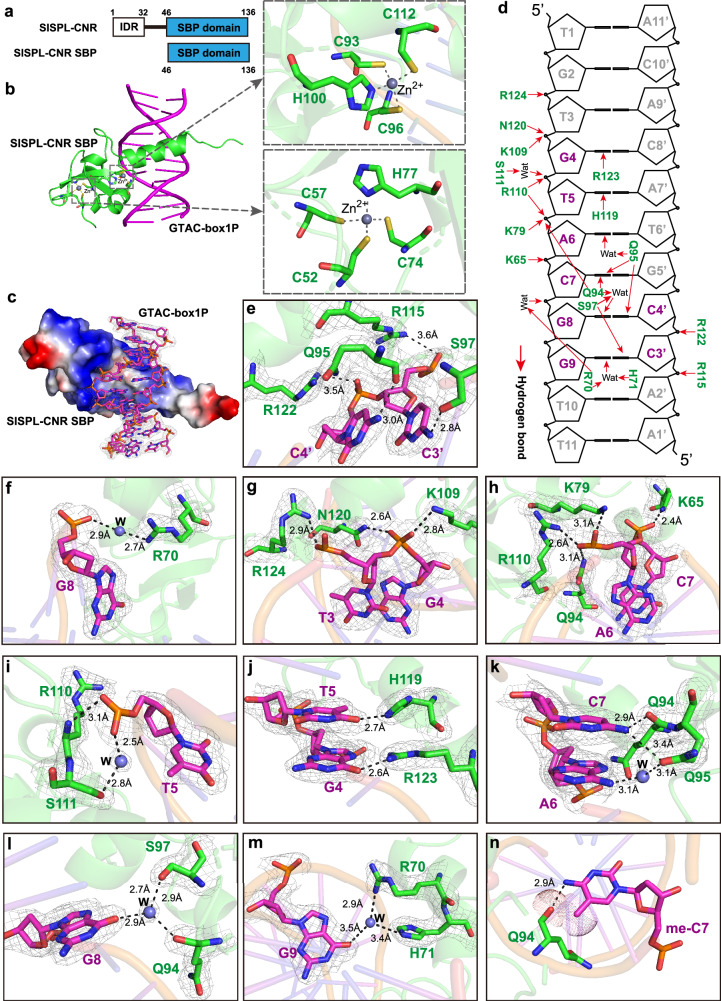
Crystal structure of the SlSPL-CNR SBP–DNA complex. **a** Domain architecture of SlSPL-CNR (upper panel) and the construct used for structural studies (lower panel). **b** Overall structure of the SlSPL-CNR SBP–DNA complex with GTAC-box1P (a partial sequence of GTAC-box1) shown in magenta and the CNR SBP domain in green. The two zinc finger motifs are zoomed in, highlighting the zinc coordination residues in sticks. **c** Electrostatic surface view of CNR SBP domain, showing that the GTAC-box1P is captured by a positively charged DNA-binding interface. GTAC-box1P shows as a stick model with electron density maps. The mesh model is 2FoFc, and the sigma level is 1.0. **d** Schematic representation of the interactions between SlSPL-CNR SBP domain and GTAC-box1P. Hydrogen bonds are indicated by red arrows. **e**–**i** Details of the recognition of the DNA phosphate backbone by SlSPL-CNR SBP domain. The interacting residues and hydrogen bonds are highlighted by sticks and dashed silver lines, respectively. **j**–**m** Details of the recognition of DNA bases by SlSPL-CNR SBP domain. W represents the water. **n** Modeling of C7 as a methylated cytosine base reveal that the introduced methyl group would cause a steric clash with Gln94, thereby providing a structural explanation for the preference of the SlSPL-CNR protein for unmethylated DNA.

To investigate the molecular basis of the recognition of unmethylated DNA by SlSPL-CNR, we carried out structural studies with the SlSPL-CNR SBP domain, which is responsible for the DNA-binding function of SlSPL-CNR (Fig. 4a). The crystal structure of the CNR SBP domain in complex with an unmethylated GTAC-box1P was determined by the molecular replacement method and refined to 1.7 Å resolution (Fig. 4b and Supplementary Data 4). Overall, there is only one protein–DNA complex in the asymmetric unit. The plant-specific SBP domain is composed of two closely linked zinc fingers, and the DNA adopts a classic B-form conformation (Fig. 4b). A long α-helix and a loop insert into the major groove of the DNA, while several positively charged residues form a continuous surface to accommodate the DNA backbone (Fig. 4b, c). The complex formation buries about 940 Å^2^ solvent-accessible surface area on both the SlSPL-CNR SBP domain and GTAC-box1P (Fig. 4c). The polar residues Lys65, Arg70, Lys79, Gln94, Lys109, Arg110, Ser111, Arg115, Asn120, Arg122, and Arg124 form extensive hydrogen bonding and salt bridge interactions with the DNA backbone, strengthening the overall interaction between SlSPL-CNR SBP and DNA (Fig. 4d–i). Consistent with the specific recognition of the GTACGG *cis*-element by SlSPL-CNR, all these bases, and/or their paired bases, have direct interactions with the SlSPL-CNR SBP domain. In detail, His119 and Arg123 form hydrogen bonding interactions with the carbonyl groups of G4 and T5 (Fig. 4j). Gln95 forms hydrogen bonds with the amino groups of A6 and C7 (Fig. 4k and Supplementary Fig. 4). Gln94 not only forms a hydrogen bond with C7 but also forms a water-mediated hydrogen bond with G8 in cooperation with Ser97 (Fig. 4k, l and Supplementary Fig. 4). Arg70 and His71 interact with G9 via a water-mediated hydrogen bond (Fig. 4m). In addition, Gln95 and Ser97 also form hydrogen bonds with the amino groups of C4’ and C3’ of the reverse strand (Fig. 4e). Overall, all the bases of the *cis*-element (marked by magenta in Fig. 4d) are strictly recognized by the hydrogen bonding interaction network, highlighting the sequence-specific recognition. Further analysis by EMSA revealed that site-directed mutagenesis substituting Gln with Ala at Gln94 (Q94A) or Gln95 (Q95A) in SlSPL-CNR weakened its binding to target GTAC-box1 (Supplementary Fig. 5). It is noteworthy that, in comparison to single-point mutations, the double mutation (Q94/95A) at positions 94 and 95 exhibits a significantly stronger inhibitory effect on DNA-binding affinity. This reveals that Q94/95 are two key amino acid residues in determining the binding affinity to the GTACGG *cis*-element (Supplementary Fig. 5). In the GTACGG *cis*-element, the middle CG site can be methylated, while SlSPL-CNR only recognizes the unmethylated form but not the methylated form of this *cis*-element. We modeled a methylated cytosine at the C7 position of GTAC-box1P using our unmethylated DNA complex structure as a reference. The methyl-group of the putative mC7 has severe steric conflict with the main chain carbonyl group of Gln94 (Fig. 4n). Consequently, both the C7 base and the Q94 main chain cannot move or rotate, and the methylation of C7 will undoubtedly introduce steric conflict between the methyl group and the Q94 main-chain carbonyl as shown in Fig. 4n, explaining the unmethylated DNA preference for the CNR SBP domain. Thereby, the SlSPL-CNR SBP domain prevents the binding to the methylated DNA, supporting a methylation status-sensitive binding mode. Considering that C4’ of the complementary strand also forms a base-specific interaction with SlSPL-CNR (Fig. 4d), we further investigated the impact of C4’ methylation on SlSPL-CNR binding. EMSA results demonstrate that DNA probes with C4’ methylation did not show a significant inhibitory effect on SlSPL-CNR binding (Supplementary Fig. 6).

### Comparative analysis of in vivo and in vitro SlSPL-CNR binding profiles

**Fig. 5:**
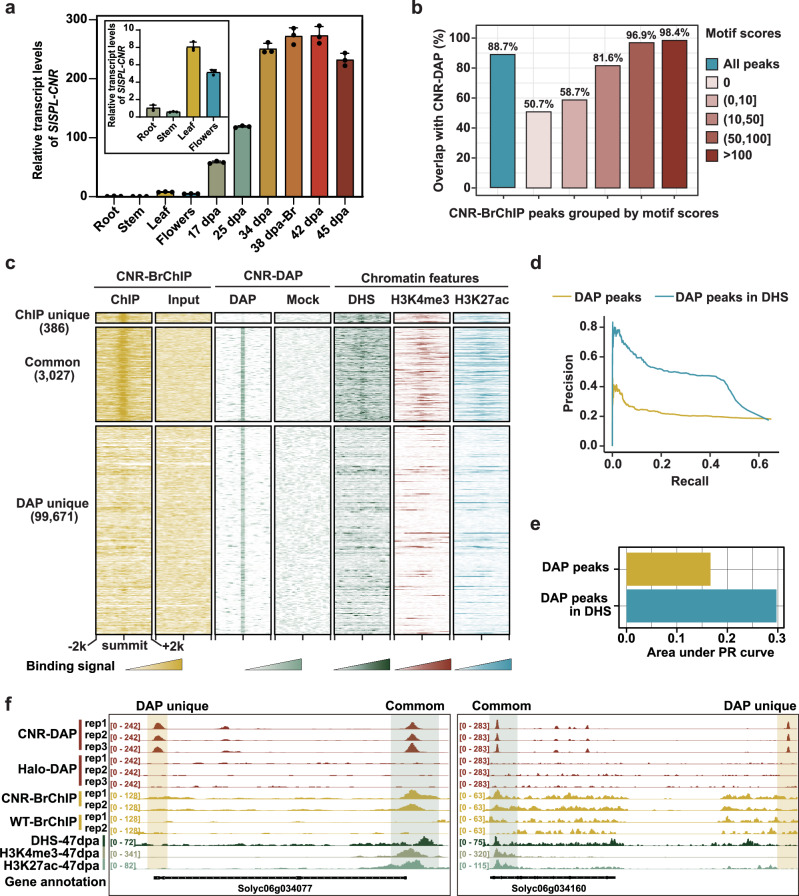
Comparative analysis of SlSPL-CNR-DAP-seq and ChIP-seq peaks. **a** Relative transcript levels of *SlSPL-CNR* in various tomato tissues (root, stem, leaf, flower) and different developing-stage fruits (17 dpa, 25 dpa, 34 dpa, 38 dpa, 42 dpa, and 45 dpa), as determined by RT-qPCR. Data are presented as means ± SD from three biological replicates**. b** The percentage overlap between CNR-BrChIP peaks and CNR-DAP peaks increases with higher motif scores. **c** Heatmaps show binding signal enrichment of CNR-BrChIP, CNR-DAP, chromatin accessibility (DHS), and histone modifications (H3K4me3, H3K27ac) at ChIP unique, ChIP-DAP common (common), and DAP unique peak regions (±2 kb around the peak summit). **d** Precision–recall curves indicate that CNR-DAP peaks located within DHS regions predict CNR-BrChIP peaks more accurately than CNR-DAP peaks alone. **e** The area under the precision–recall curve (AUPRC) demonstrates that CNR-BrChIP peaks are better predicted by CNR-DAP peaks within DHS regions compared to CNR-DAP peaks alone. **f** IGV snapshots of representative genomic regions show that common peaks are associated with increased DHS, H3K4me3, and H3K27ac signals, whereas DAP unique peaks lack these chromatin features.

Recent studies have demonstrated that *SlSPL-CNR* plays a crucial role in flavonoid biosynthesis, wax synthesis, carotenoid accumulation, and cell wall metabolism in tomato fruits^65–69^. RT-qPCR results revealed that *SlSPL-CNR* shows higher expression levels in fruit tissues compared to other tissues, including roots, stems, leaves, and flowers (Fig. 5a). To compare in vivo and in vitro DNA-binding features of SlSPL-CNR, we conducted ChIP-seq with fruits expressing native promoter-driven *gCNR-3xFLAG* at 38 dpa (breaker (Br) stage) (designated CNR-BrChIP) (Supplementary Fig. 7a). In CNR-BrChIP, a total of 3413 peaks were identified, and 3027 of them overlapped with CNR-DAP peaks (Fig. 5b, c), suggesting that CNR-DAP captured nearly 90% of CNR-BrChIP peaks. Based on CNR-BrChIP, the core sequence determined by the motif analysis was ‘GTACGG’, consistent with CNR-DAP-derived representative motifs (Supplementary Fig. 7b and Fig. 1d). We ranked CNR-BrChIP peaks according to motif scores and found that the overlap with CNR-DAP peaks increased progressively with higher motif scores, indicating that DAP-seq preferentially captured in vivo binding sites associated with high-scoring motifs (Fig. 5b).

We further evaluated how 5-mC at different positions of the GTACGG core motif impacted SlSPL-CNR binding in CNR-BrChIP by integrating the DNA methylome of the fruit at the breaker stage in wild-type tomato (designated WT-Br) from previously published WGBS data^16^. Consistent with results in CNR-DAP (Fig. 2f), cytosines at different positions within the GTACGG motif exerted varying inhibitory effects on SlSPL-CNR binding in CNR-BrChIP, with the most pronounced inhibition (*r* = −0.46) observed at the fourth position of cytosine (Supplementary Fig. 7c), suggesting that SlSPL-CNR has similar DNA methylation-sensitive binding features in vivo and in vitro.

Although many SlSPL-CNR binding features are similar in vivo and in vitro, we identified 99,671 peaks that were only detected by CNR-DAP (DAP unique) (Fig. 5c). By comparing the genomic distributions of DAP unique and ChIP-DAP common peaks, we found that most common peaks were located in genic regions, whereas DAP unique peaks were enriched in intergenic regions (Supplementary Fig. 8a), indicating that the in vitro assay detects many intergenic peaks that do not exist in vivo. To dissect whether chromatin accessibility and histone modifications affect CNR in vivo binding, we integrated publicly available DNase I hypersensitivity site (DHS) and active histone marks (H3K4me3 and H3K27ac) ChIP-seq data^79^. DAP unique peaks exhibited reduced DHS signals and lower enrichment of H3K4me3 and H3K27ac relative to ChIP-DAP common peaks (Fig. 5c), suggesting that chromatin accessibility restricts in vivo binding. To investigate how well CNR-DAP plus DHS approximated CNR-BrChIP, we ranked two peak sets: all CNR-DAP peaks and the subset that fell within DHS regions. Using precision–recall (PR) curves as previously reported^72^, we benchmarked how well CNR-DAP alone or CNR-DAP restricted to DHS regions recovered the gold-standard CNR-BrChIP peaks. Integrating DHS lifted the area under the PR curve well above DAP-only, confirming that chromatin accessibility filtering markedly sharpened in vivo binding prediction (Fig. 5d, e). As shown in the IGV screenshots, CNR-BrChIP and CNR-DAP shared peaks, but not DAP unique peaks, are associated with enrichment of DHS and active H3K4me3/H3K27ac histone marks (Fig. 5f and Supplementary Fig. 8b). These findings indicate that integrating DAP-seq data with epigenetic information may provide better prediction of the in vivo binding patterns of specific TFs.

### Ripening-induced DNA hypomethylation partly reshapes SlSPL-CNR binding profiles in vivo

**Fig. 6:**
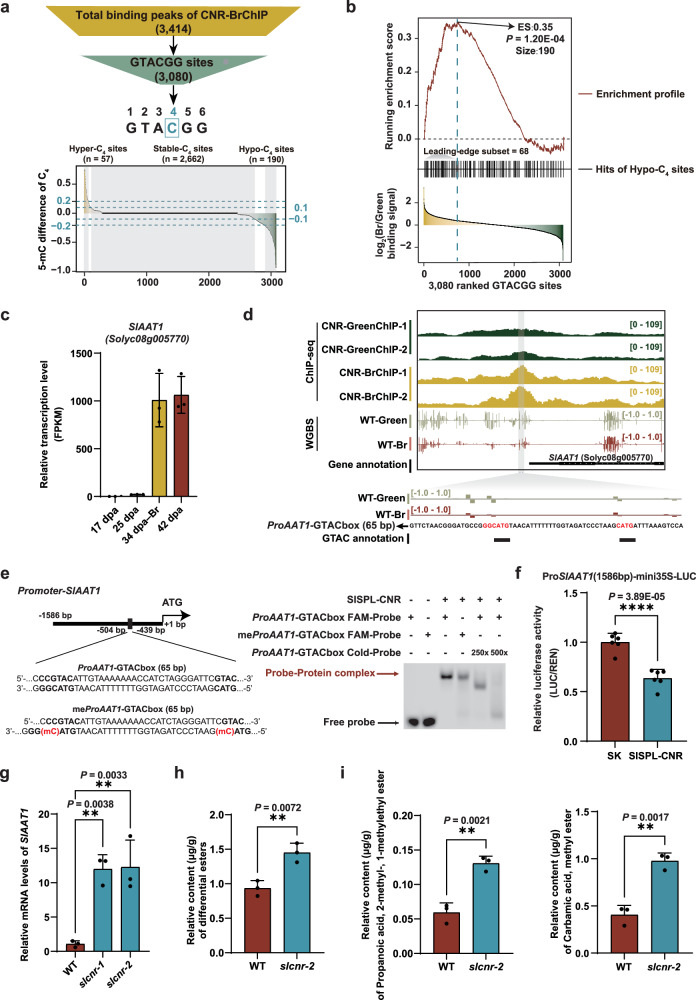
Ripening-associated DNA hypomethylation enhances SlSPL-CNR–*SlAAT1* binding to modulate flavor esters. **a** Classification of C_4_-methylation dynamics within SlSPL-CNR–bound GTACGG motifs. From 3414 high-confidence CNR-BrChIP peaks, 3080 5′-GTACGG-3′ sites were identified. Sites were classified by methylation changes (between the breaker (Br) and green stages) at C₄ as stable-C₄ (|Δmeth | ≤ 0.10, *n* = 2662), hyper-C₄ (Δmeth ≥ 0.20, *n* = 57), or hypo-C₄ (Δmeth ≤ –0.20, *n* = 190). **b** Gene set enrichment analysis (GSEA) of 3080 GTACGG sites located within CNR-BrChIP peaks, ranked by log₂ fold change in CNR binding (Br vs. green stage). The enrichment curve yields an enrichment score (ES) of 0.35, suggesting that Hypo-C₄ sites (*n* = 190) are significantly enriched among regions showing stronger CNR binding at the Br stage. The leading-edge subset (size = 68; blue dashed line) represents the primary contributors to this enrichment. *P* value was calculated using a two-sided permutation test with 10,000 permutations (*P* = 1.20E-04). **c** *SlAAT1* transcript abundance (FPKM) in fruits at 17, 25, 34 (Br stage), and 42 dpa. Data are presented as mean ± SD from three biological replicates. **d** IGV tracks depicting SlSPL-CNR ChIP-seq binding and DNA methylation at the *SlAAT1* promoter, with enlarged views of GTAC motifs and their corresponding methylation levels shown below. **e** EMSA analysis of SlSPL-CNR binding to the *SlAAT1* promoter using 65-bp unmethylated (*ProAAT1*-GTAC) or hemi-methylated (me*ProAAT1*-GTAC; two 5-mCs at antisense strand) FAM-labeled probes. Full-length SlSPL-CNR was incubated with 1 µM probe, and binding specificity was confirmed by competition assays with 250-fold or 500-fold excess of unlabeled competitor probe. “+“ and “–“ indicate the presence or absence of respective components. Experiments were repeated independently three times with similar results. **f** Dual-luciferase assay demonstrating SlSPL-CNR repression of *SlAAT1* promoter activity. Pro*AAT1* (1586 bp) represents a 1586-bp fragment of the *SlAAT1* promoter. Data are presented as mean ± SD from six biological replicates. Asterisks indicate statistical significance by a two-tailed Student’s *t* test (****P < 0.0001). **g** RT-qPCR analysis of *SlAAT1* relative transcript levels in WT and *slcnr-1/2* mutant fruits at the Br stage. Data are presented as mean ± SD from three biological replicates. Asterisks indicate statistical significance by one-way ANOVA with Dunnett’s multiple comparison test (**P < 0.01). **h** The summed relative content of five differential ester metabolites in WT and *slcnr-2* fruits. Data are presented as mean ± SD from three biological replicates. Asterisks indicate statistical significance by a two-tailed Student’s *t* test (**P < 0.01). **i** Relative content of two ester metabolites (carbamic acid, methyl ester, and propanoic acid, 2-methyl-, 1-methylethyl ester) in *slcnr-2* mutants compared to WT fruits at the Br stage. Data are presented as mean ± SD from three biological replicates. Asterisks indicate statistical significance by a two-tailed Student’s *t* test (**P < 0.01). Source data are provided in a Source Data file.

We observed that *SlSPL-CNR* maintains high expression levels in fruits across the green (34 dpa), breaker (38 dpa), and ripe stages (42 dpa), indicating that, unlike some known ripening-related TFs, such as *Ripening Inhibitor* (*RIN*) and *FRUITFULL 1* (*FUL1*), *SlSPL-CNR* is involved in ripening-related transcriptional regulation independent of its own ripening-induced upregulation (Fig. 5a)^16^. Given that tomato fruit ripening entails global DNA demethylation^8,9^, we investigated whether ripening-induced DNA hypomethylation modulates SlSPL-CNR binding. We performed ChIP-seq in *pCNR::gCNR-3xFLAG* fruits at the green fruit stage (30 dpa; referred to as CNR-GreenChIP), and compared CNR-GreenChIP with CNR-BrChIP data. We hypothesized that the ripening-induced loss of DNA methylation at recognition motifs might lead to increased SlSPL-CNR binding in CNR-BrChIP relative to CNR-GreenChIP. To test this hypothesis, we focused on the methylation change (comparing WT-Br with WT-Green) and SlSPL-CNR binding change at 3080 ‘GTACGG’ *cis*-element binding sites within 3414 CNR-BrChIP binding peaks (Fig. 6a and Supplementary Data 5). We identified a total of 190 ‘GTACGG’ binding sites that were hypomethylated at the fourth position (hypo-C_4_ sites) during ripening (Fig. 6a). To further dissect the association between DNA methylation dynamics and SlSPL-CNR binding changes, we performed a Gene Set Enrichment Analysis (GSEA) (Fig. 6b and Supplementary Data 6). We ranked 3080 GTACGG *cis*-element binding sites in descending order based on the binding signal change between CNR-BrChIP and CNR-GreenChIP to assess the enrichment of hypo-C_4_ sites. A positive enrichment score (ES: 0.35) indicated that hypo-C_4_ sites were predominantly enriched in regions with enhanced binding signals, further showing that the dynamic hypomethylation of C_4_ within the GTACGG *cis*-element was strongly associated with the increase in vivo binding affinity of SlSPL-CNR from green to breaker stage (Fig. 6b). Representative loci are shown in Supplementary Fig. 9a, b. These results demonstrate that DNA hypomethylation at core motifs is associated with enhanced binding affinity of SlSPL-CNR during fruit ripening.

In the aforementioned GSEA analysis, the leading-edge subset driving the peak ES was identified; this subset represents recognition motif sites where SlSPL-CNR exhibits enhanced binding in response to fruit ripening-associated dynamic hypomethylation (Fig. 6b and Supplementary Data 6). KEGG analysis of genes linked to these sites revealed a relatively high enrichment in terms such as “metabolism” and “enzymes with EC numbers” (Supplementary Data 7), notably including the ripening-related gene *alcohol acyltransferase 1* (*SlAAT1*)—the primary alcohol acyltransferase responsible for ester volatile organic compound (VOC) biosynthesis during tomato fruit ripening (Fig. 6c, Supplementary Fig. 10, and Supplementary Data 7)^80–82^. As shown in the IGV screenshots, enhanced SlSPL-CNR binding at the promoter of *SlAAT1* correlates with DNA hypomethylation at two GTAC motif sites from the green stage to the Br stage (Fig. 6d). To determine whether SlSPL-CNR directly binds and is sensitive to 5-mC of the *SlAAT1* promoter, we analyzed the binding affinity between SlSPL-CNR and the 65-bp DNA probes from the *SlAAT1* promoter with or without 5-mCs. The EMSA assay showed that SlSPL-CNR could directly bind to the *SlAAT1* promoter in the absence of 5-mC, but exhibited reduced binding affinity toward hemi-methylated probes (Fig. 6e), demonstrating that methylation of recognition motif sequences restricted the binding of SlSPL-CNR to the *SlAAT1* promoter. Given that the inhibitory effect of 5-mC in Fig. 6e was less pronounced than the complete abolition observed with the short probe in Fig. 3a, b, and considering that the 65-bp *ProSlAAT1* probe contains two distinct GTAC motifs (GTACGG and GTACGA), we truncated it into two 20-bp fragments (PAAT1_GTAC-box1 and PAAT1_GTAC-box2) to assess motif-specific sensitivity (Supplementary Fig. 11). EMSA revealed that hemi-methylation completely blocked binding to the GTACGG-containing probe (consistent with Fig. 3b), whereas it only partially reduced binding to the GTACGA-containing probe, indicating that the differential methylation sensitivity of these two motifs accounts for the weaker inhibition observed in Fig. 6e.

Gln94 and Gln95 have been identified as critical residues governing the DNA-binding activity of SlSPL-CNR (Supplementary Figs.4a, b and  5). To determine whether these residues are equally essential for GTAC recognition motifs within the *SlAAT1* promoter, we performed EMSA assays using Q94A or/and Q95A mutant forms of the SlSPL-CNR (aa 46–136) (Supplementary Fig. 12a). EMSA results revealed that the Q94A mutation markedly impaired binding to both PAAT1_GTAC-box1 and PAAT1_GTAC-box2 probes, whereas the Q95A mutation caused a moderate reduction in binding affinity—indicating that Q94 is the primary determinant of GTAC motif recognition (Supplementary Fig. 12a). Consistent with this, further dual-luciferase reporter assays demonstrated that the Q94A mutant exhibited significantly attenuated repression of *SlAAT1* promoter-driven luciferase expression relative to the SBP domain, thereby establishing Q94 as a functionally indispensable residue for SlSPL-CNR–mediated transcriptional regulation of *SlAAT1* (Supplementary Fig. 12b).

### DNA hypomethylation-mediated SlSPL-CNR binding to *SlAAT1* regulates flavor esters

To better understand the regulatory relationship between SlSPL-CNR and *SlAAT1*, we performed dual-luciferase assays and observed that the transcriptional regulation of SlSPL-CNR on the *SlAAT1* promoter suppressed luciferase activity, indicating that SlSPL-CNR exerts a negative regulatory effect on *SlAAT1* (Fig. 6f). In addition, we employed CRISPR/Cas9 technology to generate two *SlSPL-CNR* knockout lines, i.e., *slcnr-1* and *slcnr-2*, in the *Ailsa Craig* (AC) genetic background (Supplementary Fig. 13a, b). We analyzed the *SlAAT1* transcript levels in WT, *slcnr-1*, and *slcnr-2* fruits at the Br stage using RT-qPCR. Our results demonstrated that *SlAAT1* expression is significantly higher in the *SlSPL-CNR* knockout lines compared to WT, further supporting the negative regulatory role of SlSPL-CNR on *SlAAT1* (Fig. 6g). This finding is consistent with previous studies reporting that fruit quality-related genes (such as *SlCER1-2*, *SlCER6*, *SlLACS2*, and *SlGDSL48*) are directly bound and negatively regulated by SlSPL-CNR, thereby highlighting its inhibitory regulatory role in fruit development^65,68^. It is worth noting that *SlAAT1* shows a significant increase throughout tomato fruit ripening (Fig. 6c)^80^ Previous studies established that the ripening regulator NOR directly binds and activates the *SlAAT1* promoter to promote ester volatile biosynthesis^82^. Therefore, we propose that SlSPL-CNR may serve as a “regulatory checkpoint”, suppressing the overexpression of *SlAAT1* via a negative feedback loop.

*SlAAT1* encodes an alcohol acyltransferase that catalyzes esterification of alcohols with acyl-CoA donors, thereby controlling volatile ester accumulation^80,81^. To precisely elucidate how SlSPL-CNR modulates fruit volatile metabolism, we analyzed the volatile metabolomes of *slcnr-2* mutants and WT tomato fruits at the Br stage using an integrated HS-SPME/GC-MS approach. A total of 819 VOCs were identified in both *slcnr-2* and WT, and these were classified into 15 clusters (Supplementary Fig. 14a and Supplementary Data 8). PCA analysis demonstrated clear separation between *slcnr-2* mutants and WT, suggesting substantial changes in the aroma metabolic profile caused by the *SlSPL-CNR* (Supplementary Fig. 14b). A total of 80 differentially accumulated VOCs were detected between WT and *slcnr-2* mutants, among which five were ester metabolites (Supplementary Fig. 14c, d and Supplementary Data 9). Analysis of these ester metabolites revealed that the overall ester content in the *slcnr-2* was significantly higher than that in WT (Fig. 6h). Among the five ester metabolites, four were upregulated, and two representative differentially esterified metabolites (carbamic acid, methyl ester and propanoic acid, 2-methyl-, 1-methylethyl ester) were significantly upregulated in *slcnr-2* (Fig. 6i). This result is consistent with the accumulation profile of ester metabolites associated with *SlAAT1* overexpression (Fig. 6h)^80,81^.

Genome-wide demethylation during ripening is known to be mediated by the DNA demethylase DEMETER-LIKE 2 (SlDML2), which is essential for tomato fruit ripening^9,83^. According to previous data^16^, *SlDML2* exhibits a ripening-induced expression pattern, showing peak expression at the breaker stage (Supplementary Fig. 15a). Interestingly, the DNA methylation of *SlAAT1* promoter was decreased in WT fruit but not in *dml2* mutant from green to breaker stages (Supplementary Fig. 15b). Moreover, ChIP-qPCR assays confirmed direct binding of SlDML2 to the *SlAAT1* promoter (Supplementary Fig. 15c). To rule out the possibility that SlSPL-CNR–mediated transcriptional repression indirectly alters the DNA methylation landscape of the *SlAAT1* promoter, we performed a comparative analysis of WGBS data from *slcnr* mutant and WT fruits at the breaker stage (Supplementary Fig. 16). No significant differences in DNA methylation levels were observed in the promoter regions of *SlAAT1* or *SlDML2* in *slcnr* relative to WT, indicating that loss of SlSPL-CNR does not trigger secondary methylation reprogramming at these loci (Supplementary Fig. 16). To assess potential functional synergy between SlSPL-CNR and SlDML2 in regulating *SlAAT1* expression, we first predicted their structural interaction using AlphaFold3 and subsequently tested for direct physical association via yeast two-hybrid (Y2H) assays; neither approach yielded detectable interaction signals, supporting the conclusion that SlSPL-CNR and SlDML2 do not engage in a stable, direct protein–protein interaction (Supplementary Fig. 17a, b). Together, these results demonstrate that SlDML2 is required for the demethylation of the *SlAAT1* promoter region during fruit ripening, and this DNA hypomethylation at the recognition motif enhances SlSPL-CNR binding to the *SlAAT1* promoter.

Collectively, these findings elucidate a negative feedback mechanism wherein ripening-induced, SlDML2-mediated demethylation of the *SlAAT1* promoter enhances SlSPL-CNR binding, thereby repressing *SlAAT1* transcription to maintain ester homeostasis during fruit development. While SlSPL-CNR binds the *SlAAT1* promoter to suppress its expression, the overall upregulation of *SlAAT1* during ripening is consistent with established activation by NOR^82^. This suggests that SlSPL-CNR functions not to abolish *SlAAT1* expression, but to attenuate its overexpression, serving as a developmental checkpoint that prevents premature ester accumulation while permitting ripening-induced accumulation (Supplementary Fig. 18).

### Conserved DNA methylation sensitivity in SBP-domain TF families

**Fig. 7:**
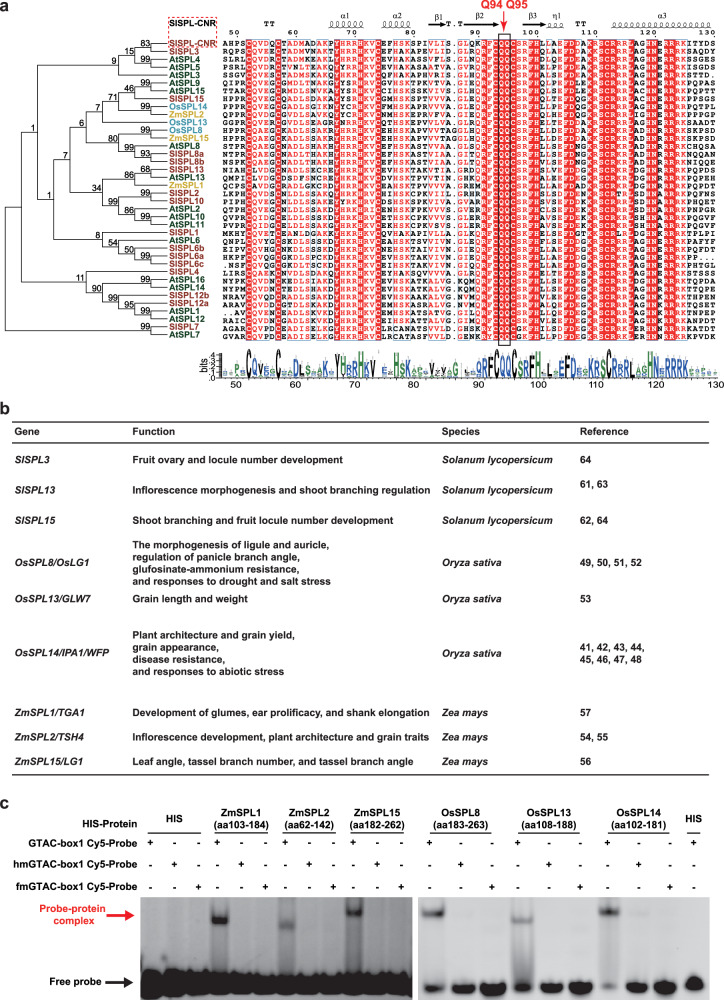
Conserved DNA methylation sensitivity of SBP domains from tomato, rice, and maize. **a** Phylogenetic tree of SPL family members from tomato (16 SPLs), rice (three SPLs), maize (three SPLs), and *Arabidopsis* (16 SPLs). The tree was inferred using the neighbor-joining method in MEGA12 with 1000 bootstrap replicates, and protein sequence conservation of the SBP domain was analyzed using ESPript 3.0. Conserved residues (Q94, Q95) are indicated by red dots. The sequence logo, generated with WebLogo 3, shows amino acid conservation by letter height. **b** Functional annotations of selected *SPL* TFs across tomato, rice, and maize. **c** EMSA demonstrating methylation-sensitive binding of SBP domains from three rice and three maize SBP proteins to unmethylated, hemi-methylated (hm), and fully methylated (fm) GTAC probes (GTAC-box1). His-tagged protein (His) served as the negative control. Experiments were repeated independently two times with similar results. Source data are provided in a Source Data file.

SPL TFs exhibit crucial biological functions across various species, such as tomato, rice, and maize^35,36,84^. For example, in tomato, *SlSPL3*, *SlSPL13*, and *SlSPL15* are involved in fruit ovary and locule development, and shoot branching^61–64^; in rice, *OsSPL8, OsSPL14*, and *OsSPL15* serve as key regulators of plant architecture, grain size, stress responses, and grain yield^47,50,53^; in maize, *ZmSPL1, ZmSPL2*, and *ZmSPL15* are associated with glume and ear development, bract development, and plant architecture^54,56,57^. However, whether and how their DNA-binding activity is modulated by DNA methylation or by the dynamic methylation changes that accompany trait regulation has not been studied. To investigate whether methylation-sensitive binding is conserved among SPL TFs across species, we first conducted a phylogenetic analysis and assessed the sequence conservation of all SPL proteins from tomato and *Arabidopsis*, along with selected agronomically important SPL TFs previously mentioned from rice and maize (Fig. 7a, b and Supplementary Data 10). In the phylogenetic tree, SlSPL-CNR is evolutionarily closer to tomato SlSPL3 and Arabidopsis AtSPL3 (Fig. 7a). Furthermore, alignment of the SBP domain amino acid sequences revealed that key residues Q94 and Q95, which are involved in binding the ‘GTAC’ core motif (Fig. 4d and Supplementary Fig. 4), are highly conserved across species (Fig. 7a). To determine whether the 5-mC repels direct binding of these SBP domains to GTAC motifs, we performed EMSA using unmethylated, hemi-methylated, and fully methylated GTAC-box1 DNA probes. We tested a total of 12 SBP domains from six SlSPLs, three OsSPLs, and three ZmSPL TFs. For all tested SBP domains, we observed inhibited binding of SBP to fmGTAC-box1 and hmGTAC-box1 probes (Fig. 7b and Supplementary Fig. 19). These results indicate that the sensitivity of the SPL-SBP domain in the above three crop species to DNA methylation is similar to that of SlSPL-CNR, suggesting that the recognition of DNA methylation by SPL proteins may be functionally important for agronomic traits across plant species.

## Discussion

DNA methylation is continuously rewritten during development and in response to environmental changes^1,3,4,85^, yet our understanding of how TFs perceive these epigenetic shifts remains fragmentary. Here, we show that the tomato SPL protein SlSPL-CNR is directly repelled by 5-mC within its core motif GTACGG. A single methyl group is sufficient to create a steric clash with Gln94 in the SBP domain, abolishing binding in vitro and reducing occupancy in planta. This establishes SlSPL-CNR as a methylation-sensitive repressor and, to our knowledge, provides the first experimental evidence for methylation-dependent regulation of plant-specific SPL TFs. The sensor residue (Gln94/95) is invariant across angiosperm SPLs, and orthologues from rice (e.g., OsSPL14) and maize (e.g., ZmSPL15) display the same methylation-induced loss of binding (Fig. 7a, c), indicating that the entire agronomically important family is wired to read DNA methylation status.

Previous studies have established the *Cnr* epiallele—defined by hypermethylation within the *SlSPL-CNR* promoter—as a paradigmatic example of epigenetic regulation governing tomato fruit ripening^70^. Intriguingly, loss-of-function mutations in *SlSPL-CNR* do not fully recapitulate the severe ripening defects characteristic of the *Cnr* epiallele, implying that *SlSPL-CNR* deficiency alone cannot account for the complete molecular etiology of the *Cnr* phenotype^86^. Our findings reveal that the conservation of methylation sensitivity across the SBP domain suggests that other tomato *SPL* family members may act redundantly to mitigate methylation-mediated transcriptional repression in the *Cnr* epiallelic background. Future investigations into functional redundancy among SlSPL family members within the hypermethylated context of the *Cnr* epiallele will be critical to fully elucidate this regulatory cascade and hold significant value for dissecting the epigenetic control of fruit ripening.

Integrating DAP-seq—which preserves cytosine methylation but lacks chromatin context—with ChIP-seq and epigenome profiles revealed that SlSPL-CNR occupancy in fruit is governed by at least three layers of epigenetic control: DNA methylation, chromatin accessibility, and histone marks (Fig. 5c–f and Supplementary Fig. 8b). Most DAP unique peaks map to closed or transcriptionally silent regions that are devoid of DHS, H3K4me3, and H3K27ac (Fig. 5c), emphasizing that true regulatory potential can only be gauged when in vitro and in vivo data are combined. Future studies will be required to quantify how each epigenetic layer fluctuates during the rapid phase of ripening.

The biological significance of this methylation-sensitive switch is exemplified by the aroma-related gene *SlAAT1*. During fruit ripening, the DNA glycosylase SlDML2 demethylates the *SlAAT1* promoter, and the resulting hypomethylated GTACGG motifs become accessible for binding by SlSPL-CNR—a methylation-sensitive transcriptional repressor (Fig. 6d and Supplementary Figs. 11 and  15). This binding suppresses *SlAAT1* expression, thereby attenuating ester biosynthesis and fine-tuning fruit aroma (Fig. 6f–h). CRISPR-mediated mutagenesis of *SlSPL-CNR* relieves this repression, leading to elevated *SlAAT1* transcript levels, increased ester volatile accumulation, and broader metabolic shifts, including altered terpenoids, alcohols, and heterocycles (Supplementary Fig. 14d). These results demonstrate that a single methylation-sensitive transcription factor can translate a global epigenetic shift—ripening-associated DNA demethylation—into a metabolite-specific output. Although our volatile profiling was conducted at the breaker stage rather than at the flavor-relevant ripe stage, and sensory evaluation data were not included, the observed upregulation of *SlAAT1* and the concomitant elevation of multiple ester species suggest an enhanced ester biosynthetic capacity in *slcnr-2*. Accordingly, the connection between these metabolic changes and perceptible flavor improvement should be considered putative, and further sensory validation at the fully ripe stage will be required to substantiate this link.

Strikingly, this mechanism challenges the classical view of the association between DNA hypomethylation and gene activation. Instead, our findings reveal a counterintuitive regulatory logic: hypomethylation can actively facilitate the recruitment of a transcriptional repressor, leading to gene silencing. In this context, demethylation of the *SlAAT1* promoter enables SlSPL-CNR-mediated repression, thereby dampening ester formation. This study highlights the context-dependent nature of epigenetic regulation, where the same epigenetic change—promoter demethylation—can lead to opposite transcriptional outcomes depending on the cis-regulatory sequence and the methylation sensitivity of the interacting transcription factor. Given that cultivated tomatoes already show reduced methylation at the *SlAAT1* locus, it is possible that domestication has inadvertently selected for enhanced SlSPL-CNR binding, a hypothesis that can now be tested in diverse germplasm collections. More broadly, this study expands our understanding of how plants integrate epigenetic dynamics with precise metabolic control.

## Methods

### Plant materials and growth conditions

The wild-type (WT) tomato cultivar AC (*Solanum lycopersicum* cv. Ailsa Craig), along with CRISPR/Cas9 knockout mutants (*slcnr-1, slcnr-2*) and transgenic lines (*pCNR::gCNR-3xFLAG* and *pDML2::gDML2-3xFLAG*, all in AC background) were used in this study. Plants were cultivated in a controlled-environment growth chamber under a 16-h light/8-h dark photoperiod, with temperatures of 26–28 °C (day) and 18–20 °C (night) at 65% relative humidity. CRISPR/Cas9 vector construction and plant transformation were performed according to established protocols^87^. Three independent biological replicates (six fruits per replicate) were harvested at each developmental stage. Primer sequences used in this study are provided in Supplementary Data 10.

### Real-time RT-PCR analysis

Total RNA was isolated from tomato fruit tissues using the FastPure Universal Plant Total RNA Isolation Kit (Vazyme, RC411). Complementary DNA was synthesized using the HiScript II Q RT SuperMix for qPCR Kit (Vazyme, R223) according to the manufacturer’s protocol. The cDNA was diluted fivefold; 2 µL of this template was used in 20 µL qPCR reactions containing ChamQ Universal SYBR qPCR Master Mix (Vazyme, Q711). All reactions were performed with three biological replicates.

### DNA affinity purification sequencing (DAP-seq) and ampDAP–seq

DAP-seq was performed following established protocols^71,72,88^. Genomic DNA extracted from ripe WT fruits (46 dpa) using Plant DNAzol Reagent (Invitrogen, 10978021) was sonicated to 250–600 bp fragments (Bioruptor Plus, Diagenode, UCD-300). Fragmented DNA was subjected to end repair (End-It DNA End Repair Kit, Lucigen, ER0720), A-tailing (Klenow Fragment 3′ → 5′ exo-, NEB, M0212L), and Y-adapter ligation (T4 DNA Ligase, Promega, M1804). For ampDAP-seq, 15 ng of adapter-ligated DNA underwent 12 PCR cycles to eliminate methylation. The SlSPL-CNR open reading frame was cloned into a pIX-Halo vector and expressed in vitro using the TNT SP6 Coupled Wheat Germ Extract System (Promega, L4130) at 37 °C for 2 h. Halo-tagged protein was purified using Magna HaloTag Beads (Promega, G7282) for 1 h at room temperature; the empty vector served as a mock control. Protein–DNA-binding reactions containing 150 ng DNA library were incubated at 25 °C for 60 min. Bound DNA was eluted at 95 °C for 10 min and amplified with indexed Illumina primers using Phanta Max Super-Fidelity DNA Polymerase (Vazyme, P521). PCR products were purified and size-selected (200–600 bp) with VAHTS DNA Clean Beads (Vazyme, N411) prior to sequencing on an Illumina NovaSeq 6000 platform (Core Facility for Genomics, Shanghai Center for Plant Stress Biology).

### Whole-genome bisulfite sequencing (WGBS) and data analysis

Whole-genome DNA was extracted from fruits of WT plants at different developmental stages using the Plant DNeasy Mini Kit (QIAGEN, 69106). The extracted genomic DNA was then submitted to the Genomics Core Facility at the Shanghai Centre for Plant Stress Biology, Chinese Academy of Sciences, for whole-genome bisulfite conversion, library construction, and high-throughput sequencing on an Illumina NovaSeq 6000 platform.

Raw 150 bp paired-end reads were quality-controlled using fastp (v0.12.4)^89^ to remove adapter sequences and trim low-quality bases (Phred quality score < Q15). Subsequent quality assessment was performed using FastQC (v0.11.9)^90^ to confirm complete adapter removal. The cleaned reads were aligned to the tomato reference genome (SL4.0) using Bismark (v0.23.0)^91^ with the parameter “-N 1” to allow one nucleotide mismatch during seed alignment. PCR duplicates were removed using the deduplicate_bismark module within Bismark, and methylation calls for each cytosine context (CpG, CHG, CHH) were extracted using the bismark_methylation_extractor module. Only cytosines covered by a minimum of four reads were retained for downstream analysis. The methylation level of each cytosine was calculated as the number of reads containing a cytosine (C) divided by the total reads covering that position (C + T (thymine)). The average methylation level of a genomic region was calculated using the following formula:

$$\frac{\sum C}{\sum (C+T)}$$

Bisulfite conversion efficiency was assessed by calculating the non-conversion rate of cytosines in chloroplast DNA, which lacks methylation and thus serves as an internal control. Methylation calls information was converted to BedGraph format and subsequently transformed into BigWig files using bedGraphToBigwig (v2.8) from UCSC tools for visualization in the Integrative Genomics Viewer (IGV)^92^. Differential methylation analysis was performed using the R package methylKit^93^. Methylation differences between WT-Br and WT-green were assessed with the calculateDiffMeth function. Cytosines with a *q* value < 0.05 were considered significantly differentially methylated. Context-specific methylation difference thresholds (≥ 20% for CG, ≥ 10% for CHG (H = A, T, or C), and ≥ 5% for CHH) were applied to classify hyper- and hypomethylated cytosines, which were collectively defined as differentially methylated cytosines (DMCs).

### Electrophoretic mobility shift assay (EMSA)

EMSA was performed following established protocol^94^. The ORF of *SlSPL-CNR* and all SBP domains sequences employed in this study were amplified and cloned to pET-Sumo vector. These vectors were transferred to *Escherichia coli* (BL21) and incubated at 16 °C for 16 h. Proteins were purified using His-tag Purification Resin (BeyoGold, P2221) as instruction described. DNA probes were 5′-end labeled with Cy5 or FAM. Labeled probes and purified proteins were incubated at 30 °C for 20 min in binding buffer (18 mM Tris–HCl, 4.5% glycerol, 16 mM MgCl₂, 1.6 mM DTT, 35.4 ng/µL BSA). Reaction mixtures were resolved at 4 °C on 6% native polyacrylamide gels in 0.5× TBE buffer at 100 V for 1.5 h. Gels were visualized using a FLA-9000 scanner (Fujifilm, Japan).

### Isothermal titration calorimetry (ITC) assay

ITC analyses were conducted using previously validated experimental procedures^95^. The ORF of SlSPL-CNR and its SBP domains were cloned into pET-Sumo vector. Recombinant proteins were expressed in *Escherichia coli* (BL21) and purified using His-tag Purification Resin (BeyoGold, P2221) according to the manufacturer’s protocol. The His-Sumo tag was removed by Ulp1 cleavage followed by secondary affinity purification. Purified proteins were dialyzed against buffer (100 mM NaCl, 20 mM Tris–HCl, pH 8.0, 2 mM β-mercaptoethanol) and adjusted to 40–50 µM. DNA oligonucleotides were resuspended in the same buffer at 700–900 µM. Titrations were performed at 20 °C using a MicroCal PEAQ-ITC (Malvern Panalytical, UK), and data were analyzed with Origin 7.0 software. Detailed data are listed in Supplementary Data 3.

### Chromatin immunoprecipitation (ChIP) sequencing

ChIP-seq was performed on WT and *pCNR::gCNR-3xFLAG* transgenic fruits harvested at the green (30 dpa) and breaker (38 dpa) stages. For Tag lines analyzed by western blotting, the concentrated protein extracts from 0.2 g samples were mixed with 6× SDS–PAGE loading buffer, denatured at 95 °C for 10 min, separated on a 10% SDS–PAGE gel, and transferred to nitrocellulose membranes. Membranes were incubated overnight with anti-flag (Sigma-Aldrich, F3165,1:5000) or anti-Actin (plant-specific) (ABclonal, AC009, 1:10,000), followed by anti-mouse secondary antibodies (ABclonal, #AS003, 1:10,000). Signals were detected using the Basic ECL ElectroChemiluminescence Kit (ShareBio, WB001).

Chromatin immunoprecipitation was conducted according to established protocol with minor modifications^96^. Fruit pericarp tissues were crosslinked with 1% formaldehyde for 10 min and quenched with 0.2 M glycine for 5 min at room temperature. Approximately 0.3 g of tissue was used per ChIP assay. Samples were ground in liquid nitrogen, lysed in 800 μL Buffer S (50 mM HEPES–KOH pH 7.5, 150 mM NaCl, 1 mM EDTA, 1% Triton X-100, 0.1% sodium deoxycholate, 1% SDS and 1× protease inhibitor) for 10 min at 4 °C, then diluted with 1.6 mL Buffer F (same as Buffer S but without SDS) and sonicated to fragment chromatin to 300–700 bp. One percent of the supernatant was used as the input sample (Input). For each ChIP experiment, 50 μL Protein G Dynabeads (Invitrogen, 10003D) were washed twice with 1 mL PBST buffer (1× PBS, 0.1% Tween 20), bound with 10 µg anti-FLAG M2 antibody (Sigma, F3165) for 6 h at 4 °C, and washed twice before incubating overnight with precleared fragment chromatin. Immunoprecipitated complexes were washed sequentially with low-salt ChIP buffer (50 mM HEPES–KOH, 150 mM NaCl, 1 mM EDTA, 1% Triton X-100, 0.1% sodium deoxycholate, 0.1% SDS; twice), high-salt ChIP buffer (replaced NaCl concentration with 350 mM; twice), LiCl wash buffer (10 mM Tris–HCl pH 8.0, 250 mM LiCl, 0.5% NP-40, 1 mM EDTA, 0.1% sodium deoxycholateonce; once), and TE buffer (once). Elution was performed with 100 μL ChIP Elution buffer (50 mM Tris–HCl pH 7.5, 10 mM EDTA, 1% SDS) at 65 °C for 15 min with agitation at 900 rpm. Eluates (ChIP or Input) were pooled, treated with 5 μL proteinase K at 55 °C overnight to reverse crosslinks, and purified using a commercial DNA Clean & Concentrator-5 kit (Zymo Research, D4014). ChIP DNA (> 0.1 ng) was used to construct sequencing libraries with the Scale ssDNA-seq Lib Prep Kit for Illumina V2 (ABclonal, RK20228). Libraries were sequenced on an Illumina NovaSeq 6000 platform (GENEWIZ, Suzhou, China) to generate 150-bp paired-end reads.

For ChIP-qPCR on WT and *pDML2::gDML2-3xFLAG*, enrichment of immunoprecipitated DNA fragments was quantified by quantitative real-time PCR. Purified DNA fragments (ChIP or Input) were diluted fivefold, and 2 µL of the dilution served as template in a 20 µL qPCR reaction.

### Processing of DAP/ampDAP-seq, ChIP-seq, and DNase I hypersensitive site sequencing (DHS) data

Raw paired-end reads from DAP/ampDAP-seq, ChIP-seq, and DNase-seq data were processed using fastp (v0.12.4)^89^ to trim adapters and low-quality bases, followed by quality assessment using FastQC (v0.11.9)^90^. Clean reads were aligned to the tomato reference genome (SL4.0) using Bowtie2 (v2.2.5)^97^ under default parameters. Reads with mapping quality < 20 were filtered using samtools (v1.19)^98^, and PCR duplicates were removed with Sambamba (v0.6.6)^99^. Visualization files were generated using the bamCoverage tool from deepTools (v3.0.1)^100^ with RPKM normalization.

For ChIP-seq and DAP-seq data, peak calling was performed with MACS2 (v2.2.7.1)^101^ with the parameters “--nomodel --nolambda”. For DAP-seq, peaks were retained at *P* ≤ 1×10⁻¹⁵ and fold change ≥ 5, while for ChIP-seq, peaks were filtered at *P* ≤ 1×10⁻⁵ and fold change ≥ 5. Distinct thresholds were applied since DAP-seq typically yields stronger enrichment and lower background than ChIP-seq. For motif analysis, the top 600 peaks ranked by −10 × log_10_ (*q* value) were selected, and 200 bp sequences centered on peak summits (±100 bp) were extracted using bedtools (v2.27.1)^102^. De novo motif discovery was carried out using MEME-ChIP of the MEME software toolkit (v4.11.2)^103^, and the top enriched motifs with the highest statistical significance (lowest *E* value) were aligned and visualized using the R package motifStack^104^. The motifs identified by MEME-ChIP were used as input for the FIMO program of the MEME software toolkit to scan the entire genome for motif-matched sites. Note that motif scanning with FIMO evaluates each motif independently, resulting in overlapping occurrences between core motifs and their nested sub-motif variants. These overlaps are an inherent feature of the algorithm and were intentionally retained unmerged to preserve the biological resolution of flanking sequence preferences.

### Comparison of DAP-seq and ampDAP-seq

To compare the ampDAP-seq and DAP-seq data, we assumed that signal differences between the two assays primarily resulted from differing levels of DNA methylation. Accordingly, we performed a differential binding analysis to enable a direct comparison. Specifically, 200 bp genomic regions centered on peak summits (±100 bp) were extracted from both CNR-DAP and CNR-ampDAP datasets and merged into a non-redundant set of 213,326 peak regions. For each region, we quantified the binding signals from both datasets and the corresponding average methylation level. Normalization factors were then estimated using the R package DESeq2 based on signals in unmethylated regions (5-mC ≤ 0.1) and were applied to normalize the entire dataset for downstream differential binding analysis.

### Crystallization, data collection, and structural determination

Prior to crystallization, the purified SlSPL-CNR SBP domain (residues 46–130) was concentrated to 10 mg/ml and mixed with an 11-bp DNA duplex with a molar ratio of 1:1.2. The crystal screening was carried out using the sitting-drop vapor diffusion method at 20 °C. The SBP–DNA complex was crystallized in a condition of 20% PEG 1000 and 0.1 M Tris–HCl, pH 8.5. Before data collection, the crystals were soaked into the reservoir solution supplemented with 15% glycerol and flash-cooled in liquid nitrogen. The data were collected in Shanghai Synchrotron Radiation Facilities beamline BL19U1 and processed using the program HKL3000^105,106^. The structure was determined using the molecular replacement method using the program Phenix with the structure of Arabidopsis SBP-Like 12 (PDB code: 1WJ0) as a search model^107^. Models were refined using the program Phenix and were manually built in the program COOT^107,108^. The structures were finally presented using the program PyMOL (Schrödinger, Inc.).

### PR analysis

The predictive performance of DAP-seq peaks—both overall and those overlapping with DNase I hypersensitive sites (DHS)—was evaluated using PR curves and the corresponding area under the curve (AUPRC), following a previous study^72^. Motif scores, derived from FIMO^109^ scanning results, served as quantitative indicators of peak confidence. Each peak was assigned the summed motif match score of all overlapping motif occurrences. Both the set of all DAP-seq peaks and the subset overlapping DHS regions were ranked in descending order based on their motif scores and treated as the predicted sets. The ChIP-seq peaks served as the ground truth set. The ranked peaks were stratified into bins based on their motif scores. Precision and recall were calculated for each bin using the following definitions:

$${{{\rm{Precision}}}}=\frac{{TP}}{{TP}+{FP}}$$

$${{{\rm{Recall}}}}=\frac{{TP}}{{TP}+{FN}}$$

Where *TP* (true positives) represents DAP-seq peaks that overlap with ChIP-seq peaks, *FP* (false positives) denotes DAP-seq peaks without a corresponding ChIP-seq peak, and *FN* (false negatives) corresponds to ChIP-seq peaks not captured by DAP-seq. The resulting PR curves and AUPRC values were used to quantitatively compare the performance of the two prediction sets (all DAP-seq peaks vs. DAP-seq peaks within DHS regions).

### Gene set enrichment analysis (GSEA)

Due to differences in ChIP-seq enrichment efficiency between green fruit and breaker (Br) fruit tissues, we performed a differential binding analysis to enable a meaningful comparison between the CNR-GreenChIP and CNR-BrChIP datasets. We hypothesized that binding signals would remain stable across the 2662 stable-C_4_ sites (|5-mC difference| ≤ 0.1), where DNA methylation levels show minimal change. Based on this assumption, normalization factors were derived from the median signal intensities within these stable-C_4_ sites and subsequently applied to normalize the hypo-C_4_ sites for subsequent analysis.

GSEA^110^ was used to assess whether dynamic DNA methylation changes were associated with SlSPL-CNR binding signals in vivo. For the 3080 GTACGG-matched sites, the log_2_ fold change in binding signals between CNR-BrChIP and CNR-GreenChIP was calculated. These sites were then ranked according to their log_2_ fold change values. The ranked list served as the input for a GSEA-like analysis implemented using the fgsea R package, with the following parameters: 10,000 permutations, a minimum gene set size of 15, and a maximum size of 2000. The set of 190 hypo-C4 sites was treated as a predefined gene set. Enrichment scores (ES) were computed to test whether these hypo-C4 sites were preferentially enriched among genomic loci exhibiting enhanced SlSPL-CNR binding. Finally, KEGG pathway analysis was conducted on the genes associated with the leading-edge subset of sites using TBtools tool^111,112^.

### Dual-luciferase reporter assay

Transactivation assays were performed following the established protocol^94^. The ORF of *SlSPL-CNR* and other sequences were cloned into pGreenII 62-SK (SK), and the 1586-bp *SlAAT1* promoter into pGreenII 0800-LUC (LUC). *Agrobacterium tumefaciens* harboring both constructs was co-infiltrated into *Nicotiana benthamiana* leaves. Luciferase activities were quantified using the Dual-Luciferase Reporter Assay Kit (Vazyme, DL101) on an EnSight Multimode Plate Reader (PerkinElmer, USA). Firefly luciferase (LUC) activity was normalized to Renilla luciferase (REN) internal control. All experiments included at least six biological replicates.

### Headspace solid-phase microextraction coupled with gas chromatography–mass spectrometry (HS-SPME/GC-MS) analysis

Fruit samples at the breaker stage from WT and *slcnr-2* mutants were pulverized in liquid nitrogen. Subsequently, 500 mg of the powdered sample was transferred to a 20 mL headspace vial (Agilent) containing 2 mL saturated NaCl solution to suppress enzymatic activity. Vials were sealed with crimp-top caps fitted with TFE-silicone septa. For HS-SPME, vials were incubated at 60 °C for 5 min, followed by exposure of a 120-µm DVB/CWR/PDMS SPME Arrow to the headspace for 15 min at 60 °C. Following extraction, adsorbed VOCs were thermally desorbed in the GC injection port (Model 8890, Agilent) at 250 °C for 5 min. VOCs were analyzed using an Agilent 8890 GC system coupled with a 7000D mass spectrometer (Agilent), equipped with a DB-5MS column (30 m × 0.25 mm × 0.25 µm). Helium carrier gas was delivered at 1.2 mL/min. The injector temperature was maintained at 250 °C. The oven temperature program was as follows: 40 °C (3.5 min), then 10 °C/min to 100 °C, 7 °C/min to 180 °C, 25 °C/min to 280 °C, held for 5 min. Mass spectra were recorded in electron impact (EI) mode (70 eV). Temperatures of the quadrupole detector, ion source, and transfer line were set to 150 °C, 230 °C, and 280 °C, respectively. Analytes were identified and quantified in selected ion monitoring (SIM) mode.

Data were analyzed by Metware Biotechnology (Wuhan, China). Unsupervised PCA (principal component analysis) performed in R (www.r-project.org) (v4.1.2) using the prcomp function from the base package, with unit-variance scaling applied prior to analysis. Hierarchical cluster analysis (HCA) and Pearson correlation coefficients (PCC) were calculated using the R package ComplexHeatmap (v2.9.4) with unit variance scaling normalized signal intensities. Differential metabolites were identified using VIP > 1 and |log₂FC | ≥ 1.0. VIP values were extracted from the OPLS-DA results, which also include score plots and permutation plots, and were generated using the R package MetaboAnalystR (v1.0.1). The data were log₂-transformed and mean-centered prior to OPLS-DA. To avoid overfitting, a permutation test (200 permutations) was performed. Detailed data are provided in Supplementary Data 8 and 9.

### Reporting summary

Further information on research design is available in the Nature Portfolio Reporting Summary linked to this article.

## Supplementary information

Supplementary Information

Description of Additional Supplementary Information

Supplementary Data 1

Supplementary Data 2

Supplementary Data 3

Supplementary Data 4

Supplementary Data 5

Supplementary Data 6

Supplementary Data 7

Supplementary Data 8

Supplementary Data 9

Supplementary Data 10

Reporting Summary

Transparent Peer Review file

## Source data

Source Data

## Data Availability

The WGBS (WT-Green/Red) data generated in this study have been deposited in the Gene Expression Omnibus (GEO) under accession code GSE173212. The WGBS raw data of *slcnr* have been deposited in the Sequence Read Archive (SRA) under accession code PRJNA1452455. The DAP-seq (CNR/Halo-DAP) data are available under accession code GSE329250. The ampDAP-seq (CNR/Halo-ampDAP) raw data are available under accession code PRJNA1320814. The ChIP-seq raw data generated in this study are accessible under accession code PRJNA1315166. The DHS (SRR5405286), H3K4me3 ChIP-seq (SRR7647220), and H3K27ac ChIP-seq (SRR7647238) raw data were obtained from a previously published study^79^. The WGBS and RNA data of *dml2* and AC at 25- and 34-dpa (WT-Br) were obtained from the study^16^ (GSE210590). The coordinates and structure factors have been deposited in the Protein Data Bank under accession code 21CH. Source data are provided with this paper.
