# Supplementary material for: DNA hypomethylation enables the transcriptional repressor SlSPL-CNR to control fruit flavor ester biosynthesis
Source: Nat Commun. 2026 Jul 1;17:8168. doi: 10.1038/s41467-026-75181-8 (PMC13458263; doi:10.1038/s41467-026-75181-8)
Supplement: Supplementary file 1 [file 41467_2026_75181_MOESM13_ESM.pdf]

nature portfolio

Corresponding author(s): Zhaobo Lang

Last updated by author(s): Jun 19, 2026

Reporting Summary

Nature Portfolio wishes to improve the reproducibility of the work that we publish. This form provides structure for consistency and transparency in reporting. For further information on Nature Portfolio policies, see our Editorial Policies and the Editorial Policy Checklist.

Statistics

For all statistical analyses, confirm that the following items are present in the figure legend, table legend, main text, or Methods section.

n/a	Confirmed	
☐	☒	The exact sample size (n) for each experimental group/condition, given as a discrete number and unit of measurement
☐	☒	A statement on whether measurements were taken from distinct samples or whether the same sample was measured repeatedly
☐	☒	The statistical test(s) used AND whether they are one- or two-sided<br>*Only common tests should be described solely by name; describe more complex techniques in the Methods section.*
☐	☒	A description of all covariates tested
☐	☒	A description of any assumptions or corrections, such as tests of normality and adjustment for multiple comparisons
☐	☒	A full description of the statistical parameters including central tendency (e.g. means) or other basic estimates (e.g. regression coefficient) AND variation (e.g. standard deviation) or associated estimates of uncertainty (e.g. confidence intervals)
☐	☒	For null hypothesis testing, the test statistic (e.g. F, t, r) with confidence intervals, effect sizes, degrees of freedom and P value noted<br>*Give P values as exact values whenever suitable.*
☒	☐	For Bayesian analysis, information on the choice of priors and Markov chain Monte Carlo settings
☒	☐	For hierarchical and complex designs, identification of the appropriate level for tests and full reporting of outcomes
☐	☒	Estimates of effect sizes (e.g. Cohen's d, Pearson's r), indicating how they were calculated

Our web collection on statistics for biologists contains articles on many of the points above.

Software and code

Policy information about availability of computer code

Data collection	DAP/ampDAP-seq, Bisulfite-seg, and ChIP-seq libraries were sequenced on the Illumina NovaSeq 6000. ITC experiments were conducted at MicroCal PEAQ-ITC (Malvern Panalytical, UK). The difraction data were colected at the Shanghai Synchrotron Radiation Facilities beamline BL19U1 and processed using the program HKL3000.. Luciferase activities were measured using an EnSight Multimode Plate Reader (PerkinElmer, USA). Adsorbed VOCs were thermally desorbed from a 120-µm DVB/CAR/PDMS SPME Arrow into the injection port of an Agilent 8890 GC.
Data analysis	fastp (v0.12.4)<br>FastQC (v0.11.9)<br>Bismark (v0.23.0)<br>bedGraphToBigwig (v2.8)<br>R package (methylKit)<br>Bowtie2 (v2.2.5)<br>samtools (v1.19)<br>Sambamba (v0.6.6)<br>deepTools (v3.0.1)<br>MACS2 (v2.2.7.1)<br>bedtools (v2.27.1)<br>MEME software toolkit (v4.11.2)<br>R package (motifStack)<br>R package (DESeq2)

TBtools (v2.441)
Origin7.0

For HS-SPME/GC-MS data analysis, unsupervised PCA (principal component analysis) performed in R (www.r-project.org) (v4.1.2) using the prcomp function from base package, with unit-variance scaling applied prior to analysis. Hierarchical cluster analysis (HCA) and Pearson correlation coefficients (PCC) were calculated using the R package ComplexHeatmap (v2.9.4) with unit variance scaling normalized signal intensities. Differential metabolites were identified using VIP > 1 and $|log_2FC| \geq 1.0$. VIP values were extracted from the OPLS-DA results, which also include score plots and permutation plots and were generated using the R package MetaboAnalystR (v1.0.1). The data were log_2-transformed and mean-centered prior to OPLS-DA. To avoid overfitting, a permutation test (200 permutations) was performed.

Raw paired-end reads from DAP/ampDAP-seq and ChIP-seq were processed using fastp (v0.12.4) to trim adapters and low-quality bases, followed by quality assessment using FastQC (v0.11.9). Clean reads were aligned to the tomato reference genome (SL4.0) using Bowtie2 (v2.2.5) under default parameters. Reads with mapping quality < 20 were filtered using samtools (v1.19), and PCR duplicates were removed with Sambamba (v0.6.6). Visualization files were generated using the bamCoverage tool from deepTools (v3.0.1) with RPKM normalization.For ChIP-seq and DAP-seq data, peak calling was performed with MACS2 (v2.2.7.1) with the parameters "--nomodel --nolambda". For DAP-seq, peaks were retained at $p \leq 1\times10^{-15}$ and fold change ≥ 5, while for ChIP-seq, peaks were filtered at $p \leq 1\times10^{-5}$ and fold change ≥ 5. Distinct thresholds were applied since DAP-seq typically yields stronger enrichment and lower background than ChIP-seq. For motif analysis, the top 600 peaks ranked by -10×log10 (q-value) were selected, and 200 bp sequences centered on peak summits (± 100 bp) were extracted using bedtools (v2.27.1). De novo motif discovery was carried out using MEME-ChIP of the MEME software toolkit (v4.11.2), and the top enriched motifs with the highest statistical significance (lowest E-value) were aligned and visualized using the R package motifStack. The motifs identified by MEME-ChIP were used as input for FIMO program of the MEME software toolkit to scan the entire genome for motif-matched sites.

For manuscripts utilizing custom algorithms or software that are central to the research but not yet described in published literature, software must be made available to editors and reviewers. We strongly encourage code deposition in a community repository (e.g. GitHub). See the Nature Portfolio guidelines for submitting code & software for further information.

Data

Policy information about availability of data

All manuscripts must include a data availability statement. This statement should provide the following information, where applicable:

- Accession codes, unique identifiers, or web links for publicly available datasets
- A description of any restrictions on data availability
- For clinical datasets or third party data, please ensure that the statement adheres to our policy

The WGBS (WT-Green/Red) data generated in this study have been deposited in the Gene Expression Omnibus (GEO) under accession code GSE173212 [https://www.ncbi.nlm.nih.gov/geo/query/acc.cgi?acc=GSE173212]. The WGBS raw data of slcnr have been deposited in the Sequence Read Archive (SRA) under accession code PRJNA1452455 [https://www.ncbi.nlm.nih.gov/bioproject/PRJNA1452455]. The DAP-seq (CNR/Halo-DAP) data are available under accession code GSE329250 [https://www.ncbi.nlm.nih.gov/geo/query/acc.cgi?acc=GSE329250]. The ampDAP-seq (CNR/Halo-ampDAP) raw data are available under accession code PRJNA1320814 [https://www.ncbi.nlm.nih.gov/bioproject/PRJNA1320814]. The ChIP-seq raw data generated in this study are accessible under accession code PRJNA1315166 [https://www.ncbi.nlm.nih.gov/bioproject/PRJNA1315166]. The DHS (SRR5405286), H3K4me3 ChIP-seq (SRR7647220), and H3K27ac ChIP-seq (SRR7647238) raw data were obtained from a previously published study. The WGBS and RNA data of dml2 and AC at 25- and 34-dpa (WT-Br) were obtained from the study (GSE210590). The coordinates and structure factors have been deposited in the Protein Data Bank under accession code 21CH. Source data are provided with this paper.

Research involving human participants, their data, or biological material

Policy information about studies with human participants or human data. See also policy information about sex, gender (identity/presentation), and sexual orientation and race, ethnicity and racism.

Reporting on sex and gender	*Use the terms sex (biological attribute) and gender (shaped by social and cultural circumstances) carefully in order to avoid confusing both terms. Indicate if findings apply to only one sex or gender; describe whether sex and gender were considered in study design; whether sex and/or gender was determined based on self-reporting or assigned and methods used.*<br>*Provide in the source data disaggregated sex and gender data, where this information has been collected, and if consent has been obtained for sharing of individual-level data; provide overall numbers in this Reporting Summary. Please state if this information has not been collected.*<br>*Report sex- and gender-based analyses where performed, justify reasons for lack of sex- and gender-based analysis.*
Reporting on race, ethnicity, or other socially relevant groupings	*Please specify the socially constructed or socially relevant categorization variable(s) used in your manuscript and explain why they were used. Please note that such variables should not be used as proxies for other socially constructed/relevant variables (for example, race or ethnicity should not be used as a proxy for socioeconomic status).*<br>*Provide clear definitions of the relevant terms used, how they were provided (by the participants/respondents, the researchers, or third parties), and the method(s) used to classify people into the different categories (e.g. self-report, census or administrative data, social media data, etc.)*<br>*Please provide details about how you controlled for confounding variables in your analyses.*
Population characteristics	*Describe the covariate-relevant population characteristics of the human research participants (e.g. age, genotypic information, past and current diagnosis and treatment categories). If you filled out the behavioural & social sciences study design questions and have nothing to add here, write "See above."*
Recruitment	*Describe how participants were recruited. Outline any potential self-selection bias or other biases that may be present and how these are likely to impact results.*

Ethics oversight | Identify the organization(s) that approved the study protocol.

Note that full information on the approval of the study protocol must also be provided in the manuscript.

Field-specific reporting

Please select the one below that is the best fit for your research. If you are not sure, read the appropriate sections before making your selection.

[x] Life sciences [ ] Behavioural & social sciences [ ] Ecological, evolutionary & environmental sciences

For a reference copy of the document with all sections, see nature.com/documents/nr-reporting-summary-flat.pdf

Life sciences study design

All studies must disclose on these points even when the disclosure is negative.

Sample size	Gene expression, WGBS, ChIP-seq, Dual-luc and genome for DAP-seq: at least six plants were selected for each genotype.
Data exclusions	No data was excluded.
Replication	For DAP-seq and ampDAP-seq, HS-SPME coupled with GC-MS, and qRT-PCR analyses, three biological replicates were performed to ensure experimental reproducibility. For ChIP-seq and whole-genome bisulfite sequencing (WGBS), two biological replicates were used. For the dual-luciferase reporter assay, six biological replicates were conducted.
Randomization	Plants samples were grown in same environmental conditions. We used randomizationto allocate different samples.
Blinding	Blinding is not applicable in this study. There was no subjective bias in this study

Reporting for specific materials, systems and methods

We require information from authors about some types of materials, experimental systems and methods used in many studies. Here, indicate whether each material, system or method listed is relevant to your study. If you are not sure if a list item applies to your research, read the appropriate section before selecting a response.

Materials & experimental systems

n/a	Involved in the study	
[ ]	[x]	Antibodies
[x]	[ ]	Eukaryotic cell lines
[x]	[ ]	Palaeontology and archaeology
[x]	[ ]	Animals and other organisms
[x]	[ ]	Clinical data
[x]	[ ]	Dual use research of concern
[ ]	[x]	Plants

Methods

n/a	Involved in the study	
[ ]	[x]	ChIP-seq
[x]	[ ]	Flow cytometry
[x]	[ ]	MRI-based neuroimaging

Antibodies

Antibodies used	Magne Halo-Tag Beads(Promega,#G7282); anti-flag (Sigma-Aldrich, #F3 165);anti-Actin (plant specific) (ABclonal, #AC009); anti-Mouse (ABclonal, #AS003)
Validation	The specifcity of antibodies used in this paper was extensivelv examined by the vendor and independent investigators.<br>Magne Halo-Tag Beads(Promega,#G7282): https://www.promega.com.cn/products/protein-purification/protein-purification-kits/magne-halotag-beads/?catNum=G7282<br>anti-flag (Sigma-Aldrich, #F3165): https://www.sigmaaldrich.cn/CN/zh/product/sigma/F3165<br>anti-Actin (plant specific) (ABclonal, #AC009): https://abclonal.com.cn/catalog/AC009<br>anti-Mouse (ABclonal, #AS003): https://abclonal.com.cn/catalog/AS003

Dual use research of concern

Policy information about dual use research of concern

Hazards

Could the accidental, deliberate or reckless misuse of agents or technologies generated in the work, or the application of information presented in the manuscript, pose a threat to:

No | Yes

- [x] No [ ] Yes — Public health
- [x] No [ ] Yes — National security
- [x] No [ ] Yes — Crops and/or livestock
- [x] No [ ] Yes — Ecosystems
- [x] No [ ] Yes — Any other significant area

Experiments of concern

Does the work involve any of these experiments of concern:

No | Yes

- [x] No [ ] Yes — Demonstrate how to render a vaccine ineffective
- [x] No [ ] Yes — Confer resistance to therapeutically useful antibiotics or antiviral agents
- [x] No [ ] Yes — Enhance the virulence of a pathogen or render a nonpathogen virulent
- [x] No [ ] Yes — Increase transmissibility of a pathogen
- [x] No [ ] Yes — Alter the host range of a pathogen
- [x] No [ ] Yes — Enable evasion of diagnostic/detection modalities
- [x] No [ ] Yes — Enable the weaponization of a biological agent or toxin
- [x] No [ ] Yes — Any other potentially harmful combination of experiments and agents

Plants

Seed stocks

The seeds of tomato (Solanum lycopersicum cv. Ailsa Craig) and N. benthamiana are both stored in our lab.

Novel plant genotypes

Not applicable for this study.

Authentication

Not applicable for this study.

ChIP-seq

Data deposition

- [x] Confirm that both raw and final processed data have been deposited in a public database such as GEO.
- [x] Confirm that you have deposited or provided access to graph files (e.g. BED files) for the called peaks.

Data access links
May remain private before publication.

The WGBS (WT-Green/Red) data generated in this study have been deposited in the Gene Expression Omnibus (GEO) under accession code GSE173212 [https://www.ncbi.nlm.nih.gov/geo/query/acc.cgi?acc=GSE173212]. The WGBS raw data of slcnr have been deposited in the Sequence Read Archive (SRA) under accession code PRJNA1452455 [https://www.ncbi.nlm.nih.gov/bioproject/PRJNA1452455]. The DAP-seq (CNR/Halo-DAP) data are available under accession code GSE329250 [https://www.ncbi.nlm.nih.gov/geo/query/acc.cgi?acc=GSE329250]. The ampDAP-seq (CNR/Halo-ampDAP) raw data are available under accession code PRJNA1320814 [https://www.ncbi.nlm.nih.gov/bioproject/PRJNA1320814]. The ChIP-seq raw data generated in this study are accessible under accession code PRJNA1315166 [https://www.ncbi.nlm.nih.gov/bioproject/PRJNA1315166].

Files in database submission

Provide a list of all files available in the database submission.

Genome browser session
(e.g. UCSC)

Not applicable for this study.

Methodology

Replicates

For DAP-seq and ampDAP-seq, three biological replicates were performed to ensure experimental reproducibility. For ChIP-seq, two biological replicates were used.

Sequencing depth

CNR-DAP-1: 15498501 pairs
CNR-DAP-2: 17433173 pairs
CNR-DAP-3: 17617336 pairs
Halo-DAP-1: 11579483 pairs

Halo-DAP-2: 10559950 pairs
Halo-DAP-3: 10428664 pairs
CNR-ampDAP-1: 21233056 pairs
CNR-ampDAP-2: 23330255 pairs
CNR-ampDAP-3: 23613230 pairs
Halo-ampDAP-1: 10078724 pairs
Halo-ampDAP-2: 15583172 pairs
Halo-ampDAP-3: 19661224 pairs
AC30_ChIP_2: 25765882 pairs
AC30_ChIP_1: 38123359 pairs
AC_Br_ChIP_1: 24776278 pairs
AC_Br_ChIP_2: 36963335 pairs
CNR_Br_ChIP_1: 28633311 pairs
CNR_Br_ChIP_2: 31454890 pairs
CNR_Br_input_2: 36382867 pairs
CNR_Br_input_1: 36753104 pairs
AC_Br_input_1: 34934890 pairs
AC_Br_input_2: 37356646 pairs
CNR30_input_1: 39234967 pairs
CNR30_input_2: 37775973 pairs
AC30_input_1: 40093056 pairs
CNR30_ChIP_1: 46463314 pairs
AC30_input_2: 55146638 pairs
CNR30_ChIP_2: 60318281 pairs

Antibodies

anti-flag (Sigma-Aldrich, #F3165): https://www.sigmaaldrich.cn/CN/zh/product/sigma/F3165

Peak calling parameters

For DAP-seq, peaks were retained at $p \leq 1\times10^{-15}$ and fold change ≥ 5, while for ChIP-seq, peaks were filtered at $p \leq 1\times10^{-5}$ and fold change ≥ 5.

Data quality

NA

Software

Raw paired-end reads from DAP/ampDAP-seq and ChIP-seq were processed using fastp (v0.12.4) to trim adapters and low-quality bases, followed by quality assessment using FastQC (v0.11.9). Clean reads were aligned to the tomato reference genome (SL4.0) using Bowtie2 (v2.2.5) under default parameters. Reads with mapping quality < 20 were filtered using samtools (v1.19), and PCR duplicates were removed with Sambamba (v0.6.6). Visualization files were generated using the bamCoverage tool from deepTools (v3.0.1) with RPKM normalization.For ChIP-seq and DAP-seq data, peak calling was performed with MACS2 (v2.2.7.1) with the parameters "--nomodel --nolambda". For DAP-seq, peaks were retained at $p \leq 1\times10^{-15}$ and fold change ≥ 5, while for ChIP-seq, peaks were filtered at $p \leq 1\times10^{-5}$ and fold change ≥ 5. Distinct thresholds were applied since DAP-seq typically yields stronger enrichment and lower background than ChIP-seq. For motif analysis, the top 600 peaks ranked by -10×log10 (q-value) were selected, and 200 bp sequences centered on peak summits (± 100 bp) were extracted using bedtools (v2.27.1). De novo motif discovery was carried out using MEME-ChIP of the MEME software toolkit (v4.11.2), and the top enriched motifs with the highest statistical significance (lowest E-value) were aligned and visualized using the R package motifStack. The motifs identified by MEME-ChIP were used as input for FIMO program of the MEME software toolkit to scan the entire genome for motif-matched sites.
