# Supplementary material for: DNA hypomethylation enables the transcriptional repressor SlSPL-CNR to control fruit flavor ester biosynthesis
Source: Nat Commun. 2026 Jul 1;17:8168. doi: 10.1038/s41467-026-75181-8 (PMC13458263; doi:10.1038/s41467-026-75181-8)
Supplement: Supplementary file 2 [file 41467_2026_75181_MOESM14_ESM.pdf]

nature portfolio

Peer Review File

DNA hypomethylation enables the transcriptional repressor SlSPL-CNR to control fruit flavor ester biosynthesis

Corresponding Author: Professor Zhaobo Lang

This file contains all reviewer reports in order by version, followed by all author rebuttals in order by version.

Version 0:

Reviewer comments:

Reviewer #1

(Remarks to the Author)
In the manuscript entitled "DNA hypomethylation enables the transcriptional repressor SlSPL-CNR to control fruit flavor ester biosynthesis", Zeng and the coauthors performed systematic studies on SlSPL-CNR, a squamosa promoter binding protein-like transcriptional factor. DAP-seq analysis showed that SlSPL-CNR enriches within DNA containing -GTACGG-, -GTACTT-, or -GTACNA- motifs, and SLSPL-CNR preferentially binds to hypomethylated DNA. To revealed the underlying basis for target DNA preference, they solved the structure of SLSPL-CNR/DNA complex and performed structure-based modeling. ChIP-seq analysis showed that 90% peaks identified by ChIP-seq are overlapped with those of DAP-seq. They also identified one downstream target gene of SLSPL-CNR, SlAAT1. They proved that SLSPL-CNR binds to the demethylated protomer of SlAAT1, inhibiting its expression and the production of ester metabolites, which is critical for fruit flavor. Collectively, this work is interesting and the conclusions are well supported by the data provided. However, some issues need to be addressed to improve the manuscript.

1) In the manuscript, the authors also solved one crystal structure of DNA-complexed SLSPL-CNR. To confirm the quality of the structure, electron density maps should be presented, especially for the central -GTACGG- motif of the DNA and the DNA-interacting residues of SLSPL-CNR. The distances between DNA and the interacting residues should be provided in the main text or the e-l panels of Figure 4.
2) As depicted in Fig. 4l, the nucleobase of C4' of the complementary strand also forms base-specific interaction with SLSPL-CNR. Like C7, C4' could also be methylated. The impact of C4' methylation on binding to SLSPL-CNR should be detected.
3) Compared with Fig. 3a-b, 5mC methylation weakly inhibited DNA binding to SLSPL-CNR in Fig. 6e. How to explain this difference? For the line with 250x Cold-probe, why there is a lower band?
4) The motif enrichment data summarized in the b panel of supplementary Fig. 2 are confused. As the core motif_1 (46408) is also present in motif_3 (15327), motif_4 (14679), motif_10 (10831), motif_15 (5571), and serval other motifs. Besides, overlapping also occurs in motif_3 and motif_14, motif_10 and motif_30.

Reviewer #2

(Remarks to the Author)

The manuscript NCOMMS-25-099639 entitled "DNA hypomethylation enables the transcriptional repressor SlSPL-CNR to control fruit flavor ester biosynthesis" presents a quite detailed investigation into the epigenetic regulation of metabolite production during tomato fruit ripening. The authors demonstrate that ripening-associated DNA hypomethylation facilitates the binding of the SlSPL-CNR transcription factor to a GTACGG motif, leading to the transcriptional repression of SlAAT1. The study is technically robust, employing a comprehensive range of methodologies (DAP-seq, ampDAP-seq, WGBS, EMSA, ITC, ChIP-seq, crystal structural determination and analyses, dual luciferase assays, HS-SPME/GC-MS) etc. The manuscript is logically structured and clearly written.
While the work is solid and of interest to the plant biology community, its novelty is somewhat limited since its major results have already been known from animal studies and well expected conceptually. The general principle that DNA methylation status can modulate transcription factor binding affinity—either positively or negatively—is well-established. This study

effectively applies this established paradigm to a specific system (tomato ripening), without introducing major new mechanistic insights beyond this particular application, nevertheless, it is still deserved to be published on NC after some revision.

Major and Minor Points:
1. Structural Analysis (mainly related to Fig. 4): The structural model showing a clash between the methyl group of 5mC and residue Q94 is suggestive, it's best to demonstrate this point by showing some relevant electron density map. To strengthen this analysis, the authors should:
o Provide a parallel view/model illustrating the interaction between Q94 and a non-methylated cytosine at the same position (C7), clearly highlighting the position of Q94 within the overall protein-DNA interface.
o Include a discussion on the functional importance of residue Q94 for specific DNA base recognition. A static structural clash may be resolved through dynamic conformational adjustments. The significance of the observed clash hinges on whether Q94 is a critical residue for DNA binding. If it is non-essential, the functional impact of the clash may be minimal, weakening the proposed steric hindrance mechanism.
2. Experimental Validation of Q94 Role: The proposed central role of residue Q94 in conferring methylation sensitivity requires direct functional validation. A key experiment would be to mutate Q94 to Alanine (Q94A) and test the binding affinity of the mutant SlSPL-CNR protein to both methylated and non-methylated DNA probes via EMSA (or ITC). This would directly assess Q94's contribution to the observed methylation-sensitive binding. (Note: we could not locate the relevant data in Supplementary Fig. 4, which may require correction or clarification).

3. Supplementary Information: All Supplementary Figures are currently missing figure legends, which should be provided.

4. Mechanistic Clarification on the "Checkpoint" Model: The manuscript states that SlAAT1 expression increases during ripening yet is repressed by SlSPL-CNR, which is proposed to act as a "checkpoint." The model in the paper implies that SlDML2-mediated demethylation of the SlAAT1 promoter is a prerequisite for SlSPL-CNR binding and subsequent repression. This would be significantly strengthened by experimental evidence showing that preventing demethylation at the SlAAT1 promoter (e.g., via targeted mutation of SlDML2 or using methylation-stable promoter constructs in transgenic plants) leads to sustained SlAAT1 expression and altered ester profiles. Alternatively, more detailed work is needed to clarify the specific trigger or upstream signal that initiates the SlDML2-mediated demethylation at this specific locus, constituting the proposed "checkpoint."

5. The following reference about 10 years ago to address the general effects of cytosine methylation on general transcription factors binding to DNA should be cited. "Jin J., Lian T., Gu C., Yu K., Gao YQ, Su XD "The effects of cytosine methylation on general transcription factors" (2016) Sci Rep.; 6:29119. doi: 10.1038/srep29119".

Reviewer #3

(Remarks to the Author)
The authors employ an impressive array of integrated experimental approaches (DAP-seq/ampDAP-seq, ChIP-seq, WGBS, structural biology, CRISPR-Cas9 knockout, and metabolomics) to reveal a novel and significant regulatory mechanism: DNA hypomethylation facilitates the binding of the transcriptional repressor SlSPL-CNR, leading to the repression of its target gene SlAAT1 and the modulation of flavor-related ester biosynthesis in tomato fruit. This finding challenges the conventional view that DNA hypomethylation is universally associated with gene activation and provides an important paradigm for understanding the integration of epigenetic dynamics and metabolic control in plants. The study is generally well-designed, and the data are of high quality, with the structural mechanism and evidence for cross-species conservation being particularly compelling. However, the manuscript has several key weaknesses.
1. The authors have precisely identified Gln94 as the key residue for sensing cytosine methylation through structural studies and in vitro EMSA. However, the physiological function of this residue in plants remains entirely unvalidated. This is a critical gap. It is essential to perform a functional complementation assay in the slcnr CRISPR knockout background. Comparing plants expressing the wild-type SlSPL-CNR protein to those expressing a Gln94 point mutant (e.g., Q94A) for the following parameters would provide definitive proof: SlAAT1 transcript levels (RT-qPCR), in vivo binding of SlSPL-CNR to the SlAAT1 promoter (ChIP-qPCR), and accumulation of key ester volatiles (targeted GC-MS). Only if the wild-type protein, but not the Q94A mutant, rescues the mutant phenotype can Gln94 be conclusively proven as essential for methylation sensing and repressive function in vivo.
2. The chain of evidence linking SlSPL-CNR to the flavor ester phenotype is incomplete. While the metabolic phenotype is attributed largely to SlAAT1, the genome-wide ChIP-seq data likely contain binding information for other alcohol acyltransferase (AAT) genes. The authors should analyze whether SlSPL-CNR binds to promoters of other AAT family members and discuss their potential contribution. In addition, the link between the metabolomic data and "flavor" is weak. The study lacks analysis of established key tomato flavor esters (e.g., hexyl acetate) and sensory evaluation data. To strengthen this, targeted quantification of 2-3 key flavor esters should be added, and the discussion should clarify that the connection to perceptible flavor improvement is currently speculative.
3. The proposed model of SlSPL-CNR as a negative feedback point is not sufficiently reconciled with the observation that SlAAT1 expression still rises dramatically during ripening (Fig. 6c). This paradox suggests its repression is either overridden by stronger activators or is itself a fine-tuning mechanism. The discussion should be deepened by integrating known ripening activators (e.g., RIN, NOR) to propose a dynamic balancing model. For instance, early hypomethylation may allow SlSPL-CNR binding to prevent premature expression, while later, dominant activation mechanisms drive high SlAAT1 expression. This would make the model more biologically nuanced and convincing.

4. Although the study indicates that SlDML2 facilitates SlSPL-CNR binding and represses SlAAT1 expression through demethylation during fruit ripening, the causality of this process remains incompletely established. The authors propose that DNA demethylation promotes SlSPL-CNR binding and subsequently inhibits SlAAT1 expression; however, whether the experimental design can clearly distinguish the cause-and-effect relationship between demethylation and the repressive function of SlSPL-CNR remains questionable.
5. The study has shown that SlDML2 influences the binding of SlSPL-CNR through its demethylation activity. However, is there a direct protein-protein interaction between the two proteins?
6. In Discussion section, it would be beneficial to briefly discuss the relationship between this finding and the classic Cnr epiallele.

Reviewer #4

(Remarks to the Author)
I co-reviewed this manuscript with one of the reviewers who provided the listed reports. This is part of the Nature Communications initiative to facilitate training in peer review and to provide appropriate recognition for Early Career Researchers who co-review manuscripts.

Version 1:

Reviewer comments:

Reviewer #1

(Remarks to the Author)
The authors have addressed my concerns. No more questions.

Reviewer #2

(Remarks to the Author)
I am satisfied with the author's answers to my questions, and wish their best of luck for the publication with this paper.

Reviewer #3

(Remarks to the Author)
The author generally addressed my concerns.

Reviewer #4

(Remarks to the Author)
I co-reviewed this manuscript with one of the reviewers who provided the listed reports. This is part of the Nature Communications initiative to facilitate training in peer review and to provide appropriate recognition for Early Career Researchers who co-review manuscripts.

Open Access This Peer Review File is licensed under a Creative Commons Attribution 4.0 International License, which permits use, sharing, adaptation, distribution and reproduction in any medium or format, as long as you give appropriate credit to the original author(s) and the source, provide a link to the Creative Commons license, and indicate if changes were made.
In cases where reviewers are anonymous, credit should be given to 'Anonymous Referee' and the source.
The images or other third party material in this Peer Review File are included in the article's Creative Commons license, unless indicated otherwise in a credit line to the material. If material is not included in the article's Creative Commons license and your intended use is not permitted by statutory regulation or exceeds the permitted use, you will need to obtain permission directly from the copyright holder.
To view a copy of this license, visit https://creativecommons.org/licenses/by/4.0/

We sincerely appreciate the reviewers' constructive feedback and valuable recommendations. In response, we have conducted additional experiments and data analysis to address each concern raised. The manuscript has been comprehensively revised to incorporate these improvements. Below are our detailed replies (blue) to the reviewers' comments (black).

REVIEWER COMMENTS

Reviewer #1 (Remarks to the Author):

In the manuscript entitled "DNA hypomethylation enables the transcriptional repressor SlSPL-CNR to control fruit flavor ester biosynthesis", Zeng and the coauthors performed systematic studies on SlSPL-CNR, a squamosa promoter binding protein-like transcriptional factor. DAP-seq analysis showed that SlSPL-CNR enriches within DNA containing -GTACGG-, -GTACTT-, or -GTACNA- motifs, and SLSPL-CNR preferentially binds to hypomethylated DNA. To revealed the underlying basis for target DNA preference, they solved the structure of SLSPL-CNR/DNA complex and performed structure-based modeling. ChIP-seq analysis showed that 90% peaks identified by ChIP-seq are overlapped with those of DAP-seq. They also identified one downstream target gene of SLSPL-CNR, SlAAT1. They proved that SLSPL-CNR binds to the demethylated protomer of SlAAT1, inhibiting its expression and the production of ester metabolites, which is critical for fruit flavor. Collectively, this work is interesting and the conclusions are well supported by the data provided. However, some issues need to be addressed to improve the manuscript.

We thank the reviewer for the positive comments.

1) In the manuscript, the authors also solved one crystal structure of DNA-complexed SLSPL-CNR. To confirm the quality of the structure, electron density maps should be presented, especially for the central -GTACGG- motif of the DNA and the DNA-interacting residues of SLSPL-CNR. The distances between DNA and the interacting residues should be provided in the main text or the e-l panels of Figure 4.

Response: We thank the reviewer for this good suggestion. We have extensively revised our figure. The distances between DNA and the interacting residues are now marked in both the main Fig. 4 and the Supplementary Fig. 4.

2) As depicted in Fig. 4l, the nucleobase of C4' of the complementary strand also forms base-specific interaction with SLSPL-CNR. Like C7, C4' could also be methylated. The impact of C4' methylation on binding to SLSPL-CNR should be detected.

Response: We thank the reviewer for the suggestion. To address this concern, we designed a panel of site-specifically methylated GTAC-box1P DNA probes and performed comprehensive EMSA analyses to investigate the impact of C4' methylation on SlSPL-CNR binding. EMSA results demonstrate that both full-length SlSPL-CNR and its SBP domain (aa46-136) exhibit robust binding to the unmethylated GTAC-box1P probe Methylation of C7 completely abrogates this interaction, while C4' methylation (F0/R2 probes in Supplementary Fig. 6) didn't show

significant inhibitory effect on. SlSPL-CNR binding These experiments support that SlSPL-CNR binding is mainly affected by DNA methylation at the C7 position of the core 5’-GTACGG-3’ motif. We integrated these new results in the revised manuscript.as following. “Considering that C4' of complementary strand also forms base-specific interaction with SlSPL-CNR (Fig. 4d), we further investigated the impact of C4' methylation on SlSPL-CNR binding. EMSA results demonstrate that DNA probes with C4' methylation didn’t show significant inhibitory effect on SlSPL-CNR binding (Supplementary Fig. 6).” (Supplementary Fig. 6).

3) Compared with Fig. 3a-b, 5mC methylation weakly inhibited DNA binding to SLSPL-CNR in Fig. 6e. How to explain this difference? For the line with 250x Cold-probe, why there is a lower band?

Response: We thank the reviewer for this careful observation. We attribute the different inhibitory effects between Fig. 3a-b and Fig. 6e to differences in probe length and motif composition. In Fig. 3a-b, we employed a 15-bp short DNA probe containing a single core -GTACGG- motif, where 5-mC hemi-methylation completely abrogated SlSPL-CNR binding. In contrast, Fig. 6e utilized a 65-bp *SlAAT1* promoter probe encompassing two distinct GTAC-box sites (containing -GTACGG- and -GTACGA- motifs) with extended flanking sequences. While 5-mC modification reduced binding in this longer probe, the residual weak band likely results from the presence of the second motif with differential methylation sensitivity, together with potential non-specific interactions from the extended flanking sequences.

To resolve these confounding effects and dissect the site-specific methylation sensitivity, we rationally truncated the original 65-bp probe into two 20-bp short DNA probes, each encompassing a single core GTAC motif (designated PAAT1_GTAC-box1 and PAAT1_GTAC-box2, respectively) (Supplementary Fig. 11). EMSA results demonstrate that SlSPL-CNR binds both unmethylated core probes with high affinity, and these interactions are efficiently competed by a 500-fold excess of cold probe, confirming sequence-specific binding. Upon hemi-methylation of the antisense strand, SlSPL-CNR displays pronounced site-selective sensitivity: binding to hmPAAT1_GTAC-box1 (containing GTACGG) is completely abolished, identical to the observation in Fig. 3, whereas binding to hmPAAT1_GTAC-box2 (containing GTACGA) is only partially reduced. This indicates that the -GTACGA- motif exhibits intrinsically weaker sensitivity to methylation inhibition compared to the -GTACGG- motif. Collectively, the weaker inhibitory effect observed in Fig. 6e reflects the composite behavior of the two distinct GTAC-box sites: while the GTACGG site (box1) is fully inhibited by methylation, the GTACGA site (box2) retains partial binding capacity, resulting in an overall weaker but detectable residual complex. We added this results in the revised manuscript as following “Given that the inhibitory effect of 5-mC in Fig. 6e was less pronounced than the complete abolition observed with the short probe in Fig. 3a-b, and considering that the 65-bp *ProSlAAT1* probe contains two distinct GTAC motifs (GTACGG and GTACGA), we truncated it into two 20-bp fragments (PAAT1_GTAC-box1 and PAAT1_GTAC-box2) to assess motif-specific

sensitivity (Supplementary Fig. 11). EMSA revealed that hemi-methylation completely blocked binding to the GTACGG-containing probe (consistent with Fig. 3b), whereas it only partially reduced binding to the GTACGA-containing probe, indicating that the differential methylation sensitivity of these two motifs accounts for the weaker inhibition observed in Fig. 6e." (Supplementary Fig. 11).

For the 250x Cold probe (the fifth lane) in Fig. 6e, we observed that the protein-probe complex band corresponding to the normal shift (as in the third lane) almost entirely forms a smear lower band, as noted by the reviewer. We think this may reflect that the correct binding between protein and probe, as shown by our crystal structure, was disrupted. Instead, the protein and probe form an unusual complex that is much less stable due to non-specific binding, as the protein is enriched in positively charged amino acids. In agreement with our hypothesis, the additional Cold probe to 500x further interferes the unstable complex formation to fully disrupt the complex to a band like the fee probe.

4) The motif enrichment data summarized in the b panel of supplementary Fig. 2 are confused. As the core motif_1 (46408) is also present in motif_3 (15327), motif_4 (14679), motif_10 (10831), motif_15 (5571), and serval other motifs. Besides, overlapping also occurs in motif_3 and motif_14, motif_10 and motif_30.

Response: We sincerely thank the reviewer for their careful and insightful observation regarding the motif enrichment data. The reviewer is completely correct in noticing that core motif_1 is present within other motifs, and that its total occurrence count (46,408) aggregates its sub-motifs (motifs 3, 4, 10, and 15) (Supplementary Fig. 2b). We also agree that similar overlaps exist between motif_3 and motif_14, as well as motif_10 and motif_30.

To address the reviewer's concern, we would like to clarify that the MEME-ChIP combined with FIMO pipeline represents the current mainstream standard for de novo motif discovery and genome-wide scanning. However, an inherent characteristic of this algorithm is that it evaluates each Position Weight Matrix independently across the genome, which inevitably produces overlapping motif occurrences that are not mutually exclusive. This is not a technical limitation, but rather the expected algorithmic behavior, as systematically demonstrated by ENCODE project standards[1]. When a shorter "core" motif and its longer, nested sub-motif variants are scanned independently, they will match at the same genomic loci, resulting in mathematically overlapping counts. Biologically, we intentionally retained these unmerged variants to preserve binding resolution. While sub-motifs share an identical core sequence, their distinct flanking bases confer specific binding affinities and epigenetic sensitivities, as established by foundational DAP-seq studies[2] and recent TF complex analyses[3]. Artificially merging these variants would sacrifice this crucial biological resolution. Our approach aligns with current field standards, as evidenced by recent top-tier publications[4]. The overlapping motif counts accurately reflect the standard outputs of this established methodology.

To prevent confusion, we have added the following clarification in the "Methods" section: "Note that motif scanning with FIMO evaluates each motif independently, resulting in overlapping occurrences between core motifs and their nested sub-motif variants. These overlaps are an inherent feature of the algorithm and were intentionally retained unmerged to preserve biological resolution of flanking sequence preferences."

Reviewer #2 (Remarks to the Author):

The manuscript NCOMMS-25-099639 entitled "DNA hypomethylation enables the transcriptional repressor SlSPL-CNR to control fruit flavor ester biosynthesis" presents a quite detailed investigation into the epigenetic regulation of metabolite production during tomato fruit ripening. The authors demonstrate that ripening-associated DNA hypomethylation facilitates the binding of the SlSPL-CNR transcription factor to a GTACGG motif, leading to the transcriptional repression of SlAAT1. The study is technically robust, employing a comprehensive range of methodologies (DAP-seq, ampDAP-seq, WGBS, EMSA, ITC, ChIP-seq, crystal structural determination and analyses, dual luciferase assays, HS-SPME/GC-MS) etc. The manuscript is logically structured and clearly written.

While the work is solid and of interest to the plant biology community, its novelty is somewhat limited since its major results have already been known from animal studies and well expected conceptually. The general principle that DNA methylation status can modulate transcription factor binding affinity—either positively or negatively—is well-established. This study effectively applies this established paradigm to a specific system (tomato ripening), without introducing major new mechanistic insights beyond this particular application, nevertheless, it is still deserved to be published on NC after some revision.

We thank the reviewer for the supportive comments.

Major and Minor Points:

1. Structural Analysis (mainly related to Fig. 4): The structural model showing a clash between the methyl group of 5mC and residue Q94 is suggestive, it's best to demonstrate this point by showing some relevant electron density map. To strengthen this analysis, the authors should:

o Provide a parallel view/model illustrating the interaction between Q94 and a non-methylated cytosine at the same position (C7), clearly highlighting the position of Q94 within the overall protein-DNA interface.

Response: We thank the reviewer for this good suggestion. We have extensively revised our figure by supplementing the electron density maps of the interacting residues. A parallel view of the interaction between Q94 and a non-methylated C7 is shown in Fig. 4k, and also highlighted in Supplementary Fig. 4 to show the Q94 position within the overall protein-DNA interface in two orientations.

o Include a discussion on the functional importance of residue Q94 for specific DNA base recognition. A static structural clash may be resolved through dynamic conformational adjustments. The significance of the observed clash hinges on whether Q94 is a critical residue for DNA binding. If it is non-essential, the functional impact of the clash may be minimal, weakening the proposed steric hindrance mechanism.

Response: Thank you for this important suggestion. The two hydrogen bonds between C7 base and Q94/Q95 ***lock*** the relative conformation of the C7 base and Q94 main chain carbonyl group (Supplementary Fig. 4a). We have added a short

discuss in the main text as suggested: "Consequently, both the C7 base and the Q94 main chain cannot move or rotate, and the methylation of C7 will undoubtedly introduce steric conflict between the methyl group and the Q94 main-chain carbonyl as show in Fig. 4n, explaining the unmethylated DNA preference for the CNR SBP domain." we have integrated these results into the revised manuscript.

2. Experimental Validation of Q94 Role: The proposed central role of residue Q94 in conferring methylation sensitivity requires direct functional validation. A key experiment would be to mutate Q94 to Alanine (Q94A) and test the binding affinity of the mutant SlSPL-CNR protein to both methylated and non-methylated DNA probes via EMSA (or ITC). This would directly assess Q94's contribution to the observed methylation-sensitive binding. (Note: we could not locate the relevant data in Supplementary Fig. 4, which may require correction or clarification).

Response: We thank the reviewer for this important suggestion. We apologize for the confusion regarding the location of Q94 mutagenesis data. In the revised manuscript, we included the EMSA results of Q94A mutant data in Supplementary Fig. 5. and described these results in the main text as following "Further analysis by EMSA revealed that site-directed mutagenesis substituting Gln to Ala at Gln94 (Q94A) or Gln95 (Q95A) in SlSPL-CNR weakened its binding to target GTAC-box1 (Supplementary Fig. 5). It is noteworthy that, in comparison to single-point mutations, the double mutation (Q94/95A) at positions 94 and 95 exhibits a significantly stronger inhibitory effect on DNA binding affinity. This reveals that Q94/95 are two key amino acid residues in determining the binding affinity to the GTACGG cis-element (Supplementary Fig. 5)."

3. Supplementary Information: All Supplementary Figures are currently missing figure legends, which should be provided.

Response: In the revised manuscript, we provided figure legends for all Supplementary Figures in the "Supplementary Information" document.

4. Mechanistic Clarification on the "Checkpoint" Model: The manuscript states that SlAAT1 expression increases during ripening yet is repressed by SlSPL-CNR, which is proposed to act as a "checkpoint." The model in the paper implies that SlDML2-mediated demethylation of the SlAAT1 promoter is a prerequisite for SlSPL-CNR binding and subsequent repression. This would be significantly strengthened by experimental evidence showing that preventing demethylation at the SlAAT1 promoter (e.g., via targeted mutation of SlDML2 or using methylation-stable promoter constructs in transgenic plants) leads to sustained SlAAT1 expression and altered ester profiles. Alternatively, more detailed work is needed to clarify the specific trigger or upstream signal that initiates the SlDML2-mediated demethylation at this specific locus, constituting the proposed "checkpoint."

Response: We appreciate the reviewer's excellent suggestion regarding precise targeted demethylation experiments. Indeed, site-specific epigenetic editing (e.g., targeting SlDML2 activity or using methylation-stable promoter constructs) would

provide the most direct evidence to establish causality between promoter demethylation and SlSPL-CNR binding. However, precise epigenetic editing tools (such as targeted demethylation using dCas9-TET1 or similar systems) remain technically extremely challenging in dicot crops like tomato and are currently not well-established in the plant research community worldwide. Consequently, this gold standard experiment was not technically feasible for the current revision.

To address the reviewer's core concern regarding whether SlDML2-mediated demethylation is required for SlSPL-CNR binding at the *SlAAT1* promoter, we have instead incorporated alternative experiments and data analyses that collectively support our proposed model:

1. Comparative methylome analysis: We analyzed DNA methylome data from wild-type and *dml2* mutant fruits and found that the demethylation at the CNR-binding motif within the *SlAAT1* promoter is completely blocked in the *dml2* mutant (Supplementary Fig. 15b). This demonstrates that SlDML2 is responsible for the observed epigenetic changes at this locus.

2. Direct occupancy evidence: We performed ChIP-qPCR using the *pDML2::gDML2-3xFLAG* transgenic line, which revealed direct occupancy of SlDML2 at the *SlAAT1* promoter during ripening (Supplementary Fig. 15c).

Together with our existing SlSPL-CNR ChIP-seq data (Fig. 6d) and EMSA results (Fig. 6e, Supplementary Fig. 11) showing that DNA methylation impedes SlSPL-CNR binding both *in vivo* and *in vitro*, these new data strongly support our model that SlDML2-dependent demethylation is a prerequisite for SlSPL-CNR binding and subsequent transcriptional regulation of *SlAAT1*.

Regarding the reviewer's suggestion to clarify the specific trigger or upstream signal that initiates SlDML2-mediated demethylation at the *SlAAT1* locus: We appreciate this valuable insight. The current study focuses specifically on the methylation-sensitive DNA-binding activity of SlSPL-CNR and the SlDML2-dependent epigenetic regulation of CNR binding at flavor-related genes. The precise mechanisms governing how DNA demethylases are recruited to specific genomic loci remain poorly understood in both plants and animals and represent a significant outstanding question in the field. While investigating the upstream signals that target SlDML2 to the *SlAAT1* promoter is beyond the scope of the present study, we agree that elucidating the precise targeting mechanisms of demethylases is an important direction for future research, and we hope to pursue this question in subsequent studies.

5. The following reference about 10 years ago to address the general effects of cytosine methylation on general transcription factors binding to DNA should be cited. "Jin J., Lian T., Gu C., Yu K., Gao YQ, Su XD "The effects of cytosine methylation on general transcription factors" (2016) Sci Rep.; 6:29119. doi: 10.1038/srep29119".

Response: We thank the reviewer for bringing this important reference to our attention. In the revised manuscript, we have cited it in the Introduction, following the sentence: “DNA methylation can directly or indirectly alter the binding affinity between transcription factors and their target DNA sequences.”

Reviewer #3 (Remarks to the Author):

The authors employ an impressive array of integrated experimental approaches (DAP-seq/ampDAP-seq, ChIP-seq, WGBS, structural biology, CRISPR-Cas9 knockout, and metabolomics) to reveal a novel and significant regulatory mechanism: DNA hypomethylation facilitates the binding of the transcriptional repressor SlSPL-CNR, leading to the repression of its target gene SlAAT1 and the modulation of flavor-related ester biosynthesis in tomato fruit. This finding challenges the conventional view that DNA hypomethylation is universally associated with gene activation and provides an important paradigm for understanding the integration of epigenetic dynamics and metabolic control in plants. The study is generally well-designed, and the data are of high quality, with the structural mechanism and evidence for cross-species conservation being particularly compelling. However, the manuscript has several key weaknesses.

We thank the reviewer for the supportive comments and constructive suggestions.

1. The authors have precisely identified Gln94 as the key residue for sensing cytosine methylation through structural studies and in vitro EMSA. However, the physiological function of this residue in plants remains entirely unvalidated. This is a critical gap. It is essential to perform a functional complementation assay in the slcnr CRISPR knockout background. Comparing plants expressing the wild-type SlSPL-CNR protein to those expressing a Gln94 point mutant (e.g., Q94A) for the following parameters would provide definitive proof: SlAAT1 transcript levels (RT-qPCR), in vivo binding of SlSPL-CNR to the SlAAT1 promoter (ChIP-qPCR), and accumulation of key ester volatiles (targeted GC-MS). Only if the wild-type protein, but not the Q94A mutant, rescues the mutant phenotype can Gln94 be conclusively proven as essential for methylation sensing and repressive function in vivo.

Response: We appreciate the reviewer's suggestion for functional complementation in the *slcnr* knockout background to study the physiological function of Gln94. Due to the 4-6 month timeline required to generate transgenic tomato fruits, we were unable to complete stable transformation experiments during the revision period. Instead, we performed dual-luciferase transient expression assays to validate the functional importance of Gln94 in planta (Supplementary Fig. 12b). The result demonstrates that the SlSPL-CNR fragment (aa46-136) significantly represses the activity of the *SlAAT1* promoter (****$p<0.0001$ vs. control). Importantly, the Q94A mutation substantially attenuates this inhibitory effect (***$p<0.001$), indicating that Gln94 is a critical residue for SlSPL-CNR-mediated transcriptional regulation of *SlAAT1*. In addition to these in planta assays, we also performed *in vitro* EMSA analysis (Supplementary Fig. 12a) to further examine the molecular mechanism. The Q94A mutant protein completely loses DNA-binding ability to both PAAT1_GTAC-box1 and PAAT1_GTAC-box2 probes, demonstrating that Gln94 is essential for SlSPL-CNR–DNA interaction. Together, these in planta and *in vitro* results establish that Gln94 is critical for both DNA binding and the repressive function of SlSPL-CNR. We acknowledge that stable complementation in tomato fruits remains an important goal for future studies to fully validate these findings in the fruit-specific context.

2. The chain of evidence linking SlSPL-CNR to the flavor ester phenotype is incomplete. While the metabolic phenotype is attributed largely to SlAAT1, the genome-wide ChIP-seq data likely contain binding information for other alcohol acyltransferase (AAT) genes. The authors should analyze whether SlSPL-CNR binds to promoters of other AAT family members and discuss their potential contribution. In addition, the link between the metabolomic data and "flavor" is weak. The study lacks analysis of established key tomato flavor esters (e.g., hexyl acetate) and sensory evaluation data. To strengthen this, targeted quantification of 2-3 key flavor esters should be added, and the discussion should clarify that the connection to perceptible flavor improvement is currently speculative.

Response: We sincerely appreciate the reviewer's constructive suggestions. We have now thoroughly addressed this concern by analyzing both the transcript abundance of all five annotated *SlAAT* family genes and the genome-wide binding profile of SlSPL-CNR. As previously reported, tomato contains five annotated alcohol acyltransferase genes (*SlAAT1*–SlAAT5)[5]. Our RNA-seq data clearly demonstrate that *SlAAT1* (FPKM > 800) is the only family member exhibiting substantial expression in fruit tissues during ripening. In striking contrast, *SlAAT2*–*SlAAT5* show minimal to negligible expression throughout all ripening stages (all FPKM < 8) (Supplementary Fig. 10a). This expression pattern establishes *SlAAT1* as the overwhelmingly dominant contributor to ester biosynthesis in ripening tomato fruit, which is fully aligns with previous functional studies identifying *SlAAT1* as the major ripening-associated acyltransferase responsible for volatile ester production in tomato[5].

Following the reviewer's suggestion, we also re-examined our genome-wide ChIP-seq data for SlSPL-CNR binding at the promoters of all *SlAAT* family members. No significant enrichment of SlSPL-CNR binding was detected at the promoter regions of *SlAAT2, SlAAT3, SlAAT4,* or *SlAAT5* in CNR-BrChIP (Supplementary Fig. 10b). This binding specificity, combined with the exclusive fruit-expression pattern of *SlAAT1*, strongly supports a model where SlSPL-CNR selectively represses the dominant ripening-associated acyltransferase to regulate ester volatile production, rather than coordinating the broader *AAT* gene family.

In addition, the reviewer suggested to detect key tomato flavor esters (e.g., hexyl acetate). Prior quantitative analyses of flavor-relevant esters were conducted almost exclusively at the fully ripe stage when these compounds reach peak concentrations[5,6,7]. However, our volatile profiling was conducted solely at the breaker stage, during which key flavor esters (e.g., hexyl acetate) were almost undetectable (Supplementary Data 8). We fully agree with the reviewer that the connection between our metabolomic findings and perceptible flavor remains putative. Our analysis was conducted at the breaker stage rather than the flavor-relevant ripe stage, and sensory evaluation data are not included in this study. Accordingly, we have explicitly clarified this limitation and moderated our claims throughout the revised Discussion as following:

"Although our volatile profiling was conducted at the breaker stage rather than at the flavor-relevant ripe stage and sensory evaluation data were not included, the observed upregulation of *SlAAT1* and the concomitant elevation of multiple ester species suggest an enhanced ester biosynthetic capacity in *slcnr-2*. Accordingly, the connection between these metabolic changes and perceptible flavor improvement should be considered putative, and further sensory validation at the fully ripe stage will be required to substantiate this link."

3. The proposed model of SlSPL-CNR as a negative feedback point is not sufficiently reconciled with the observation that SlAAT1 expression still rises dramatically during ripening (Fig. 6c). This paradox suggests its repression is either overridden by stronger activators or is itself a fine-tuning mechanism. The discussion should be deepened by integrating known ripening activators (e.g., RIN, NOR) to propose a dynamic balancing model. For instance, early hypomethylation may allow SlSPL-CNR binding to prevent premature expression, while later, dominant activation mechanisms drive high SlAAT1 expression. This would make the model more biologically nuanced and convincing.

Response: We sincerely appreciate the reviewer's insightful suggestion to refine our regulatory model. We have revised the discussion in the main text to propose a time-resolved regulatory model: "While SlSPL-CNR binds the *SlAAT1* promoter to suppress its expression, the overall upregulation of *SlAAT1* during ripening is consistent with established activation by NOR[8]. This suggests that SlSPL-CNR functions not to abolish *SlAAT1* expression, but to attenuate its overexpression, serving as a developmental checkpoint that prevents premature ester accumulation while permitting ripening-induced accumulation (Supplementary Fig. 18)." This provides a clear explanation for how repression and activation coexist during ripening.

4. Although the study indicates that SlDML2 facilitates SlSPL-CNR binding and represses SlAAT1 expression through demethylation during fruit ripening, the causality of this process remains incompletely established. The authors propose that DNA demethylation promotes SlSPL-CNR binding and subsequently inhibits SlAAT1 expression; however, whether the experimental design can clearly distinguish the cause-and-effect relationship between demethylation and the repressive function of SlSPL-CNR remains questionable.

Response: We agree with the reviewer that in our previous manuscript, although we showed DNA methylation at *SlAAT1* is decreased during ripening, we did not demonstrate whether and how DML2 is involved in this epigenetic regulation. First, we analyzed DNA methylome data of wild type and *dml2* mutant, and demonstrated that the demethylation at SlSPL-CNR binding motif is totally blocked in *dml2* mutant, suggesting that DML2-mediated demethylation is responsible for the epigenetic change at *SlAAT1* promoter.

In DNA demethylation research area, the precise targeting of DNA demethylase is an important question that has not been well answered. Our previous study identified the

first protein complex (IDM-MBD complex) that recruits DNA demethylase in Arabidopsis[9]. However, in tomato, the targeting mechanism has not been studied. In this study, we investigated whether SlSPL-CNR binding could secondarily influence DML2-dependent DNA hypermethylation patterns across the *SlAAT1* promoter. We conducted additional WGBS for *slcnr* mutant, and the comparative analysis revealed that the methylation levels are similar between *slcnr* mutant and wild type, suggesting that deficiency does not trigger epigenetic remodeling at these genomic loci and that DML2-dependent DNA methylation works upstream of SlSPL-CNR (Supplementary Fig. 16).

5. The study has shown that SlDML2 influences the binding of SlSPL-CNR through its demethylation activity. However, is there a direct protein-protein interaction between the two proteins?

Response: We sincerely appreciate the reviewer's insightful question regarding potential protein–protein interactions between SlSPL-CNR and SlDML2. To address this point, we first performed AlphaFold3-based protein interaction prediction (Supplementary Fig. 17a). The low predicted interface template modeling (ipTM) score (0.24), indicates that no stable, high-confidence physical interaction forms between SlSPL-CNR and SlDML2. We then performed Y2H assays to test direct interactions between CNR-BD and truncated protein DML2 (aa1–1000)-AD—to avoid auto-activation of full DML2 (Supplementary Fig. 17b). No interaction signal was observed across tested combinations. Neither approach yielded detectable interaction signals, supporting the conclusion that SlSPL-CNR and SlDML2 do not engage in a stable, direct protein–protein interaction (Supplementary Fig. 17a, b). We added these new results in the revised manuscript.

6. In Discussion section, it would be beneficial to briefly discuss the relationship between this finding and the classic Cnr epiallele.

Response: We sincerely appreciate the reviewer's helpful suggestion to connect our findings to the classic *Cnr* epiallele. We have incorporated a brief discussion of this relationship into the revised Discussion section, as follows: "Previous studies have established the *Cnr* epiallele—defined by hypermethylation within the *SlSPL-CNR* promoter—as a paradigmatic example of epigenetic regulation governing tomato fruit ripening. Intriguingly, loss-of-function mutations in *SlSPL-CNR* do not fully recapitulate the severe ripening defects characteristic of the *Cnr* epiallele, implying that *SlSPL-CNR* deficiency alone cannot account for the complete molecular etiology of the *Cnr* phenotype. Our findings reveal that the conservation of methylation sensitivity across the SBP domain suggests that other tomato SPL family members may act redundantly to mitigate methylation-mediated transcriptional repression in the *Cnr* epiallelic background. Future investigations into the functional redundancy among SlSPL family members within the hypermethylated context of the *Cnr* epiallele will be critical to fully elucidate this regulatory cascade and hold significant value for dissecting the epigenetic control of fruit ripening." This addition provides a potential link between our mechanistic findings and the classic *Cnr* epiallele, offering a

possibility for the phenotypic discrepancy between *SlSPL-CNR* loss-of-function mutants and the *Cnr* epiallele.

Reviewer #4 (Remarks to the Author):

I co-reviewed this manuscript with one of the reviewers who provided the listed reports. This is part of the Nature Communications initiative to facilitate training in peer review and to provide appropriate recognition for Early Career Researchers who co-review manuscripts.

Response: We thank the reviewer.

Reference:

1. Kheradpour P, Kellis M. Systematic discovery and characterization of regulatory motifs in ENCODE TF binding experiments. *Nucleic Acids Res* **42**, 2976-2987 (2014).
2. O'Malley RC*, et al.* Cistrome and Epicistrome Features Shape the Regulatory DNA Landscape. *Cell* **165**, 1280-1292 (2016).
3. Li M*, et al.* Double DAP-seq uncovered synergistic DNA binding of interacting bZIP transcription factors. *Nat Commun* **14**, 2600 (2023).
4. Jiao W*, et al.* Transcriptional regulatory network reveals key transcription factors for regulating agronomic traits in soybean. *Genome Biol* **25**, 313 (2024).
5. Goulet C*, et al.* Divergence in the enzymatic activities of a tomato and Solanum pennellii alcohol acyltransferase impacts fruit volatile ester composition. *Mol Plant* **8**, 153-162 (2015).
6. Goulet C, Mageroy MH, Lam NB, Floystad A, Tieman DM, Klee HJ. Role of an esterase in flavor volatile variation within the tomato clade. *Proc Natl Acad Sci U S A* **109**, 19009-19014 (2012).
7. Zhang Z*, et al.* Volatilomics-Based Discovery of Key Volatiles Affecting Flavor Quality in Tomato. *Foods* **13**, (2024).
8. Cao X*, et al.* Transcriptional and epigenetic analysis reveals that NAC transcription factors regulate fruit flavor ester biosynthesis. *Plant J* **106**, 785-800 (2021).
9. Lang Z*, et al.* The methyl-CpG-binding protein MBD7 facilitates active DNA demethylation to limit DNA hyper-methylation and transcriptional gene silencing. *Mol Cell* **57**, 971-983 (2015).
