# Supplementary material for: DNA hypomethylation enables the transcriptional repressor SlSPL-CNR to control fruit flavor ester biosynthesis
Source: Nat Commun. 2026 Jul 1;17:8168. doi: 10.1038/s41467-026-75181-8 (PMC13458263; doi:10.1038/s41467-026-75181-8)
Supplement: Supplementary file 3 [file 41467_2026_75181_MOESM1_ESM.pdf]

Supplementary Information for

DNA hypomethylation enables the transcriptional repressor SlSPL-CNR to control fruit flavor ester biosynthesis

Zhifeng Zeng[1,2,3,#], Yu Ma[1,#], Hang He[2,3,#], Linzhu Li[2,3], Yuan Peng[2,3], Yawen Hou[1], Songge Chai[4], Pengcheng Wang[1], Cheng-Guo Duan[2,3], Jian-Kang Zhu[1], Jiamu Du[4,*], Zhaobo Lang[1,*]

[1]Institute of Advanced Biotechnology and School of Medicine, Southern University of Science and Technology, Shenzhen 518055, China
[2]Shanghai Center for Plant Stress Biology and CAS Center for Excellence in Molecular Plant Sciences, Chinese Academy of Sciences, Shanghai 200032, China
[3]University of Chinese Academy of Sciences, Beijing 100049, China
[4]Shenzhen Key Laboratory of Plant Genetic Engineering and Molecular Design, Institute of Plant and Food Science and Institute for Biological Electron Microscopy, Department of Biology, School of Life Sciences, Southern University of Science and Technology, Shenzhen 518055, China

# contributed equally to this work
*Correspondence: Zhaobo Lang (langzb@sustech.edu.cn), Jiamu Du (dujm@sustech.edu.cn)

This file includes:
Supplementary Fig. 1 to Supplementary Fig. 19

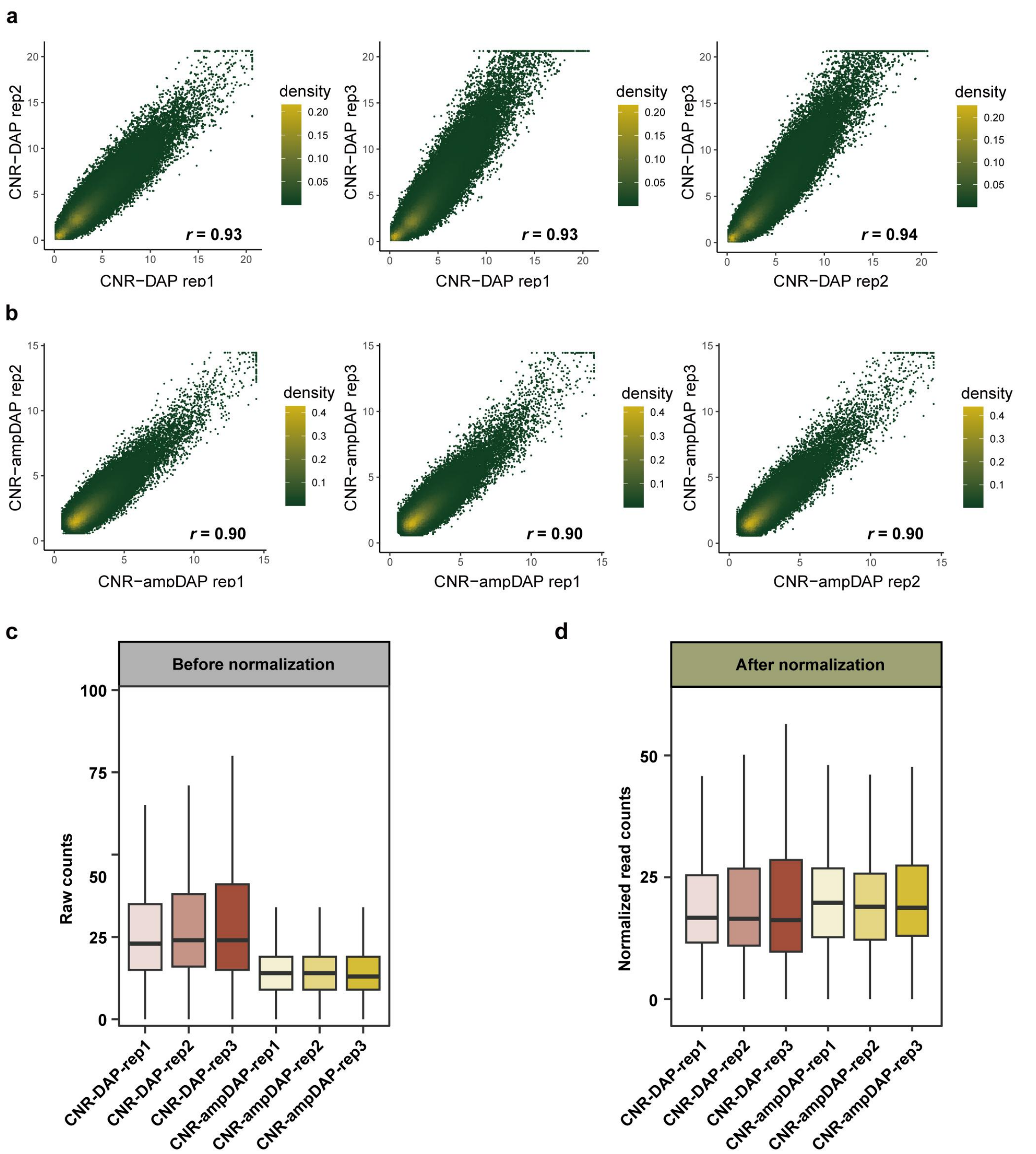

Supplementary Fig. 1 Replicate consistency and read count normalization analysis for CNR-DAP and CNR-ampDAP, related to Fig. 1. (a, b) Pairwise Pearson correlation coefficients (*r*) among three biological replicates of CNR-DAP **(a)** and CNR-ampDAP **(b)**. All *r* values exceed 0.9, indicating strong reproducibility. **(c, d)** Box plots showing read count distributions before **(c)** and after **(d)** normalization. Normalization was performed under the assumption that binding levels at non-methylated regions (5-mC $\leq$ 0.1) remain consistent across the two systems.

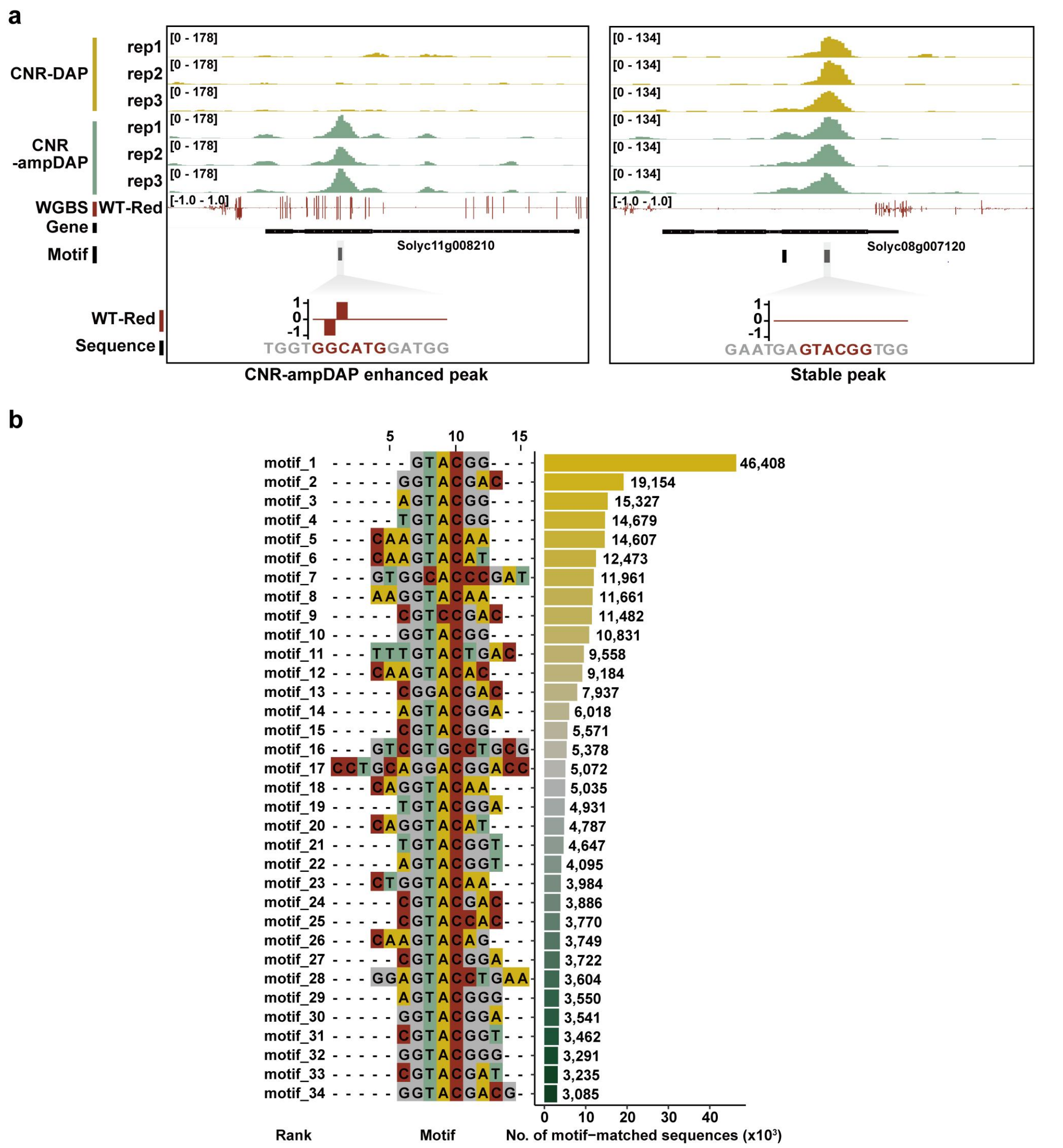

Supplementary Fig. 2 Representative genomic loci in two systems and motif enrichment for CNR-DAP, related to Fig. 2. a IGV tracks depicting SlSPL-CNR binding signals and corresponding DNA methylation levels at selected genomic loci. **b** Partial ranking of the most enriched TF binding motifs based on the number of motif-matched sequences. *De novo* identification of binding motifs was performed based on normalized CNR-DAP peaks.

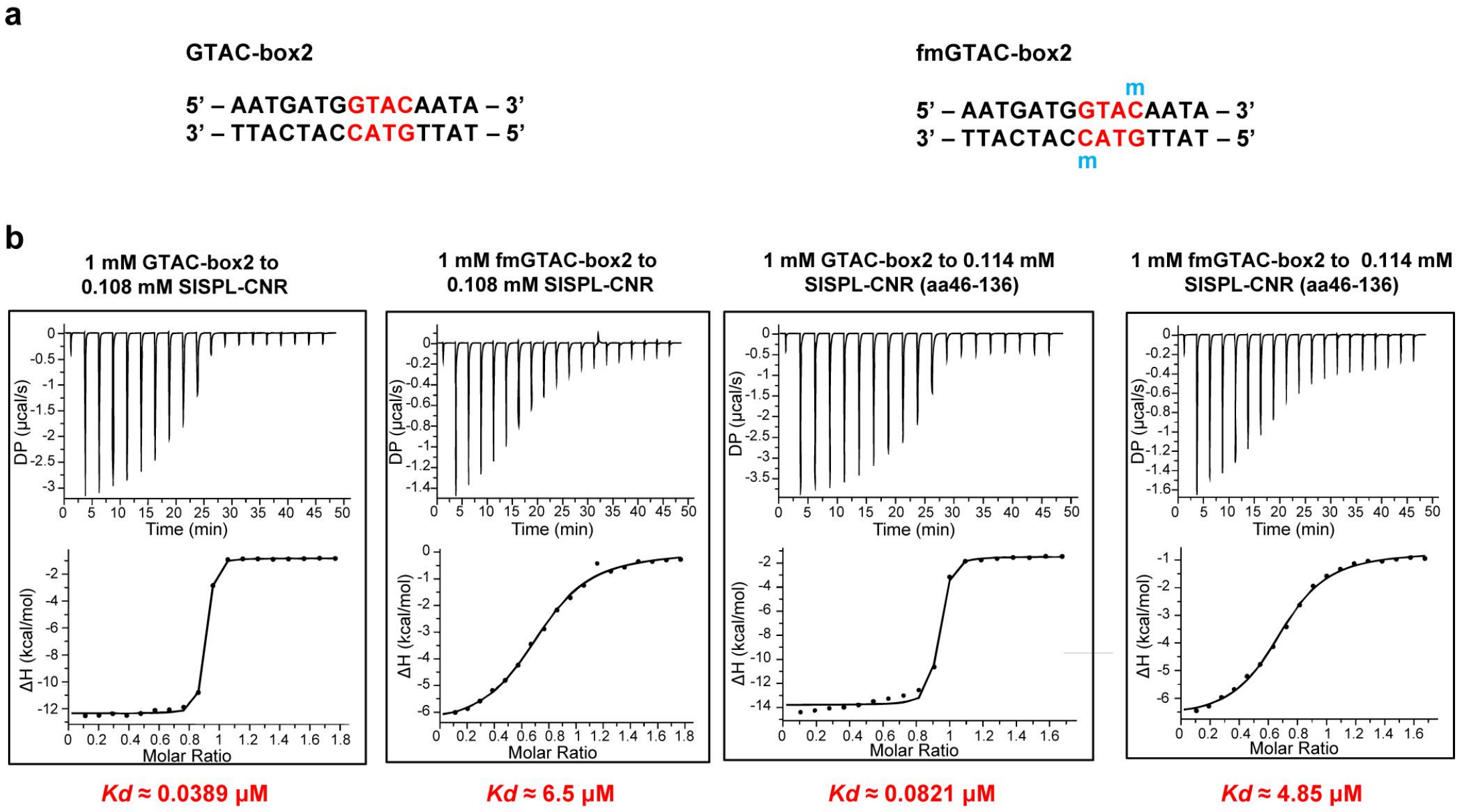

Supplementary Fig. 3 Binding affinity of SlSPL-CNR to GTAC-box2 with or without 5-mCs, related to Fig. 3. a DNA duplexes of GTAC-box2 (unmethylated at the GTAC motif; predicted binding site from CNR-DAP) and fmGTAC-box2 (fully methylated on both strands) were used in ITC experiments; lowercase "m" (blue) denotes 5-mC. GTAC-box2 (SL4.0ch05: 39,753,488-39,753,561) was identified as a SlSPL-CNR target by DAP-seq. **b** ITC analysis of full-length SlSPL-CNR and its SBP domain (aa 46–136) binding to unmethylated and fully methylated GTAC-box2 probes.

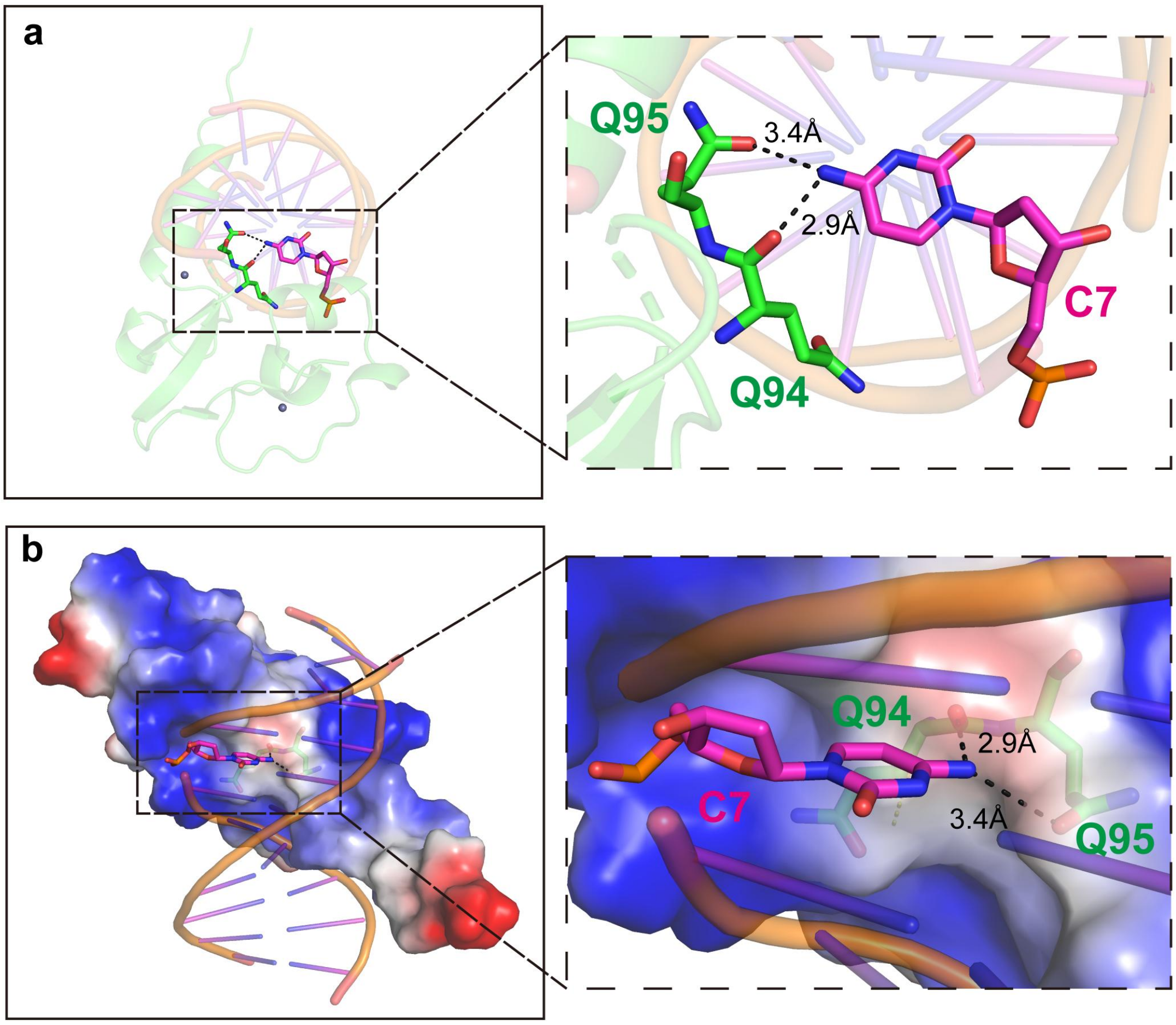

Supplementary Fig. 4 Highlighting the position of Gln94/Gln95 within the overall protein–DNA interface, related to Fig. 4. **a** The interaction between Gln94/Gln95 (Q94/Q95) and a non-methylated cytosine C7. The interacting residues and hydrogen bonds are highlighted by sticks and dashed black lines, respectively. **b** The electrostatic surface view shows the position of Q94/Q95 and cytosine C7.

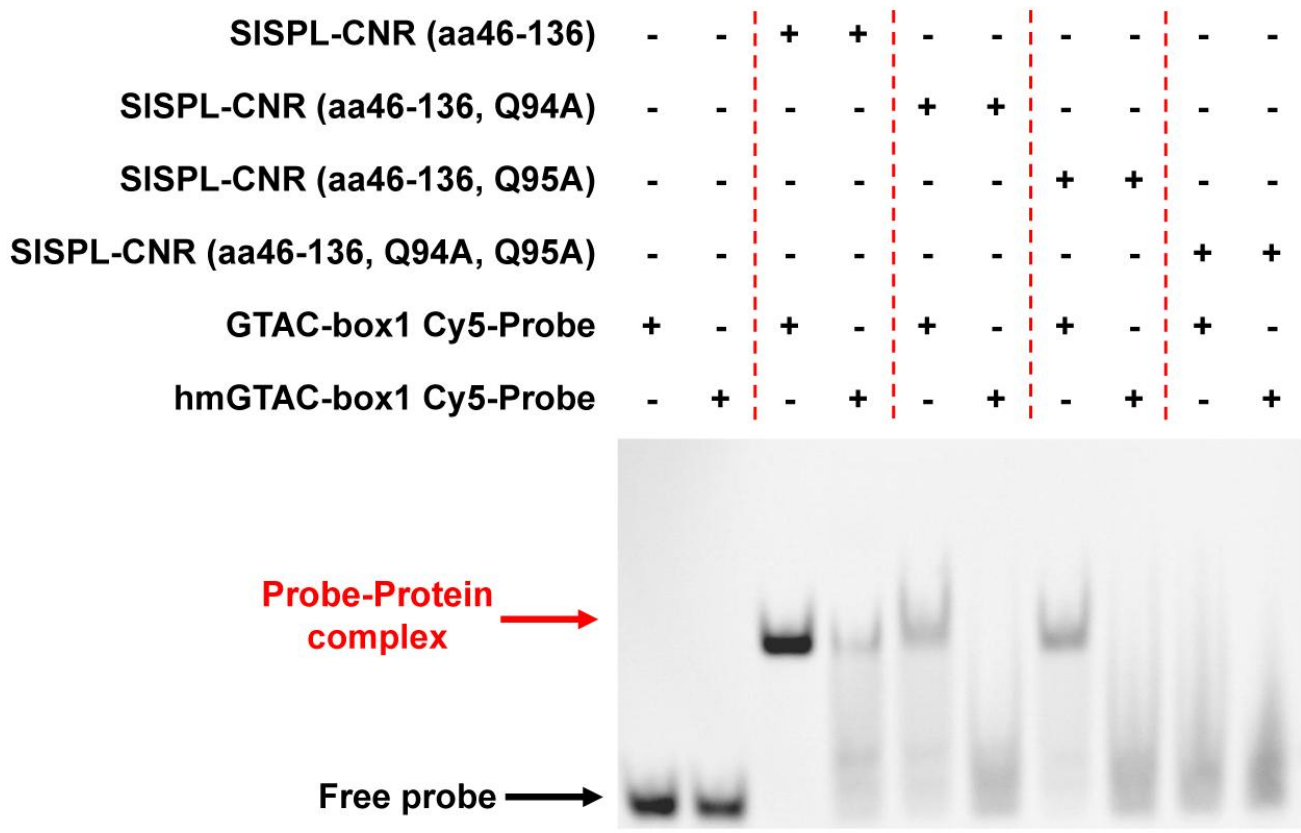

Supplementary Fig. 5 Site-directed mutagenesis of critical residues in the SlSPL-CNR SBP domain attenuates DNA-binding affinity, related to Fig. 4. EMSA validation of alanine substitutions at Gln94 (Q94A) and/or Gln95 (Q95A) within SlSPL-CNR (aa46–136) binding to DNA probes with or without 5-mC modification. Experiments were repeated independently three times with similar results.

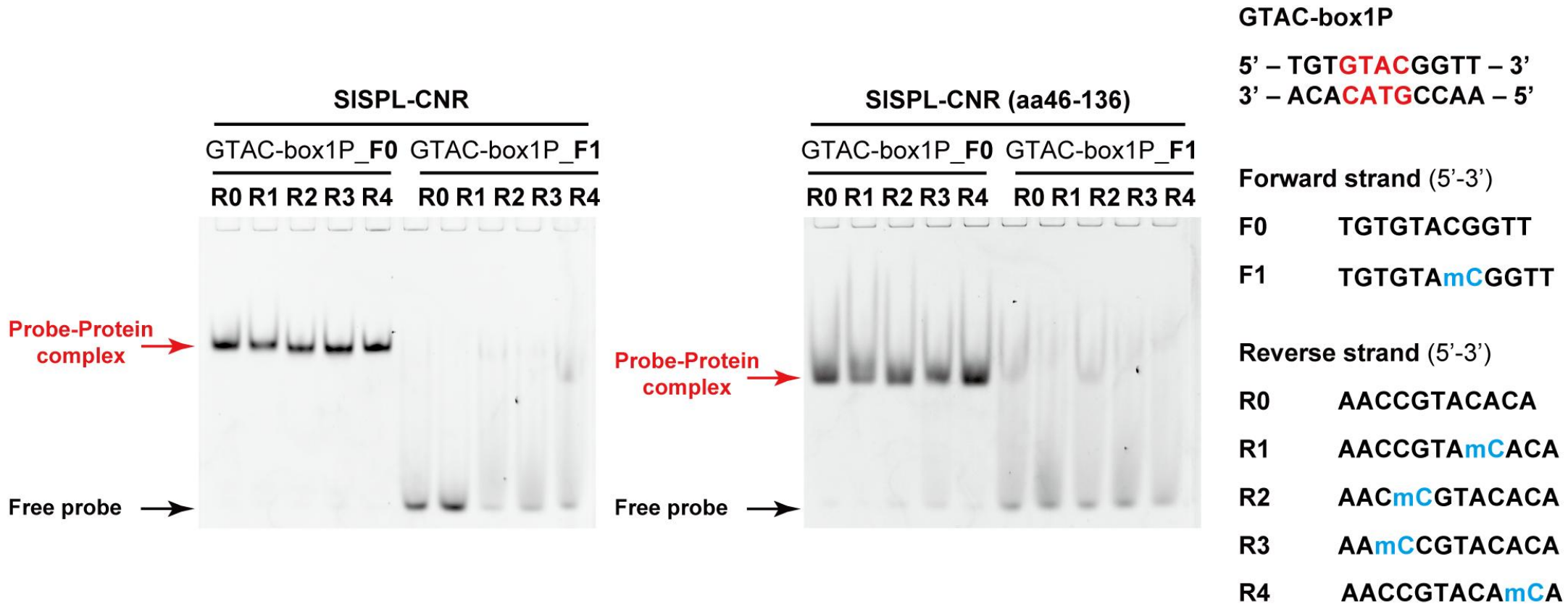

Supplementary Fig. 6 Binding affinity of SlSPL-CNR to GTAC-box1P with or without 5-mCs, related to Fig. 4. EMSA assessed SlSPL-CNR binding to GTAC-box1P probes with varying cytosine methylation states. 5-mC is indicated in blue "mC". SlSPL-CNR and its SBP domain bound strongly to unmethylated probes but showed markedly reduced or undetectable binding when one or more cytosines—especially C7 (F1)—were methylated. Experiments were repeated independently two times with similar results.

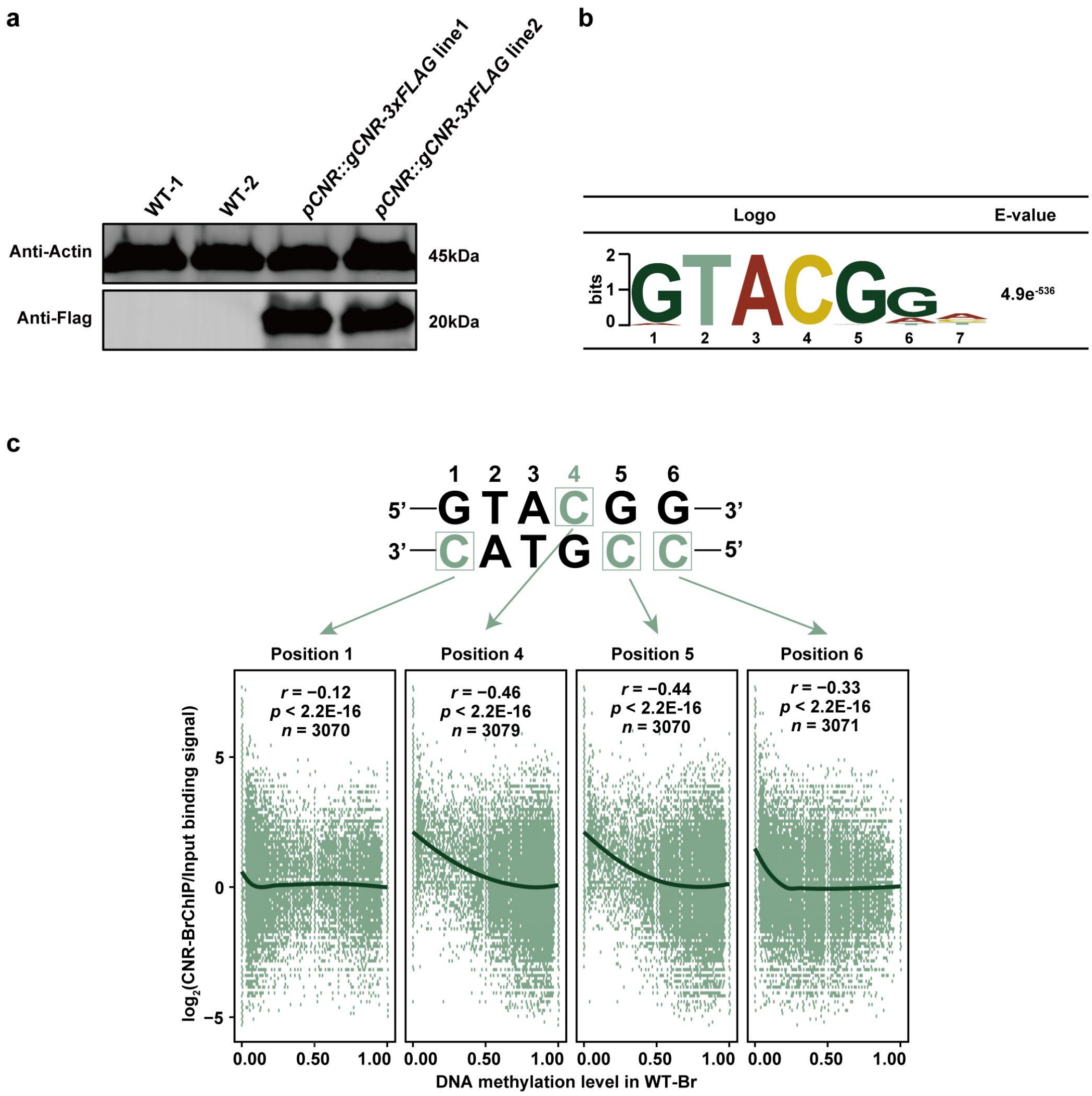

Supplementary Fig. 7 Characterization of SlSPL-CNR *in vivo* binding peaks captured by CNR-BrChIP, related to Fig. 5. a Western blot confirming SlSPL-CNR protein expression in WT and *pCBC::gCNR-3xFLAG* lines fruits at the breaker stage (38dpa). Experiments were repeated independently two times with similar results. **b** *De novo* motif analysis of CNR-BrChIP peaks reveals the GTACGG *cis*-element. The E-value is indicated. **c** Pearson correlation coefficients (r) between DNA methylation (WT-red) and $\log_2$(CNR-BrChIP/Input) binding signal ratios at individual cytosine positions within GTACGG motifs (n = 3,080 sites). P value was calculated using a Pearson correlation test. The numbers for each site are shown.

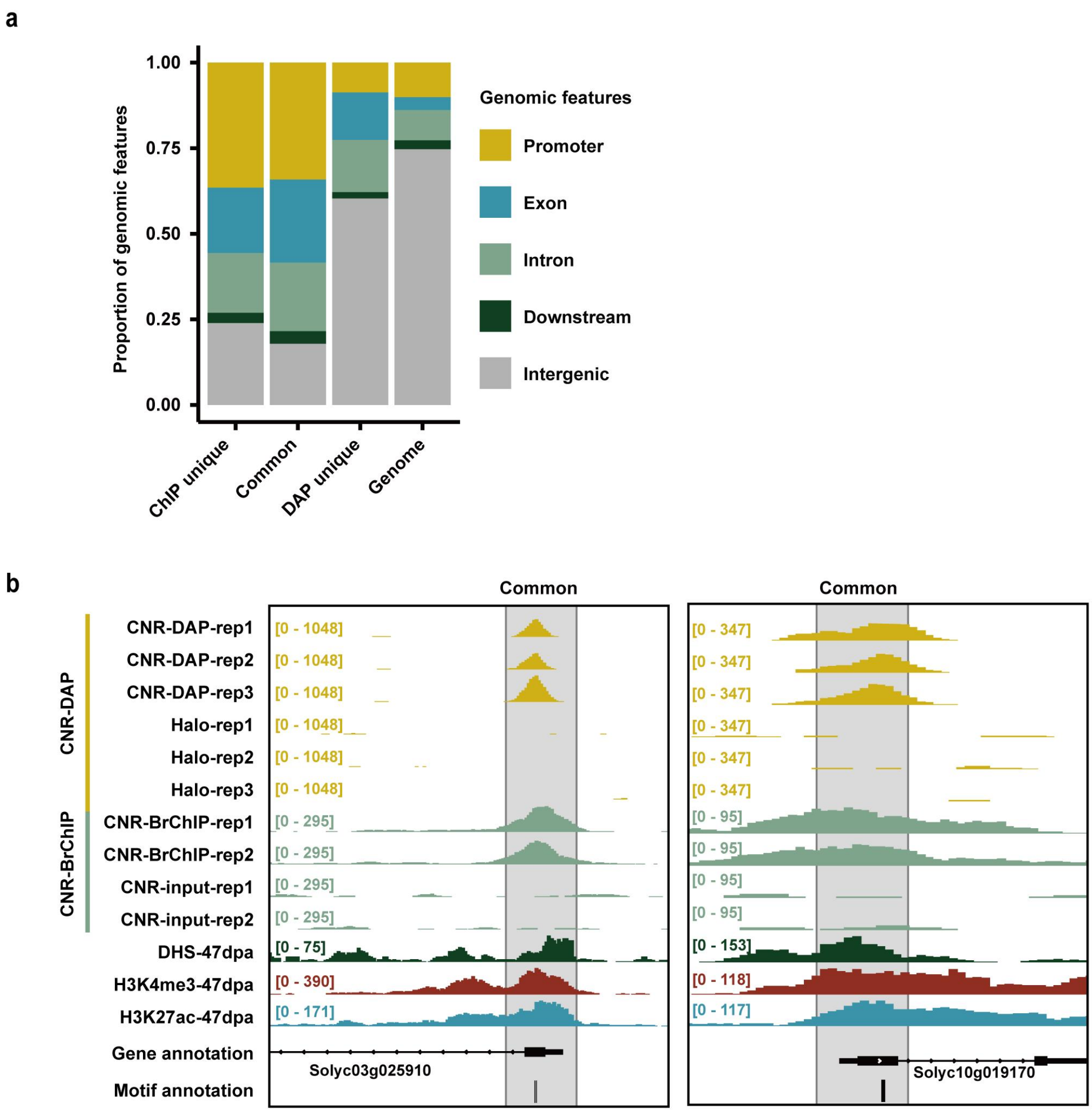

Supplementary Fig. 8 Impact of chromatin state on SlSPL-CNR binding profiles, related to Fig. 5. a Genomic distribution of ChIP unique (CNR-BrChIP only), DAP unique (CNR-DAP only) and common (ChIP-DAP common) peaks across annotated genomic features (promoter, exon, intron, downstream, and intergenic regions) relative to genome-wide composition (Genome). **b** Representative IGV tracks showing common peaks coincide with elevated chromatin accessibility (DHS) and active histone marks (H3K4me3, H3K27ac).

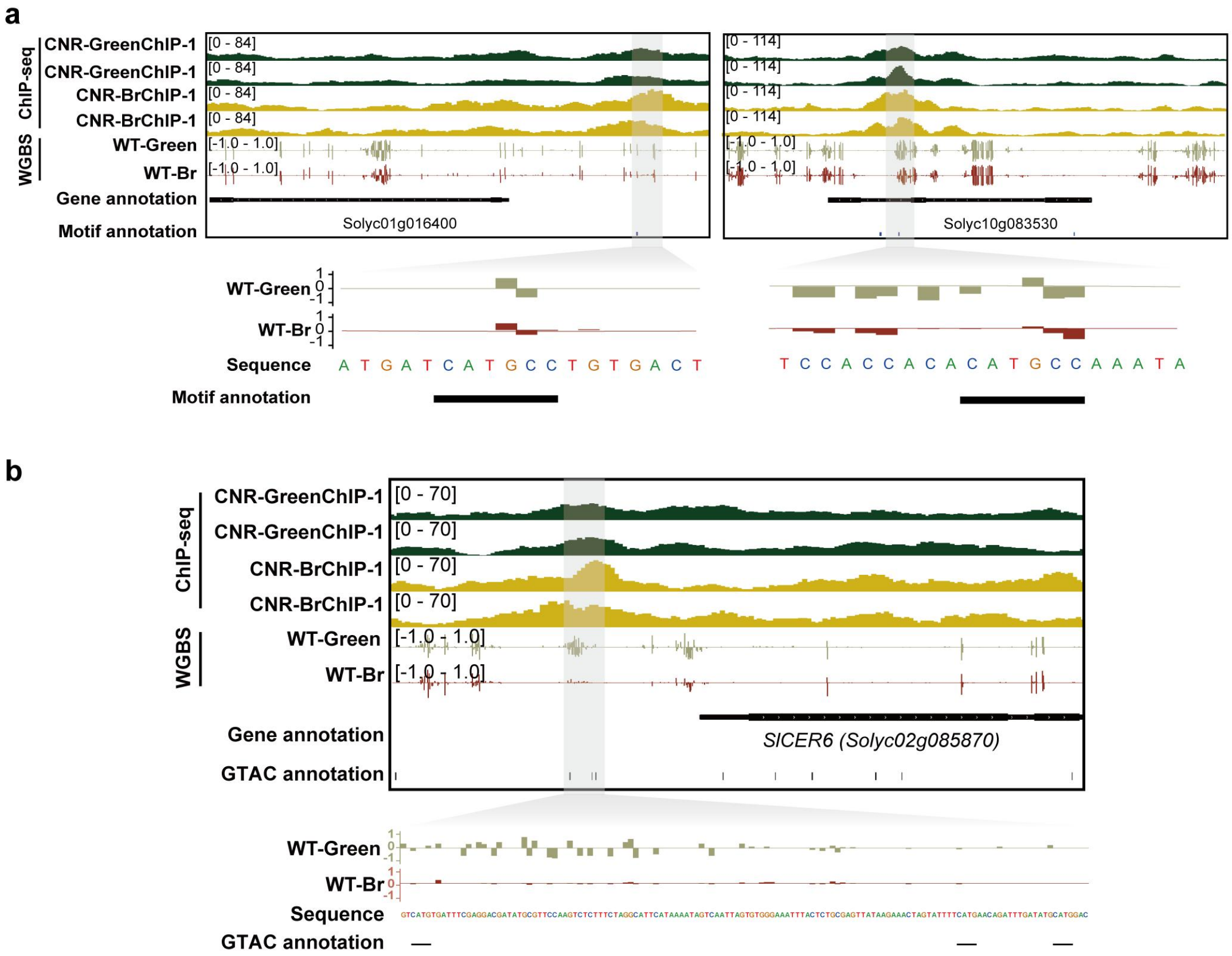

Supplementary Fig. 9 Representative *in vivo* genomic loci bound by SlSPL-CNR, related to Fig. 6. a IGV tracks showing SlSPL-CNR occupancy and DNA methylation at two representative hyper-C_4 site regions. **b** IGV visualization of SlSPL-CNR binding and DNA methylation patterns at the promoter region of the known target gene *SlCRE6*, with GTAC motifs and methylation status shown in magnified view.

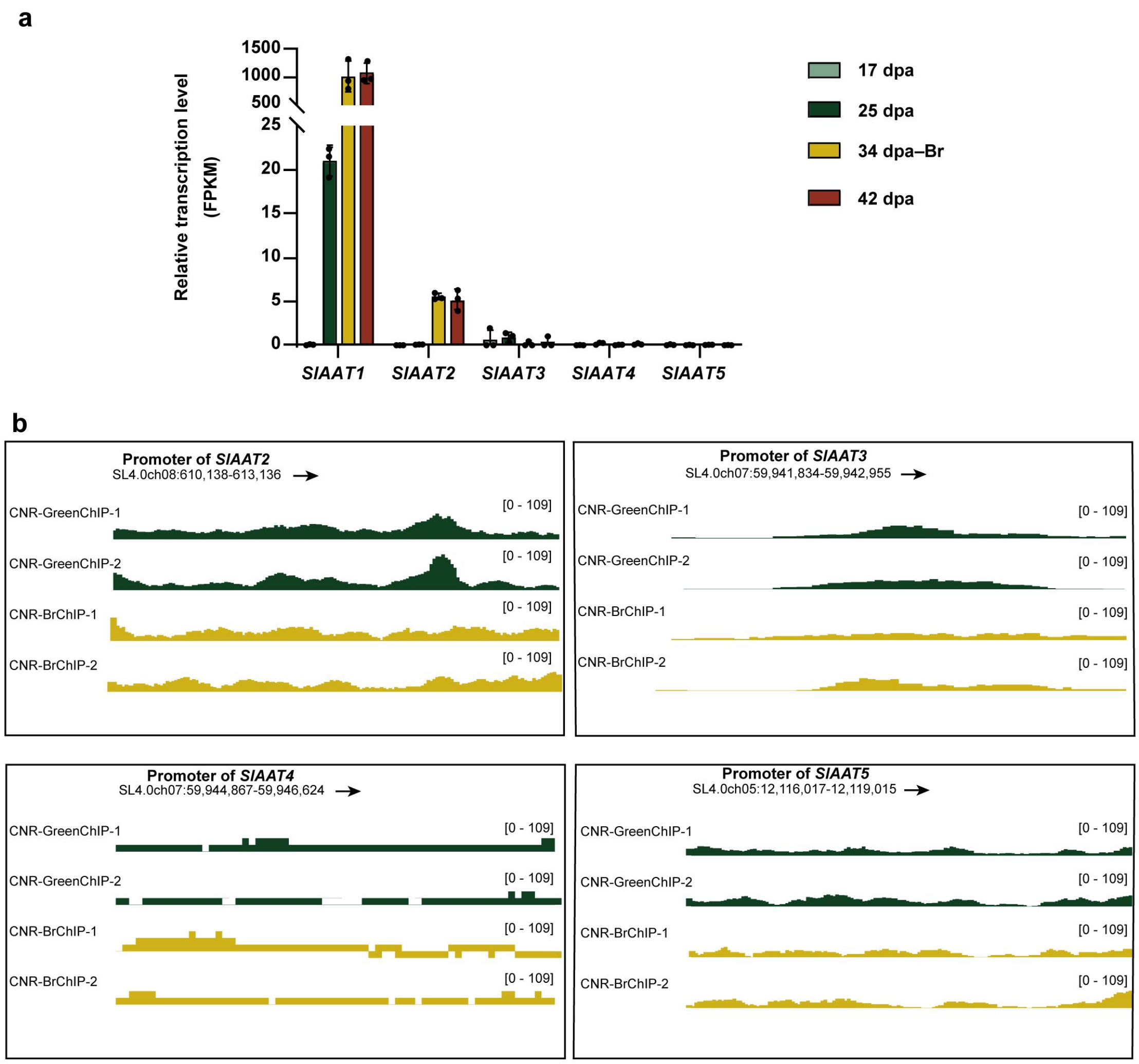

Supplementary Fig. 10 Transcriptional expression and SlSPL-CNR binding of selected *SlAAT* genes in tomato fruit, related to Fig. 6. a Transcript abundance (FPKM) of five *SlAAT* genes (*SlAAT1*–*SlAAT5*) in AC (wild-type) tomato fruit at at 17, 25, 34 (Breaker stage), and 42 dpa. Data are presented as mean ± SD (*n* = 3 biological replicates). Source data are provided in the Source Data file. **b** IGV visualization shows no SlSPL-CNR binding to the promoters of four *SlAAT* genes (*SlAAT2*–*SlAAT5*) in CNR-BrChIP. Source data are provided in a Source Data file.

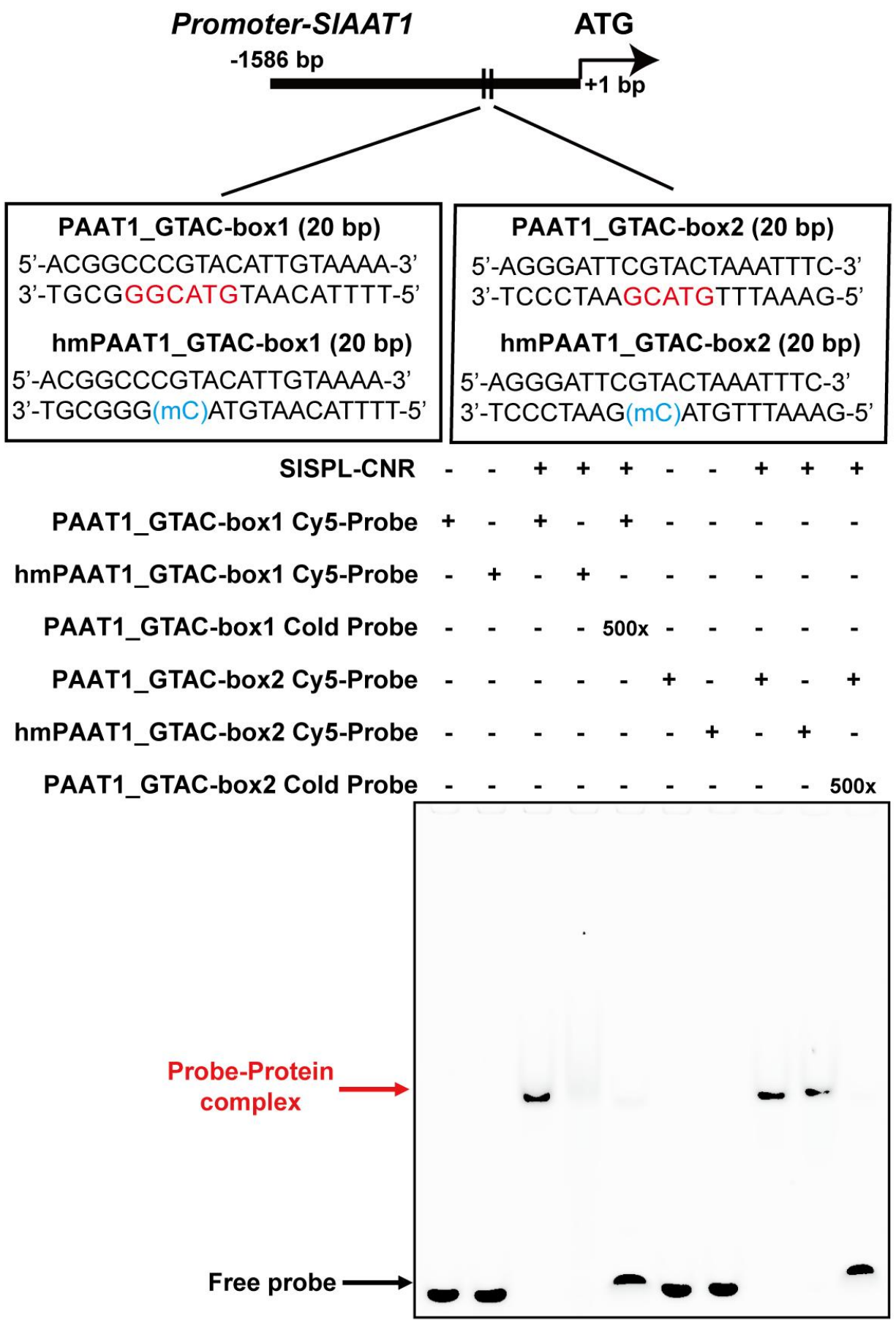

Supplementary Fig. 11 Methylation-sensitive binding of SlSPL-CNR to GTAC-box motifs in the *SlAAT1* promoter, related to Fig. 6. EMSA analysis of SlSPL-CNR binding to two 20-bp *SlAAT1* promoter probes: unmethylated (PAAT1_GTAC-box1, PAAT1_GTAC-box2) or hemi-methylated (hmPAAT1_GTAC-box1, hmPAAT1_GTAC-box2; 5-mC (blue) on the antisense strand). Full-length SlSPL-CNR was incubated with Cy5-labeled probes (1 μM), and binding specificity was validated by competition with a 500-fold excess unlabeled cold probe. "+" and "–" denote the presence or absence of the indicated protein or probe components. SlSPL-CNR binds robustly to both unmethylated GTAC-box motifs, while hemi-methylation completely abolishes or restrains this interaction, confirming methylation-sensitive DNA binding. Experiments were repeated independently two times with similar results.

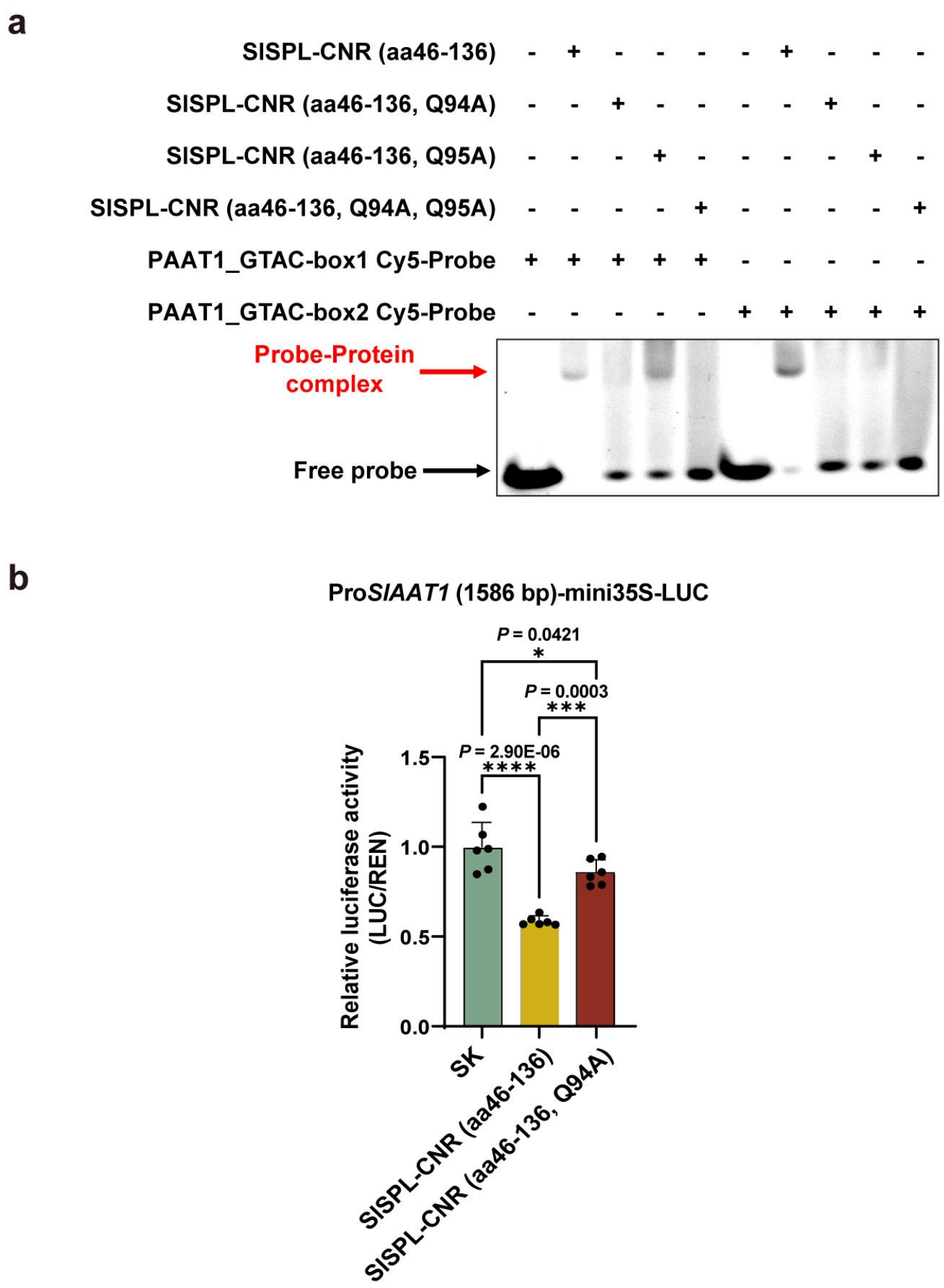

Supplementary Fig. 12 Site-directed mutagenesis of Gln94 in the SlSPL-CNR SBP domain impairs DNA binding and repressive activity toward the *SlAAT1* promoter, related to Fig. 6. a EMSA assessing the binding of SlSPL-CNR (aa46–136) and mutant proteins (Q94A, Q95A, Q94A/Q95A) to PAAT1_GTAC-box1 and PAAT1_GTAC-box2 probes. Wild-type SlSPL-CNR forms a stable probe-protein complex with both probes, while alanine substitutions at Gln94 or both completely abolish DNA binding. Experiments were repeated independently two times with similar results. **b** Dual-luciferase reporter assays showing that SlSPL-CNR (aa46–136) significantly represses the activity of the Pro*SlAAT1* (1586 bp) promoter, and the Q94A mutation markedly alleviates this inhibitory effect. Data are presented as means±SD from six biological replicates. Asterisks indicate statistical significance by one-way ANOVA followed by Tukey's multiple comparisons test (*$P < 0.05$, ***$P < 0.001$,****$P < 0.0001$). Source data are provided in the Source Data file.

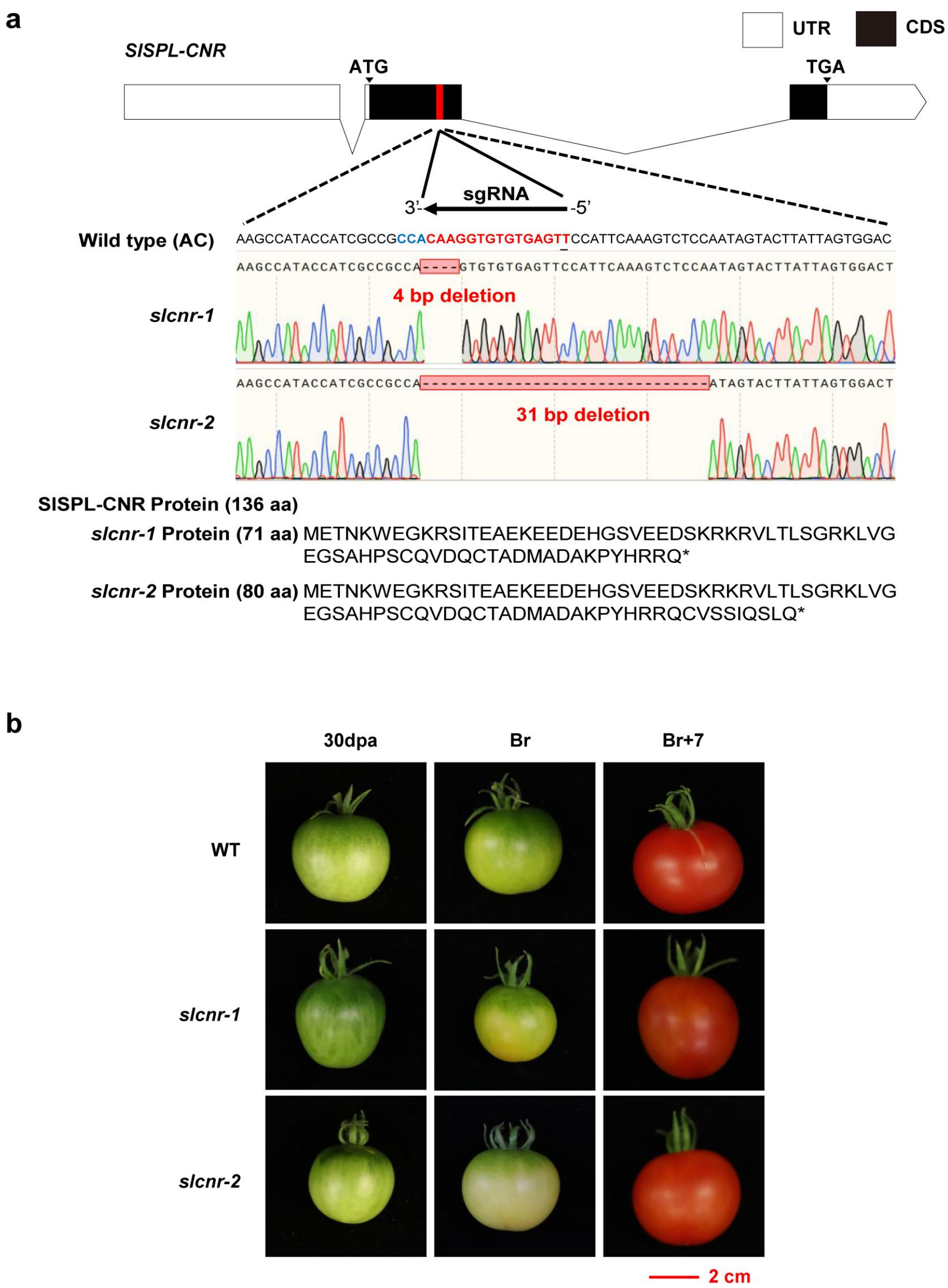

Supplementary Fig. 13 CRISPR/Cas9-mediated generation of SlSPL-CNR stable loss-of-function alleles, related to Fig. 6. a Targeted genomic region in wild-type (AC) and CRISPR-edited *slcnr* mutant lines (*slcnr-1*, *slcnr-2*), along with Sanger sequencing chromatograms. Second-generation transgenic plants (T1) were used for genotyping. Sequences highlighted in red indicate the guide RNA (sgRNA) targeting the first CDS region of SlSPL-CNR, with an arrow indicating its orientation for clarity. Blue letters denote the protospacer adjacent motif (PAM). Dashed red boxes highlight the editing sites: *slcnr-1* carries a homozygous 4-bp deletion, while *slcnr-2* has a homozygous 31-bp deletion. Protein sequences (WT: 136 aa; *slcnr-1*: 71 aa; *slcnr-2*: 80 aa) illustrate the frameshift consequences. **b** Fruit morphology of WT (AC) and *slcnr* mutant lines (*slcnr-1*, *slcnr-2*) fruits at three ripening stages (30 dpa, Br, and Br+7). Scale bar = 2 cm.

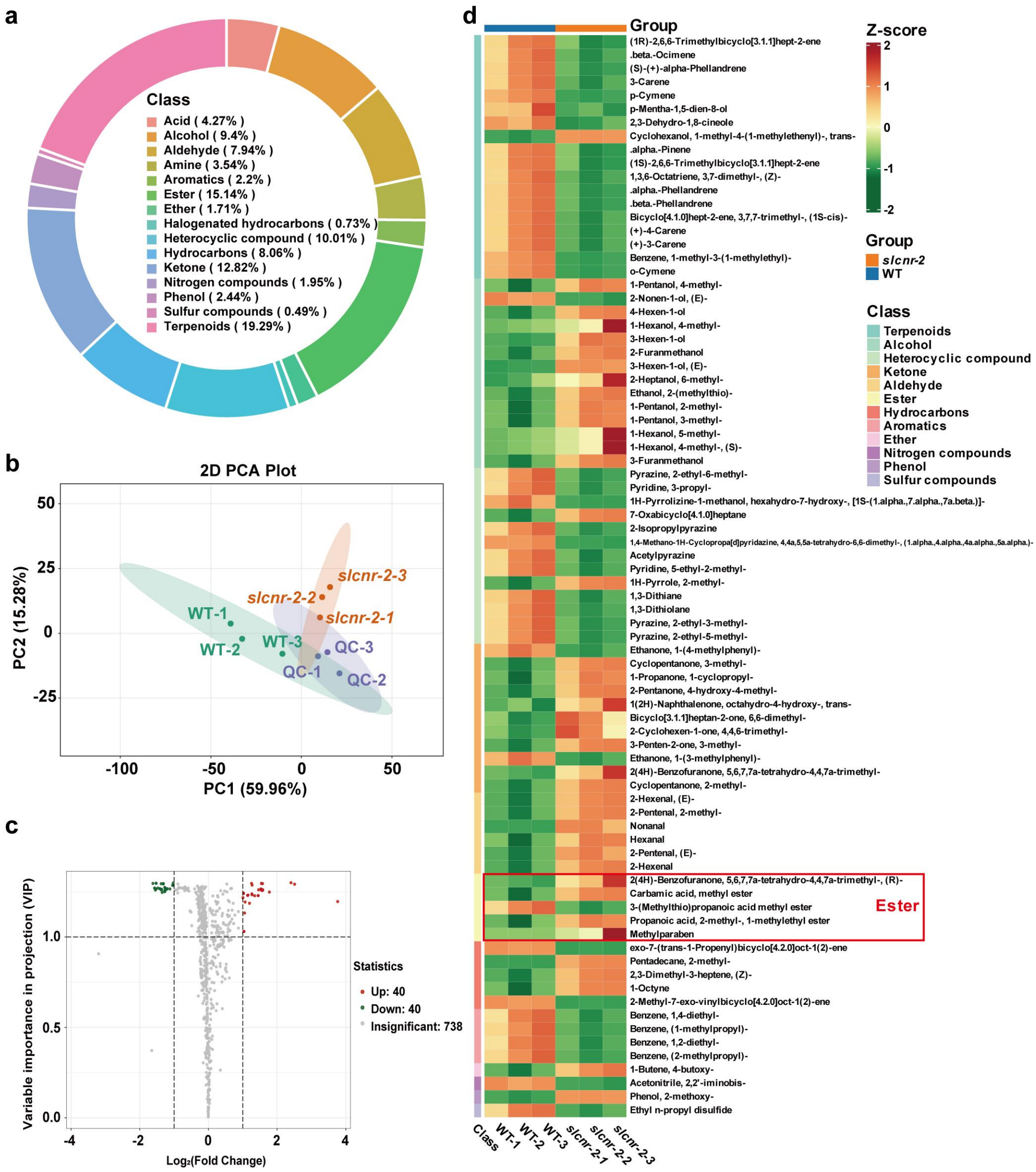

Supplementary Fig. 14 Metabolomic analysis of volatile organic compounds in WT and *slcnr-2* fruits at the breaker stage, related to Fig. 6. a Pie chart depicting the distribution of volatile metabolite categories across 15 classes. **b** Principal component analysis (PCA) plot illustrating sample segregation. **c** Differential VOCs identified between WT and *slcnr-2* using OPLS-DA modeling (n = 3 biological replicates). Metabolites with variable importance in projection (VIP) > 1 were selected as initial candidates, then filtered using fold change thresholds (FC $\geq$ 2 or $\leq$ 0.5). **d** Heatmap of 80 differential VOCs between WT and *slcnr-2*, showing relative abundance. Five differential ester compounds are highlighted (red box).

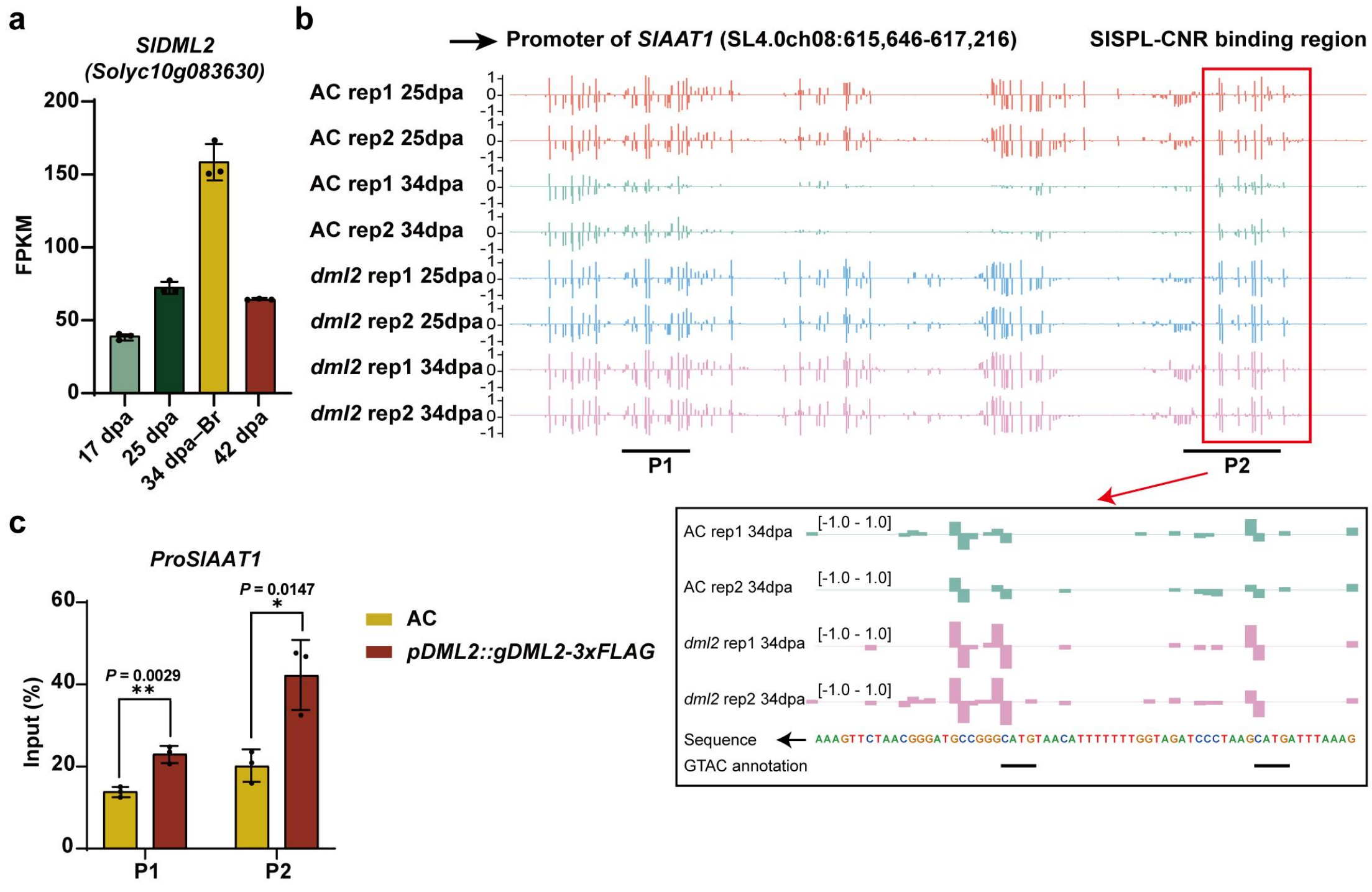

Supplementary Fig. 15 SlDML2 mediates the DNA demethylation at the *SlAAT1* promoter during fruit ripening, related to Fig. 6. a *SlDML2* transcript abundance (FPKM) in tomato WT fruits at 17, 25, 34 (Br stage), and 42 dpa. Data are presented as mean ± SD (*n* = 3 biological replicates). **b** IGV tracks showing DNA methylation patterns at the *SlAAT1* promoter in AC (WT) and *dml2* mutant fruits at 25 dpa and 34 dpa (Br stage), with enlarged view of GTAC motifs and their corresponding methylation levels at Br stage are shown below. Red box denotes hypermethylated differentially methylated sites within the SlSPL-CNR binding region. **c** ChIP-qPCR analysis of SlDML2 enrichment at *SlAAT1* promoter regions P1 and P2 in *pDML2::gDML2-3xFLAG* lines and AC control fruits at breaker stage. Data are presented as percentage of input DNA (means ± SD, *n* = 3 technical replicates). Asterisks denote statistically significant differences by two-tailed Student's t-test (*$P < 0.05$, **$P < 0.01$,), confirming significant enrichment of SlDML2 at both regions *in vivo*. Source data are provided in the Source Data file.

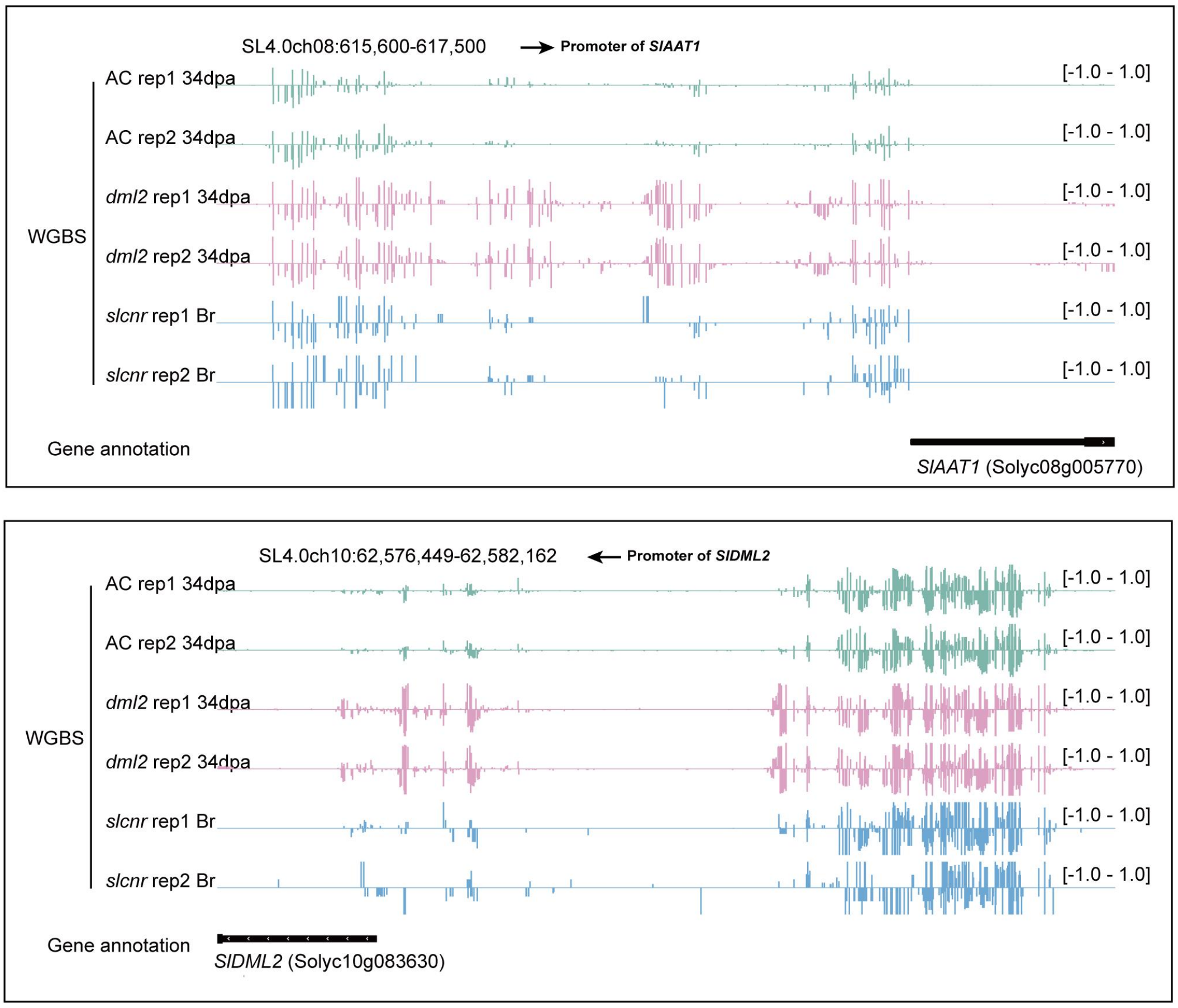

Supplementary Fig. 16 Methylation of *SlAAT1* and *SlDML2* promoters in *slcnr* mutant, related to Fig. 6. IGV visualization of WGBS data shows no significant differences in DNA methylation at *SlAAT1* and *SlDML2* promoters between wild-type (AC) and *slcnr* mutant fruit at the breaker (Br) stage.

a

Prediction of SlSPL-CNR—SlDML2 interaction by AlphaFold3

PPI	ipTM	pTM
SlSPL-CNR + SlDML2	0.24	0.39

b

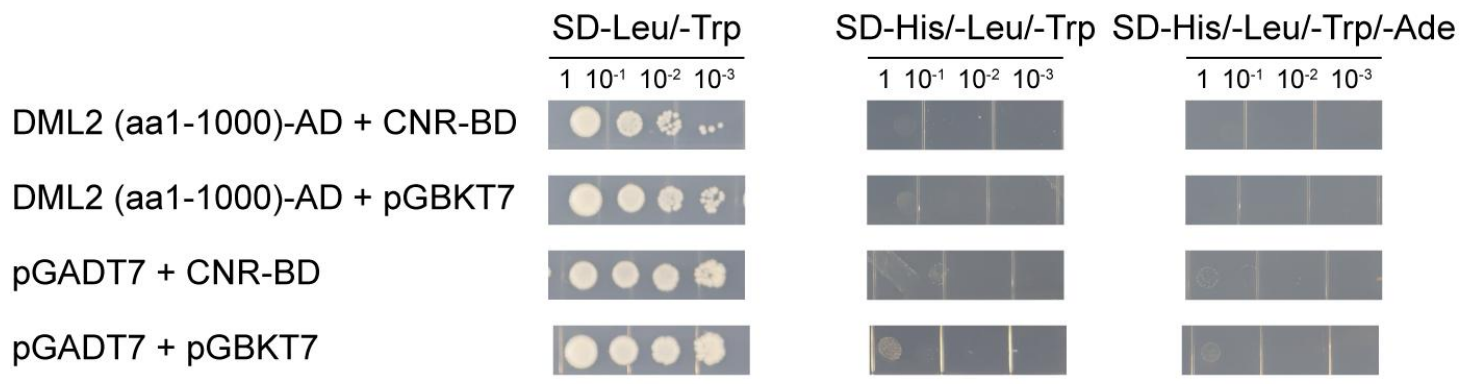

Supplementary Fig. 17 Assessment of physical interactions between SlSPL-CNR and SlDML2, related to Fig. 6. a AlphaFold3-based structural prediction of the protein–protein interaction (PPI) between SlSPL-CNR and SlDML2. Low predicted Template Modeling score (ipTM) of 0.24 (< 0.6) indicate no stable, high-confidence physical interaction between the two proteins. Prediction was performed via the AlphaFold Server (https://alphafoldserver.com). **b** Yeast two-hybrid assay confirms no direct interaction between SlDML2 (aa1-1000) and SlSPL-CNR, with no growth on selective media. Experiments were repeated independently two times with similar results.

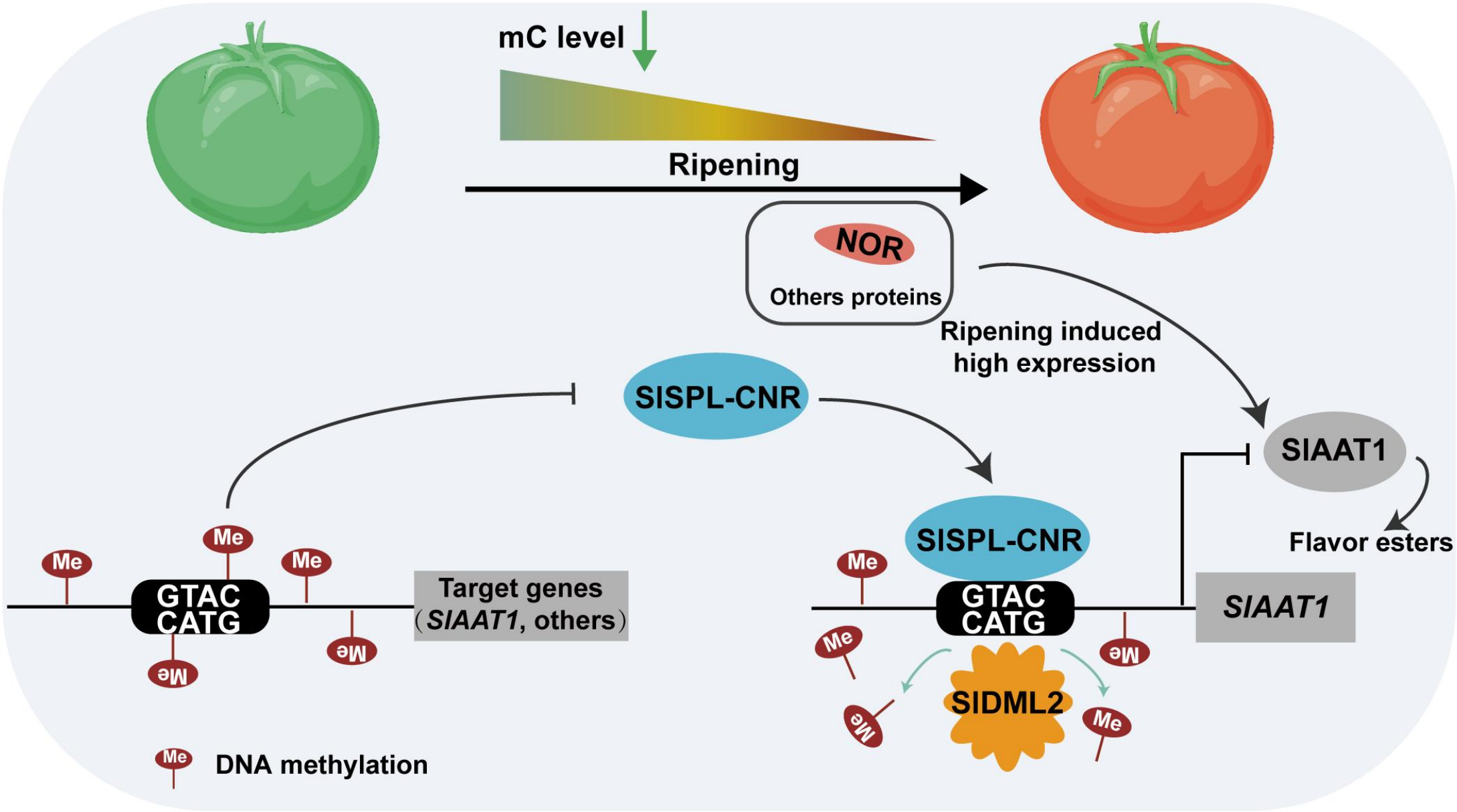

Supplementary Fig. 18 Schematic model of SlSPL-CNR-mediated negative feedback regulation of *SlAAT1* during tomato fruit ripening, related to Fig. 6. Ripening induces *SlAAT1* transcription—activated by NOR and other regulatory proteins—while simultaneously promoting *SlDML2*-mediated hypomethylation of the *SlAAT1* promoter, including GTAC motif, thereby enhancing SlSPL-CNR binding. Occupancy of SlSPL-CNR subsequently represses *SlAAT1* expression, establishing a feedback loop that modulates flavor esters metabolism and maintains expression within a range compatible with coordinated fruit quality development. The green-to-red gradient illustrates ripening progression; arrows denote regulatory interactions.

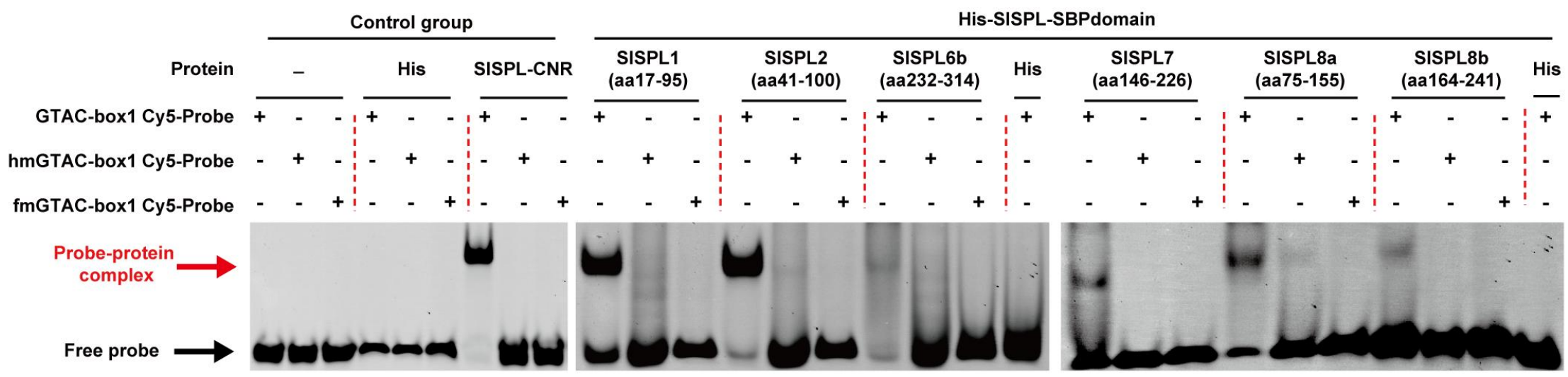

Supplementary Fig. 19 DNA methylation sensitivity of selected tomato SBP proteins, related to Fig. 7. EMSA assays evaluating SBP domains from six tomato SPL proteins binding to unmethylated, hemi-methylated, and fully methylated GTAC probes (GTAC-box1). His-tagged empty vector protein (His) served as the negative control. Experiments were repeated independently two times with similar results.
