# Supplementary material for: DNA hypomethylation enables the transcriptional repressor SlSPL-CNR to control fruit flavor ester biosynthesis
Source: Nat Commun. 2026 Jul 1;17:8168. doi: 10.1038/s41467-026-75181-8 (PMC13458263; doi:10.1038/s41467-026-75181-8)
Supplement: Supplementary file 4 [file 41467_2026_75181_MOESM2_ESM.pdf]

Description of Additional Supplementary Files

File Name: Supplementary Data 1
Description: Quality metrics of sequencing data processing in this study.

File Name: Supplementary Data 2
Description: Differential binding analysis of normalized peaks between CNR-DAP and CNR-ampDAP.

File Name: Supplementary Data 3
Description: Fitting parameters of the ITC experiment.

File Name: Supplementary Data 4
Description: Data collection and refinement statistics for crystal structure of CNR SBP domain in complex with DNA.

File Name: Supplementary Data 5
Description: Differential methylation and SlSPL-CNR binding analysis at C4 among 3,080 GTACGG cis-element binding sites identified in CNR-BrChIP peaks.

File Name: Supplementary Data 6
Description: Gene Set Enrichment Analysis (GSEA) of hypo-C4 sites.

File Name: Supplementary Data 7
Description: KEGG pathway enrichment analysis on genes associated with 68 leading-edge subset sites.

File Name: Supplementary Data 8
Description: Volatile metabolism detected in *slcnr-2* mutants and AC tomato fruits.

File Name: Supplementary Data 9
Description: 80 differential metabolites of volatile (VIP > 1 and $|log_2FC| \geq 1$) detected in *slcnr-2* mutants and WT (AC) tomato fruits.

File Name: Supplementary Data 10
Description: Primers and protein sequences used in this study.
